# Contribution of increased mutagenesis to the evolution of pollutants-degrading indigenous bacteria

**DOI:** 10.1371/journal.pone.0182484

**Published:** 2017-08-04

**Authors:** Tanel Ilmjärv, Eve Naanuri, Maia Kivisaar

**Affiliations:** Department of Genetics, Institute of Molecular and Cell Biology, University of Tartu, Tartu, Estonia; University of Cape Town, SOUTH AFRICA

## Abstract

Bacteria can rapidly evolve mechanisms allowing them to use toxic environmental pollutants as a carbon source. In the current study we examined whether the survival and evolution of indigenous bacteria with the capacity to degrade organic pollutants could be connected with increased mutation frequency. The presence of constitutive and transient mutators was monitored among 53 pollutants-degrading indigenous bacterial strains. Only two strains expressed a moderate mutator phenotype and six were hypomutators, which implies that constitutively increased mutability has not been prevalent in the evolution of pollutants degrading bacteria. At the same time, a large proportion of the studied indigenous strains exhibited UV-irradiation-induced mutagenesis, indicating that these strains possess error-prone DNA polymerases which could elevate mutation frequency transiently under the conditions of DNA damage. A closer inspection of two *Pseudomonas fluorescens* strains PC20 and PC24 revealed that they harbour genes for ImuC (DnaE2) and more than one copy of genes for Pol V. Our results also revealed that availability of other nutrients in addition to aromatic pollutants in the growth environment of bacteria affects mutagenic effects of aromatic compounds. These results also implied that mutagenicity might be affected by a factor of how long bacteria have evolved to use a particular pollutant as a carbon source.

## Introduction

Contamination of the environment with various organic xenobiotic compounds is a widespread problem throughout the world, particularly in industrialized areas. Among the most abundant environmental pollutants, aromatic compounds are of major concern because of their persistence and toxicity. Although many of these compounds are resistant to degradation, the large genetic plasticity and metabolic versatility of bacteria allows them to evolve the necessary catabolic activities to use aromatic compounds as the sole carbon and energy source. New catabolic pathways evolve rapidly as a result of horizontal transfer of genes and point mutations that broaden the substrate range of pre-existing enzymes [1–6].

Most mutations are likely to be deleterious, and so the spontaneous mutation rate is generally held at a low level [7]. However, bacterial populations with higher mutation rates can adapt to novel environments faster than those with lower mutation rates [8]. The mutation rate can be elevated either constitutively (constitutive mutators) or transiently, in response to DNA damage or other stress situations [9, 10]. Transiently increased mutability could be the consequence of the action of the specific low-fidelity DNA polymerases, e.g., Pol IV, Pol V and DnaE2 [11, 12]. Depending on the type of function that is defective, constitutive mutators can have mutation rates that are moderately (about 10-fold) to strongly (100-1000-fold) increased [10]. The most potent constitutive mutators are bacteria defective in either the DNA polymerase III proofreading subunit or the methyl-directed mismatch repair system [13]. Mutators are expected to be rare in well-adapted populations [10]. However, surveys of natural isolates of pathogenic and commensal bacteria have revealed that constitutive mutators exist in the environment at much higher frequencies [14–16]. Subsequent studies [reviewed in [17, 18]] demonstrated that hypermutators can be readily isolated from populations of a variety of human pathogens at frequencies ranging from 1% to 58% of total population. The high proportion of mutators among clinical isolates can be explained by advantage that they give in generating the variability pool for selecting for mutations that could be beneficial under harsh environmental conditions bacteria meet during pathogenesis. By selecting a favourable allele, natural selection indirectly selects the DNA in which this mutation occurred. Consequently, mutator alleles can spread in microbial populations by hitchhiking: mutators can acquire favourable alleles more frequently than nonmutators and will therefore increase in frequency if the advantage of beneficial alleles is greater than the cost of being a mutator [19]. So far, surveys of constitutive mutators have mostly included human pathogens. Whether the survival and evolution of organic pollutants degrading indigenous bacteria could also be connected with mutator phenotype is still unexplored.

Above a certain threshold organic compounds are toxic to bacteria as they damage cell membrane and impair biosynthetic pathways [20, 21]. Membrane stress is connected with generation of reactive oxygen species (ROS) [22, 23]. Notably, exposure of *Pseudomonas putida* to toluene induces transcription of *uvrA* and *uvrB* genes that are involved in nucleotide excision repair (NER) [22], which indicates that the exposure of bacteria to pollutants could induce DNA damage. Thus, exposure of bacteria to aromatic pollutants and aliphatic solvents may facilitate generation of new catabolic pathways due to their potential mutagenic effects. This idea has been strongly supported by the recent finding that the faulty dioxygenation reaction of the evolving enzyme generates ROS, which, in turn, elevates mutation frequency in the 2,4-dinitrotoluene-degrading bacteria [6].

The aim of the current study was to explore mechanisms of evolution of xenobiotics degrading indigenous bacteria. Specifically, we assayed for the presence of constitutive and transient mutators among pollutants degrading bacteria, many of them isolated from heavily contaminated environments.

## Results

### Estimation of spontaneous mutation frequency among pollutants-degrading indigenous strains disclosed rare appearance of constitutive mutators

**Fig 1:**
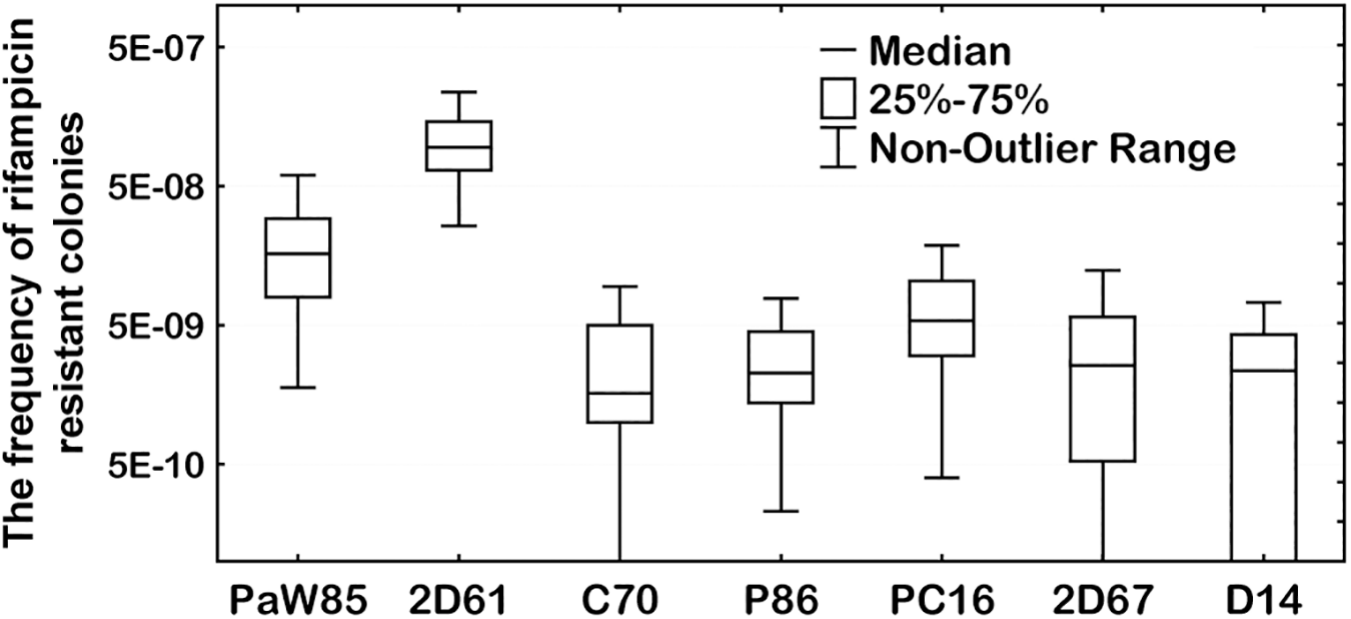
Median values of the spontaneous Rif^r^ mutant frequencies of the indigenous strains and the laboratory reference strain PaW85. Presented are only the results for strains exhibiting the mutant frequency statistically significantly different from that of the reference strain PaW85 (the Kruskal-Wallis test, p<0.006; S1 Table). Isolate D2RT is missing from the figure because the median value of the frequency of Rif^r^ mutants of this strain was 0 (the mean value of Rif^r^ frequency of this strain was 1.07 x 10^−9^).

In order to examine the possibility that the evolution of xenobiotic degrading bacteria could be connected with the appearance of constitutive mutators, we estimated the spontaneous mutation frequencies of 53 indigenous strains, mostly pseudomonads, which were able to degrade various pollutants, e.g., alkanes and phenolic compounds (Table 1). These strains were originated from the Baltic Sea and from the river water polluted with phenolic compounds of the oil shale industry leachate in Northeast Estonia [24–26]. In order to search for constitutive mutators, we employed the *rpoB*/Rif^r^ test system which has been commonly used to estimate the frequency of mutations in Gammaproteobacteria, e.g., in *Escherichia coli* [27] and in pseudomonads [28]. Specifically, we performed fluctuation tests and determined the frequency of rifampicin-resistant mutant colonies/ml with regard to the total viable colony count/ml of the same culture. Spontaneous Rif^r^ mutant frequency of the indigenous strains was compared with that of the laboratory reference strain *P*. *putida* PaW85. *P*. *putida* strain PaW85 [29] is a plasmid-free derivative of the toluene catabolic plasmid pWW0-harbouring strain mt-2 (PaW1) [30]. This strain is isogenic to the strain KT2440 which is widely used in laboratory experiments [31]. According to our previous studies [28] PaW85 has a modal or so called “normal” Rif^r^ mutant frequency. The median value of the Rif^r^ mutant frequency (*f*) of PaW85 estimated in the current study was 1.6 x 10^−8^. Approximately 87% of the studied indigenous isolates had the Rif^r^ mutant frequency statistically similar to that of the reference strain PaW85. Only two of the studied strains were moderate mutators with considerably higher mutant frequency in comparison with PaW85 (Fig 1 and S1 Table). Specifically, *P*. *stutzeri* strain 2C23 and *P*. *putida* strain 2D61 exhibited about 15- and 6-fold higher Rif^r^ frequency than the reference strain *P*. *putida* PaW85 (p = 0.0094 and p<0.0001, respectively). However, according to the Benjamini-Hochberg procedure only the strain 2D61 had statistically higher mutant frequency. Notably, out of all studied strains about 11% (6 strains) were hypomutators. The hypomutators were represented among most of the studied species except *P*. *stutzeri*.

**Table 1:** The bacterial strains used in this study.

Laboratory reference strains			
Strain	Species	Characteristics:	Source or reference
PaW1	*P*. *putida*	Carries toluene catabolic plasmid pWW0	[29]
PaW85	*P*. *putida*		[29]
PaWrulAB	*P*. *putida*	PaW85 carrying the *rulAB* genes from pWW0 in chromosome	[35]
PaWpheBA25	*P*. *putida*	PaW85, carries the phenol degradation genes (*pheBA*) in the chromosomal locus PP2556	[111]
Indigenous isolates			
Strain^a^	Species	Pollutant degradation capability^b^	Source or reference
2A20	*P*. *stutzeri*	Phe, Ben, mTol	[25]
2A38	*P*. *stutzeri*	Phe, Ben, mTol, Sal, Nah	[112]
2A54	*P*. *stutzeri*	Ben, mTol	[25]
2Anah4	*P*. *stutzeri*	Phe, Ben, mTol	[112]
2B45	*P*. *stutzeri*	Phe, Ben, mTol	[112]
2C23	*P*. *stutzeri*	Phe, Ben, mTol, Nah	[112]
2C41	*P*. *stutzeri*	Phe, Ben, mTol	[112]
2C56	*A*. *johnsonii*	Phe, Ben, mTol, Nah	[25]
2C63	*P*. *stutzeri*	Phe, Ben, mTol	[112]
2D47	*P*. *stutzeri*	Ben, mTol, Sal	[25]
2D61	*P*. *putida*	Ben, mTol, Tol, Phe, Cre, pHBA, PCat, Sal	[25]
2D66	*P*. *stutzeri*	mTol, Tol	Unpublished
2D67	*A*. *johnsonii*	Phe, Ben, mTol, Nah	[25]
C52	*P*. *stutzeri*	Phe, Ben, mTol	[112]
C70	*P*. *pseudoalcaligenes*	Phe, Ben, Sal, Nah	[25]
D113	*P*. *fluorescens*	Alkanes (Df)	[26]
D14	*Acinetobacter sp*.	Phe, Ben, mTol, Nah	[25]
D28	*P*. *fluorescens*	Alkanes (Df)	[26]
D2RT	*P*. *migulae*	Ben, Sal, mTol,	[25]
D3	*P*. *fluorescens*	Alkanes (Df)	[26]
D45	*Pseudomonas sp*.	Alkanes (Df)	[26]
D66v	*A*. *johnsonii*	Phe, Ben, mTol, Nah	[112]
D67	*P*. *migulae*	Ben, Sal, mTol,	[25]
Hd1	*P*. *fluorescens*	Alkane (C16)	[26]
Hd16	*P*. *fluorescens*	Alkane (C16)	[26]
Hd6	*P*. *fluorescens*	Alkane (C16)	[26]
Hp2	*P*. *fluorescens*	Alkane (C7)	[26]
Hp5	*Pseudomonas sp*.	Alkane (C7)	Unpublished
Hp6	*P*. *fluorescens*	Alkane (C7)	[26]
Nah4	*Shewanella sp*.	Nah	Unpublished
P3	*P*. *fluorescens*	Alkanes (OO)	[26]
P4	*P*. *fluorescens*	Alkanes (OO)	[26]
P48	*P*. *fluorescens*	Alkanes (OO)	[26]
P49	*P*. *fluorescens*	Alkanes (OO)	[26]
P6	*P*. *fluorescens*	Alkanes (OO)	[26]
P69	*P*. *fluorescens*	Phe_*pheA*_	[45]
P85	*P*. *fluorescens*	Alkanes (OO)	[26]
P86	*P*. *fluorescens*	Alkanes (OO)	[26]
P87	*P*. *fluorescens*	Alkanes (OO)	[26]
P94	*P*. *fluorescens*	Alkanes (OO)	[26]
PC13	*P*. *putida*	Phe_*pheA*_	[24]
PC14	*P*. *putida*	Phe	[24]
PC15	*P*. *putida*	Phe	[24]
PC16	*P*. *putida*	Phe_*pheA*_	[24]
PC17	*P*. *fluorescens*	Phe, Sal, pCre	[24]
PC18	*P*. *fluorescens*	Phe, pCre	[24]
PC20	*P*. *fluorescens*	Phe_*pheA*_, Sal, Nah, Ben, mTol, pCre	[24]
PC24	*P*. *fluorescens*	Alkanes (C7, C16), Phe_*pheA*_, Sal, Nah, pCre	[24]
PC30	*P*. *putida*	Phe	[24]
PC34	*P*. *fluorescens*	Phe	[24]
PC36	*P*. *fluorescens /*	Phe	[24]
PC38	*P*. *fluorescens*	Phe	[24]
PC39	*P*. *putida*	Phe_*pheA*_	[24]

^a^All indigenous strains are deposited in the Collection of Environmental and Laboratory Microbial Strains (CELMS; http://eemb.ut.ee).

^b^Abbreviations: Ben—benzoate; C7—heptane; C16—hexadecane; Cre—cresol; Df—diesel fuel; pHBa—*p*-hydroxybenzoate; Nah—naphthalene; OO—shale oil; PCat—protocatechuate; Phe–phenol; Phe_*pheA*_—phenol is degraded by the single-component phenol hydroxylase, encoded by the *pheA* gene; Sal—salicylate; mTol—*m*-toluate; Tol—toluene.

To evaluate the results obtained with the *rpoB*/Rif^r^ assay, we repeated the estimation of the mutant frequency by using another assay which monitored the appearance of spontaneous streptomycin resistant mutants (Sm^r^ assay; S1 Fig). In addition, we excluded a possibility that the estimated mutant frequencies of the studied environmental isolates could be affected by variations in the tolerance to Rif or Sm by measuring the minimal inhibitory concentrations (MIC) (S2 Table). Representatives from different species either exhibiting the highest or the lowest Rif^r^ mutant frequency in comparison with the other strains from the same species were included in this assay. In general, the strains with the highest Rif^r^ mutant frequency exhibited also an elevated Sm^r^ mutant frequency (S1 Fig). This assay also disclosed the absence of strong mutators among the studied indigenous strains. Notably, although the Rif^r^ and Sm^r^ mutant frequencies of the reference strain PaW85 were comparable, the majority of the studied indigenous strains displayed statistically significantly lower frequency of Sm^r^ mutants in comparison to the frequency of Rif^r^ mutants (S1 Fig, S3 and S4 Tables). Taken together, the absence of strong mutators among the studied indigenous strains implied that constitutive mutability could have only a minor role on the evolution of pollutants-degrading indigenous strains.

### Indigenous isolates harboured DNA damage-induced mutagenic pathways

In addition to the constitutive pathway increased mutability can be transient and inducible, for example, through the action of specific low-fidelity DNA polymerases [11, 18]. One of the best studied is the UV irradiation-inducible DNA polymerase Pol V-dependent mutagenic pathway encoded by the *umuDC* operon in *E*. *coli* [32–34]. In many environmental bacteria, e.g., in *P*. *putida* and *P*. *syringae* the Pol V genes (usually named as *rulAB* genes) are frequently located on plasmids [18]. The translesion DNA synthesis (TLS) provided by the mutagenic DNA polymerase Pol V grants bacteria higher tolerance to UV-C irradiation and to several other DNA-damaging agents [32, 34]. For instance, we have previously shown that the insertion of the *rulAB* mutagenic operon from the toluene catabolic plasmid pWW0 into the chromosome of *P*. *putida* PaW85 (strain PaWrulAB) increased approximately 10 times the tolerance against the UV-C irradiation at a dose of 50 J/m^2^ [35]. The UV tolerance and mutability phenotype is widely distributed among various bacterial species [18, 36]. Usually there are no quantitative data available how frequent this phenotype is. However, it was reported that about 50% of *Pseudomonas syringae* pathovars isolated from plants throughout the world expressed the UV tolerance phenotype and carried the *rulAB* genes [37]. Therefore, we expected that some of the indigenous bacterial strains characterized in this study could also harbour DNA damage-inducible mutagenesis pathways. Since the higher tolerance to UV-C irradiation could hint to the presence of DNA damage-induced mutagenic pathways, we estimated the UV tolerance of the isolated strains. The survival rate of bacteria was measured after the exposure of cells to the UV-C light at doses of 10 J/m^2^ and 20 J/m^2^. Two *P*. *putida* strains, PaWrulAB and PaW1, both of which possessed the Pol V-encoding *rulAB* operon, were used as positive controls in the UV tolerance experiments.

**Fig 2:**
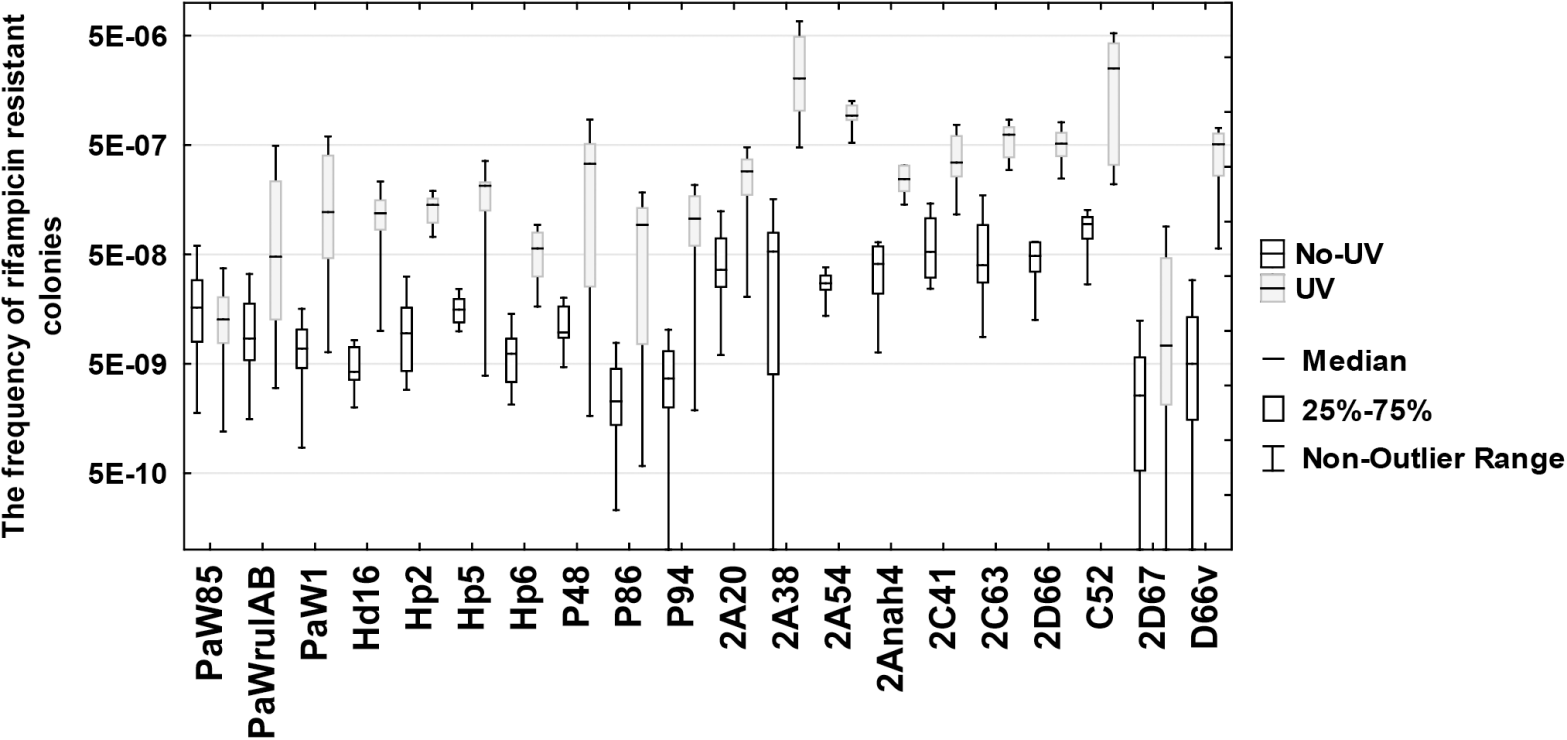
Comparison of spontaneous and UV-induced Rif^r^ mutant frequencies. Presented are only the results with indigenous strains exhibiting statistically significantly increased mutation frequency (p<0.045) after the exposure to UV-C irradiation analysed with Mann-Whitney U test (S6 Table).

We observed that ~89% of the studied strains (41 strains from the total of 46 investigated) had statistically similar tolerance to the UV irradiation (20 J/m^2^) in comparison with the strain PaW1 (Table 2 and S5 Table). In addition to *P*. *stutzeri* we were able to detect bacterial isolates with increased UV tolerance among the species of *P*. *fluorescens* and *Acinetobacter johnsonii*. All *P*. *putida* indigenous isolates exhibited lower UV tolerance than the *P*. *putida* laboratory strains PaWrulAB and PaW1 (S5 Table). As already mentioned above, the strains with increased tolerance against the UV irradiation might be potential candidates for possessing DNA damage-induced mutagenic pathways. In order to evaluate this possibility, we measured the UV mutagenesis in the strains which had at least 10-fold higher tolerance to the UV irradiation in comparison with the reference strain PaW85. Bacteria were treated by UV-C irradiation at a dose of 100 J/m^2^ which has been previously used in the UV mutagenesis assay [35]. *P*. *putida* strains PaWrulAB and PaW1 were used as positive controls. Under such conditions the exposure of the reference strain PaW85 to the UV irradiation had no increasing effect on the frequency of Rif^r^ mutants (Fig 2, p>0.05). At the same time, the presence of the *rulAB* operon increased the Rif^r^ mutant frequency more than 10 times, which was even higher than that observed in our previous experiments [35]. About 90% of the studied indigenous isolates with the increased UV tolerance expressed also the UV mutagenesis phenotype (Fig 2 and S6 Table). In addition, we estimated the effect of the UV irradiation on the Rif^r^ mutant frequency in the case of 6 strains (strains PC16, PC18, PC20, PC24, D14, and 2D61) which exhibited lower tolerance against the UV irradiation. It turned out that 3 strains (PC16, PC18, and PC24) exhibited the UV mutagenesis phenotype (S6 Table). Thus, our results indicated that at least one third of the indigenous strains examined in this study have a potential to evolve via transient mutability. However, the real proportion of such strains could be much larger since based on our results the increased UV tolerance seems to be not the only indicator of the presence of mutagenic pathways in the studied isolates.

**Table 2:** UV-C irradiation tolerance of indigenous strains.

The percentage of survival after UV-irradiation^a^			
Dosage J/m^2^	10	20	
PaW85^b^	16.2 (22.5)	0.2 (0.4)	*Pseudomonas putida*
PaWrulAB	47.8 (38.5)	5.1 (5.4)	
PaW1	46.3 (25.0)	8.1 (7.9)	
2A20	26.1 (14.1)	5.8 (3.6)	*Pseudomonas stutzeri*
2A38	81.1 (34.2)	94.9 (30.3)	
2A54	83.3 (33.5)	44.9 (16.9)	
2ANah4	41.0 (24.1)	18.9 (11.7)	
2C23	32.6 (10.8)	13.4 (11.2)	
2C41	55.8 (35.4)	64.0 (33.0)	
2C63	46.3 (22.9)	27.8 (17.5)	
C52	89.8 (39.9)	58.7 (34.4)	
2D66	44.0 (22.7)	28.8 (20.6)	
Hp2	47.0 (41.7)	13.8 (14.1)	*Pseudomonas fluorescens*
Hp5	46.0 (29.3)	7.86 (4.9)	
Hp6	37.5 (14.9)	10.88 (4.6)	
P48	42.1 (41.5)	10.7 (6.7)	
P86	35.67 (24.9)	8.4 (6.3)	
P94	48.1 (18.3)	7.5 (6.7)	
PC17	38.3 (34.9)	5.3 (5.5)	
Hd16	34.8 (11.5)	4.2 (1.5)	
PC20^b^	21.1 (11.1)	1.5 (1.9)	
PC24^b^	3.7 (3.4)	0.1 (0.01)	
2D67	60.8 (48.0)	36.9 (47.5)	*Acinetobacter johnsonii*
D66v	59.4 (76.6)	31.0 (20.7)	

^a^The percentages of survival after UV-C irradiation are shown only for strains with at least 10 fold higher UV-C tolerance compared to the reference PaW85. Each data represents the mean from three replicate UV-tolerance experiments. Standard deviation is added in the parentheses.

^b^Strain which exhibited statistically significantly lower UV-tolerance than PaW1.

### The genomes of *P*. *fluorescens* strains PC20 and PC24 possess several copies of Pol V genes and a copy of *imuC*

Among the strains investigated in this study, *P*. *fluorescens* strains PC20 and PC24 have been shown to have a great potential for application in bioaugmentation and rhizoremediation [38, 39]. Therefore, to further explore the catabolic potential of PC20 and PC24, the genomes of these strains have been sequenced in Ain Heinaru’s laboratory. The availability of the draft sequence data enabled us a closer inspection for the presence of error-prone DNA polymerases in these strains. Importantly, in a number of bacterial species which do not have the *umuDC* genes or their orthologs (e.g., *rulAB*) in the chromosome, carry instead another DNA damage-regulated operon, originally named as *imuABdnaE2* operon (renamed later as *imuABC* operon), which encodes for a mutagenic paralog of the replicative DNA polymerase Pol III, named as DnaE2 or ImuC [40]. This DNA polymerase has been shown to be responsible for the UV tolerance and UV-induced mutagenesis in various bacterial species, e.g., in *Mycobacterium tuberculosis* and in *Caulobacter crescentus* [40–42]. The DNA damage-inducible *imuABC* operon has been identified also in pseudomonads [43]. Therefore, we decided to analyse the sequence data of the strains PC20 and PC24 not only for the presence of genes encoding for Pol V but also for the presence of ImuC. We found that both strains harboured the *imuABC* operon. Surprisingly, the strain PC20 possessed additionally three copies of the *rulAB* operon, two in the chromosome and the third one was localized on the plasmid pG20 (unpublished data, GenBank accession number KX893538). The strain PC24 carried two copies of the *rulAB* operon in the chromosome.

**Fig 3:**
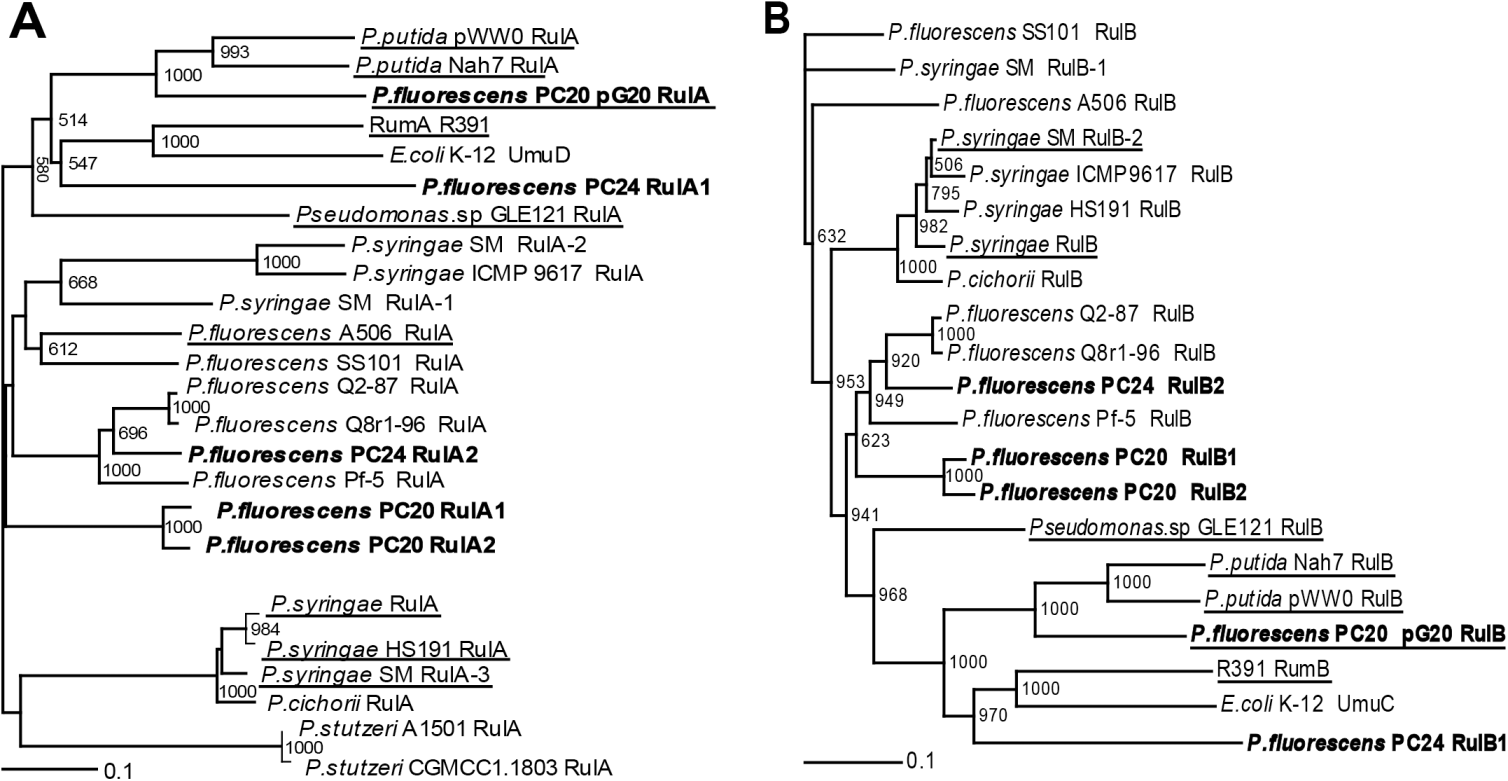
**The phylogenetic trees of RulA (A) and RulB (B) protein sequences. Underlining indicates the genes of proteins which are located on plasmids.** Sequences derived from *P*. *fluorescens* strains PC20 and PC24 are shown in boldface. Sequences were aligned with ClustalX2 and further visualised with TreeViewX. Numbers at branch nodes indicate bootstrapping values for 1000 bootstrap replicates. Values under 500 were removed. The identifiers of the aligned sequences are presented in S7 Table.

Further characterization of the putative Pol V genes from PC20 and PC24 revealed that the *rulAB* genes, which are located on the plasmid pG20 of the strain PC20, were clustered together with the *rulAB* sequences present on the catabolic plasmids pWW0 and NAH7 (Fig 3 and S7 Table). The two chromosomal copies, the *rulAB1* and *rulAB2* genes of the strain PC20 also clustered together, but were distantly related to the *rulAB*_*pG20*_. Meanwhile, the constructed phylogenetic tree revealed that the two copies of the *rulAB* genes identified in the chromosome of the strain PC24 were less related to each other. The *rulAB1*operon of PC24 was more closely clustered to the *umuDC* operon of *E*. *coli* K-12 and the *rulAB*_*pG20*_, but the *rulAB2* operon was more related to the *rulAB* copies identified in the strain PC20.

Multiple sequence alignments of the RulA and RulB homologs from the strains PC20 and PC24 with the sequence of the biochemically and structurally well characterized DNA polymerase Pol V from *E*. *coli* revealed that the sequences of the RulA and RulB from PC20 and PC24 contained all Pol V domains. Moreover, all of the analysed sequences with the only exception of the RulB sequence at the amino acid position 72 contained the same residues which have been shown to be essential for the catalytic activity of the *umuDC*-encoded Pol V in *E*. *coli* (S2 and S3 Figs, S7 Table).

**Fig 4:**
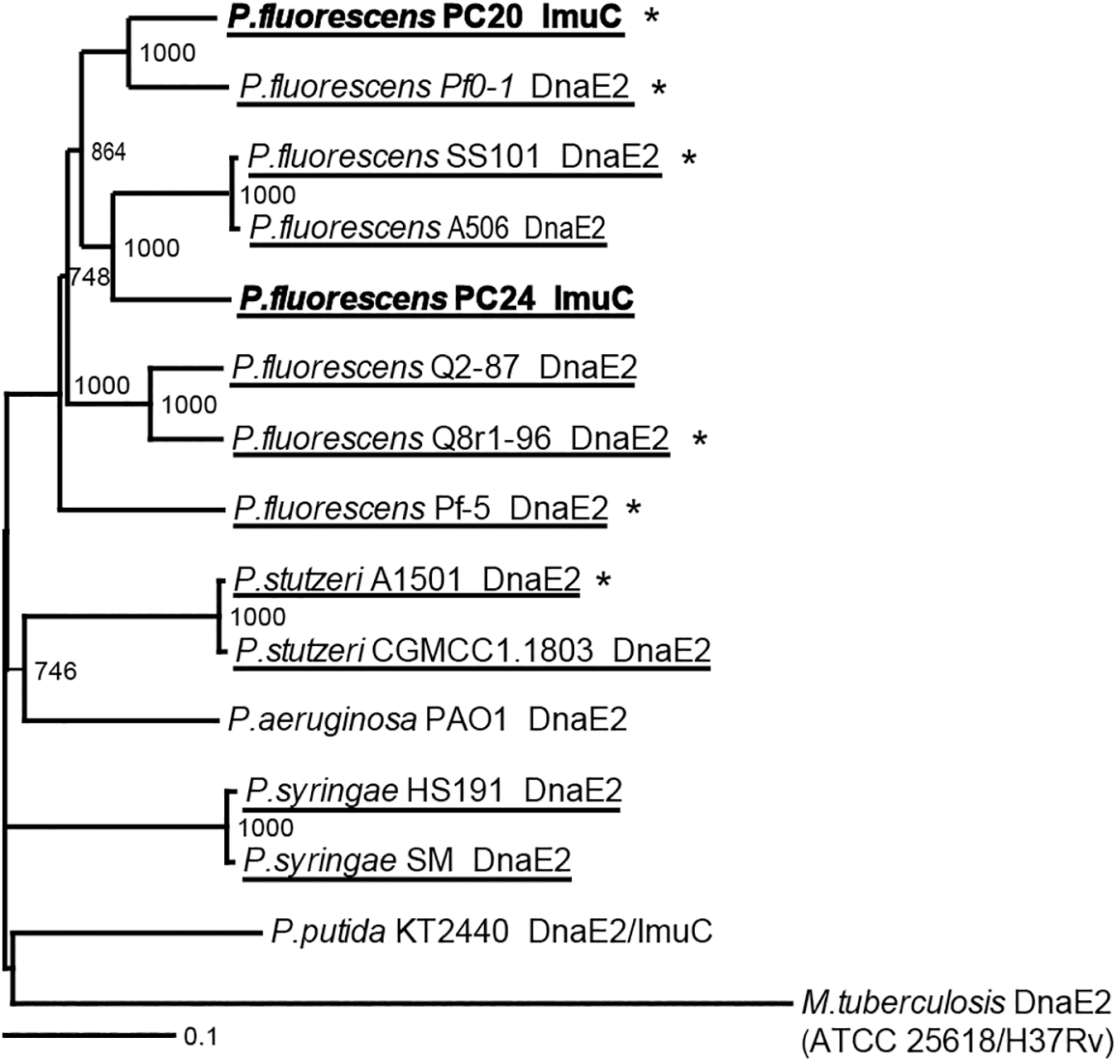
The phylogenetic tree of ImuC/DnaE2 protein sequences. The underlined proteins are from strains possessing *rulAB* genes and the ones marked with star are from strains where *rulAB* genes are located in the chromosome. Sequences derived from *P*. *fluorescens* strains PC20 and PC24 are shown in boldface. Sequences were aligned with ClustalX2 and further visualised with TreeViewX. Numbers at branch nodes indicate bootstrapping values for 1000 bootstrap replicates. Values under 500 were removed. The identifiers of the aligned sequences are presented in S7 Table.

Construction of the phylogenetic tree of the deduced amino acid sequences of the *imuC* from PC20 and PC24 and from other pseudomonads demonstrated that the ImuC homologs from PC20 and PC24 were more closely clustered with the homologs from *P*. *fluorescens* than with those from the other *Pseudomonas* species (Fig 4 and S7 Table). Based on multiple sequence alignment and according to the NCBI Conserved Protein Family database the ImuC homologs of PC20 and PC24 carry functional domains and putative active site residues of ImuC/DnaE2 polymerases (S4 Fig and S7 Table). Thus, the results of DNA sequence analysis indicated that most likely both PC20 and PC24 contain more than one copy of functional Pol V genes and a copy of *imuC*.

### The *rulB* and *imuC* homologs were induced under the conditions of DNA damage

**Fig 5:**
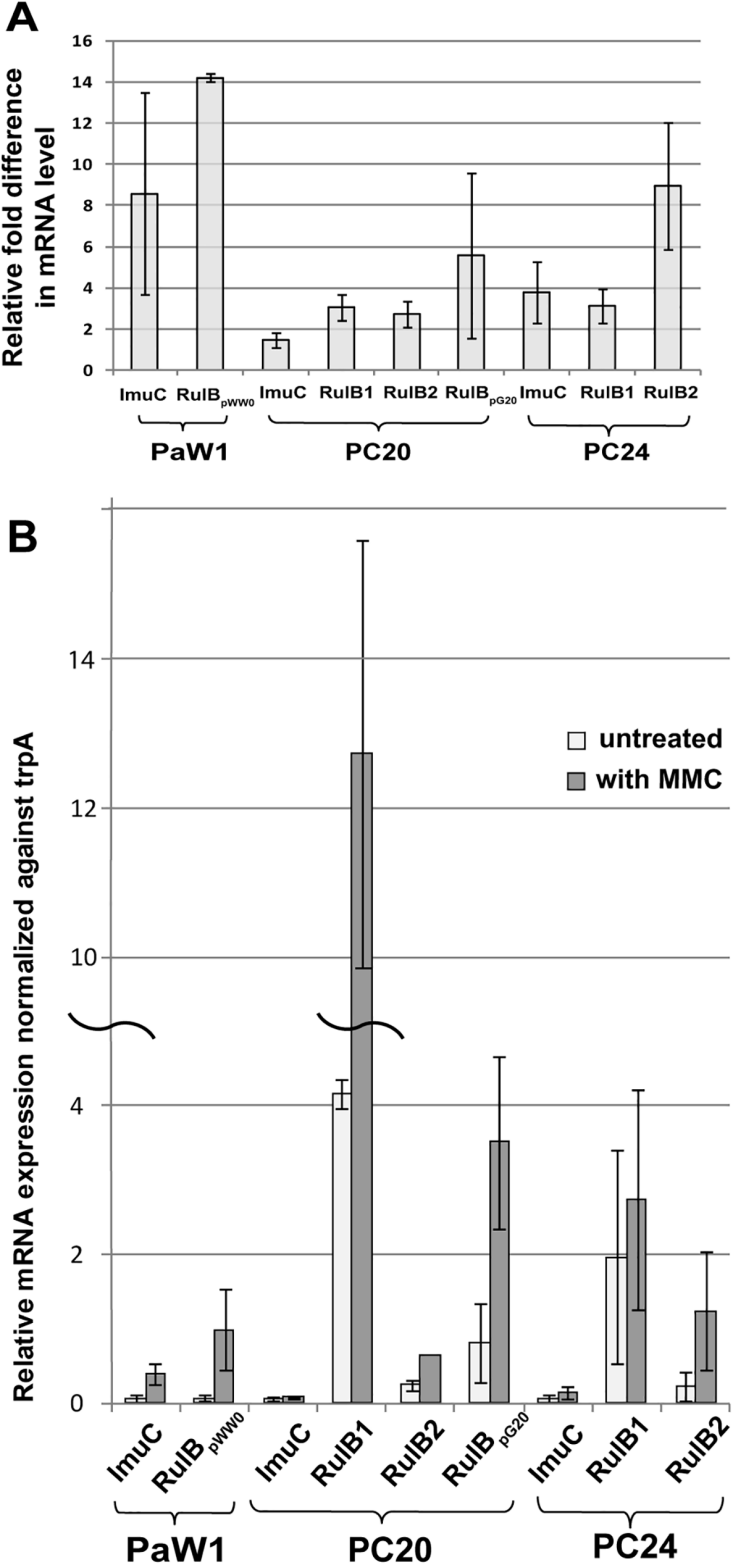
The expression rate of the genes of error prone DNA polymerases after the exposure of cells to DNA damaging agent MMC (2 μg/ml). (A) The results are presented as relative fold difference between cells with and without MMC treatment. (B) The expression rate of the studied error-prone DNA polymerases normalized against *trpA*. Each data represents the mean from at least three replicate induction experiments.

Although the strains PC20 and PC24 carried more than one copy of the *rulAB* genes in addition to the *imuC* gene, they were more sensitive to the UV irradiation than the strains PaW1 and PaWrulAB (Table 2). To further examine functionality of these putative DNA polymerase genes, we investigated whether all copies of the *rulAB* genes and the *imuC* gene were expressed under the conditions of exogenously induced DNA damage. We have previously shown that the transcription of the *rulAB* genes located on the plasmid pWW0 can be induced by the DNA damage-inducing chemical mitomycin C (MMC) [35]. Transcription from the LexA2-regulated promoter of the “mutagenesis cassette” encoding for ImuC polymerase in *P*. *putida* was also induced in the presence of MMC [43]. According to our previous studies the treatment of cells with MMC gives more reproducible results on gene expression profile than the exposure of bacteria to UV irradiation [35]. Therefore, in order to evaluate the expression of the putative DNA polymerase genes in the strains PC20 and PC24, a quantitative reverse transcriptase polymerase chain reaction (RT-qPCR) analysis was performed using RNA extracted from cells grown in the presence or absence of MMC (experimental details are given in Materials and Methods section). As a positive control we employed the *P*. *putida* strain PaW1 which carried the MMC-inducible *rulAB* operon on the TOL plasmid pWW0 and the *imuC* gene in the chromosome. The results shown in Fig 5 revealed that under such conditions the transcription of the *rulAB*_pWW0_ and the *imuC* gene were induced in the positive control strain PaW1 about 14 fold and 8 fold, respectively. The transcription of all copies of the *rulAB* operons as well the *imuC* gene in the strains PC20 and PC24 were also induced by MMC (Fig 5 and S8 Table). However, the induction levels of these genes were lower in comparison with the control strain (Fig 5A). At the same time, the basal level of transcription of the *rulAB* genes in the strains PC20 and PC24 was higher than that estimated in the reference strain PaW1 (Fig 5B).

**Fig 6:**
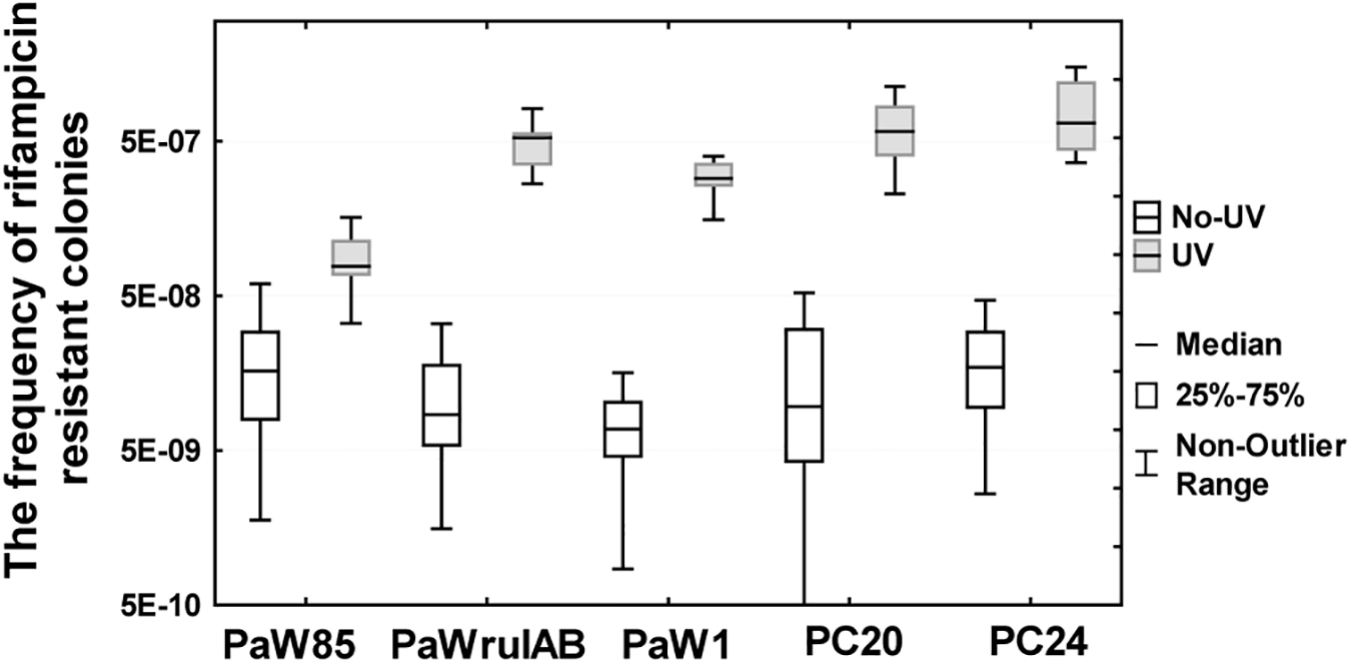
The comparison of spontaneous and UV-C irradiation (5 J/m^2^) induced Rif^r^ mutant frequencies. Each data represents the median value from three replicate UV-mutagenesis assays. Data were analysed using the Mann-Whiteny U test (S9 Table).

These results altogether suggested that the genes encoding for putative error-prone DNA polymerases Pol V and ImuC in the strains PC20 and PC24 could be functional and inducible under the conditions of DNA damage. Consequently, it was hard to understand why the UV mutagenesis phenotype, although quite modest, appeared only in the case of the strain PC24 (S6 Table). Meanwhile, examination of UV mutagenesis phenotype of the strain PC20 was complicated because the survival of this strain upon exposure to the UV-C irradiation at a dose of 100 J/m^2^ was very low. Thus, it is possible that the experimental conditions which were set up based on the commonly used protocols for the detection of UV mutagenesis are not optimal for this strain. Indeed, when bacteria were exposed to a UV-C irradiation dose of 5 J/m^2^ (this dose was about 20-fold lower than the commonly used dose), the strong UV mutagenesis phenotype was detected also with the strain PC20 (Fig 6 and S9 Table). Under such conditions the Rif^r^ mutant frequency increased about 60 times. Interestingly, the irradiation of cells at UV-C irradiation at a dose of 5 J/m^2^ elevated remarkably the UV mutagenesis also in the case of the other three studied strains PC24, PaWrulAB and PaW1 (Fig 6 and S9 Table). For example, the strain PC24 which exhibited about 4.5-fold increase in the number of Rif^r^ mutants above spontaneously induced levels at a dose of 100 J/m^2^ had approximately 38-fold elevated mutant frequency when cells were exposed to the UV irradiation at a dose of 5 J/m^2^. Even the reference strain *P*. *putida* PaW85 exhibited a clearly visible UV mutagenesis phenotype at this dose (4.8-fold elevated mutant frequency above the spontaneous levels). Thus, without testing for a wider range of environmental conditions one may easily underestimate a potential of the studied strains for the induced mutagenesis.

### Is the degradation of aromatic compounds mutagenic for bacteria?

Exposure of bacteria to aromatic compounds induces production of reactive oxygen species (ROS) which may cause DNA damage. This in turn may cause elevated mutation frequency and speed up the evolution of novel metabolic properties. To examine possible mutagenic effects of phenolic compounds, we incubated bacteria in the presence of phenol and 3-methylphenol (*m*-cresol). Notably, the indigenous strains examined in the current study are able to degrade various aromatic compounds (Table 1). According to the previously published data the strain PC20 can grow on phenol, *p*-cresol, salicylate and naphthalene, and the strain PC24 can grow on phenol, *p*-cresol and *m*-toluate, whereas PC20 degrades *p*-cresol via *meta* pathway and PC24 via protocatechuate pathway [24, 44, 45]. Here it is important to mention that during the current studies we discovered that the strain PC20 cannot grow any more on minimal medium containing *p*-cresol as a carbon source (data not shown). Importantly, both PC20 and PC24 contained the mono-component phenol monooxygenase gene *pheA* probably acquired after the release of laboratory bacteria employed for removal of phenolic pollution in contaminated water [24, 46]. The latter inspired us to use the phenol-degrading *P*. *putida* strain PaWpheBA25 with the introduced *pheBA* operon as a control. *P*. *putida* strain PaW85 unable to degrade phenol was also included for the comparison. Bacteria were grown on glucose CAA media similarly to that when we measured the spontaneous mutation frequency, except phenol or *m*-cresol was also added.

**Fig 7:**
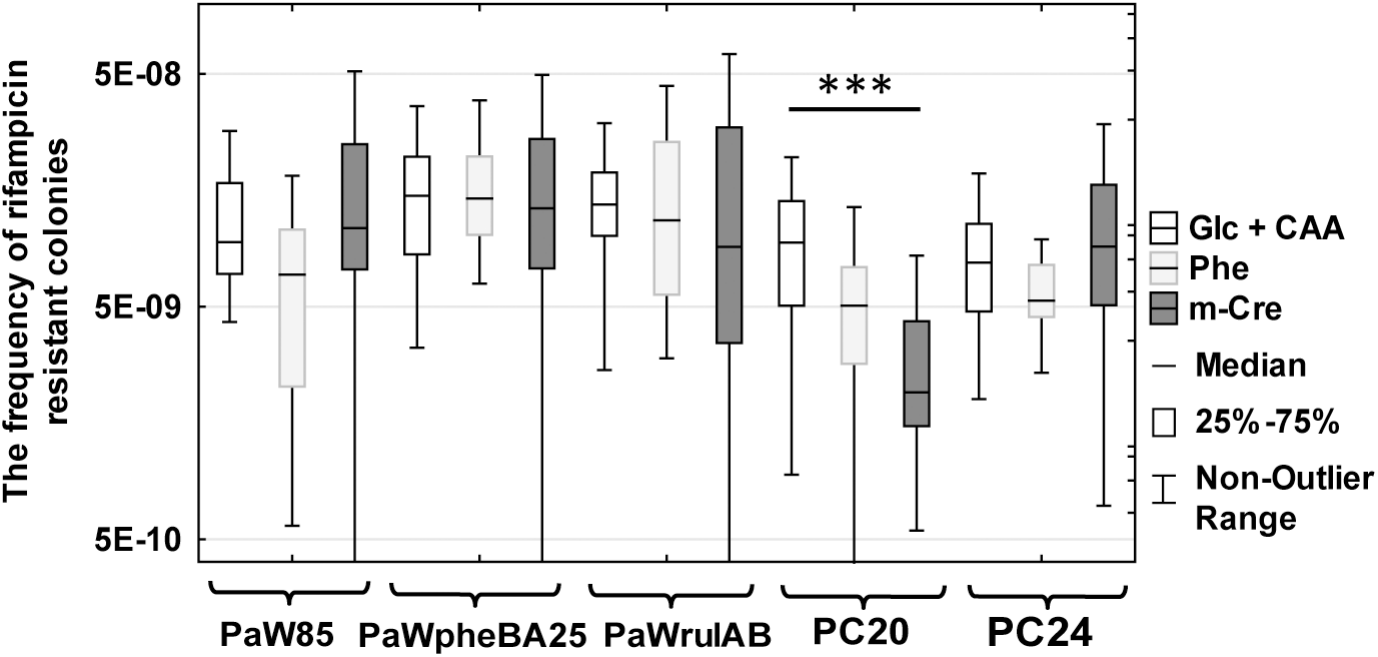
Study of the effect of phenolic compounds on the frequency of Rif^r^ mutants in nutritiously rich growth medium. Phenol or *m*-cresol was supplemented into M9 glucose medium supplemented with CAA for the strains PaW85, PaWpheBA, PaWrulAB and PC24 at the following concentrations: phenol (5 mM), *m*-cresol (2.5 mM). These compounds were added in lower concentrations (2.5 mM phenol and 1.25 mM *m*-cresol) for the strain PC20 because of growth inhibiting effects at higher concentrations. Data were analysed using the Kruskal-Wallis test (S10 Table). *** designates P<0.0001.

**Fig 8:**
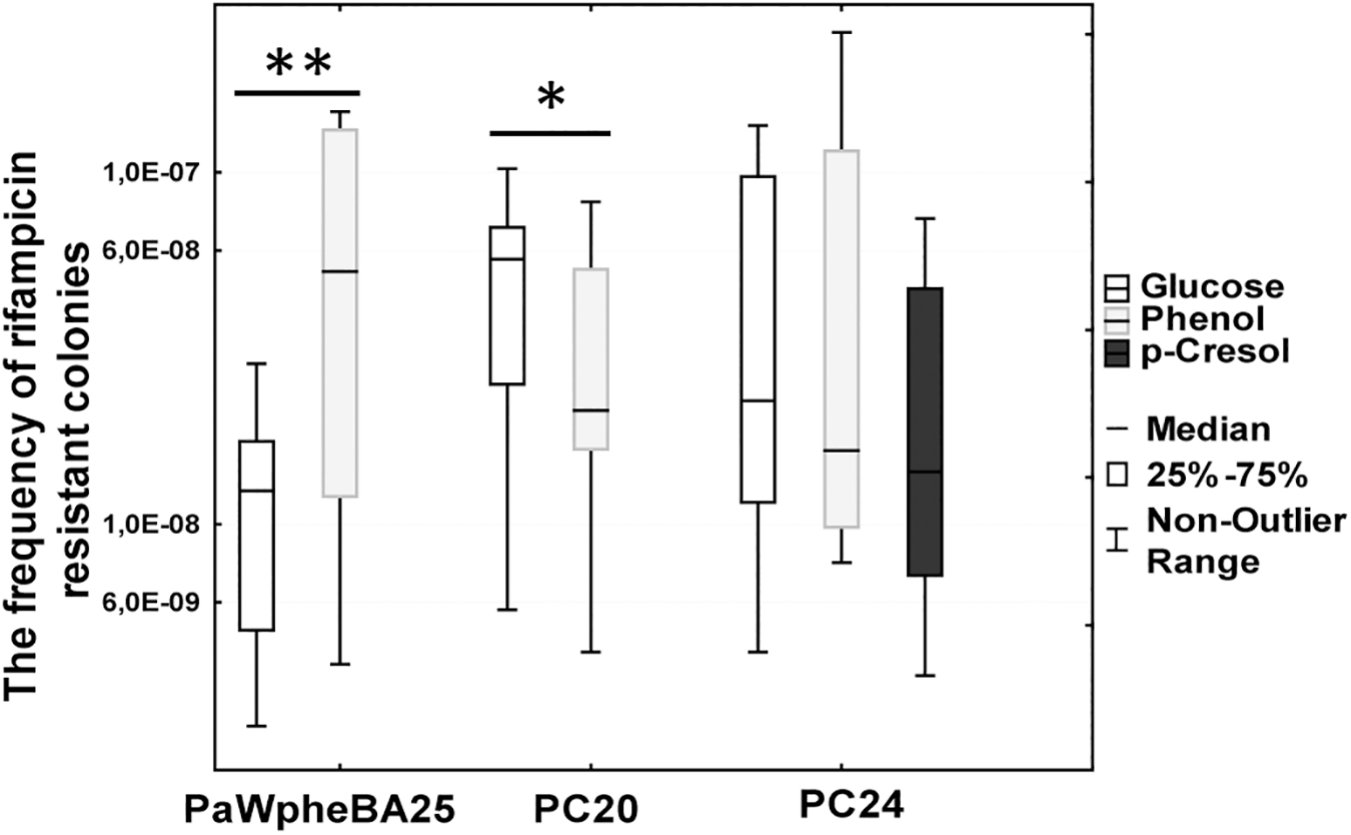
The frequency of rifampicin resistant colonies in M9 minimal medium which contained either glucose, phenol (2.5 mM) or *p*-cresol (1.25 mM) as the only carbon source. Data were analysed using the Kruskal-Wallis test (S11 Table). * designates P<0.025 and ** designates P<0.001.

To our surprise, all of the studied strains had similar or even reduced Rif^r^ mutant frequencies when phenol was present in the growth medium (Fig 7 and S10 Table). In the case of the strain PC20 the addition of *m*-cresol also lowered the Rif^r^ mutant frequency. Presented are only these results obtained with 5 mM phenol and with 2.5 mM *m*-cresol. Lower concentrations of these compounds had no effect on mutant frequency (data not shown). The results of this experiment implied that the studied bacteria have evolved highly functional mechanisms to cope with external or internal oxidative stress in the presence of phenolic compounds when the environment contains energetically rich growth substrates (glucose and amino acids). Therefore, in order to examine whether the availability of carbon and energy sources could affect potential mutagenic effects of the studied phenolic compounds, we repeated the experiment by cultivating bacteria in M9 minimal medium which either contained glucose, phenol or *p*-cresol as an only carbon source. No amino acids were supplied for the growth medium (for more details of the growth conditions of bacteria, see the Materials and Methods section). Indeed, it turned out that the growth of bacteria on minimal medium revealed the mutagenicity of phenol (Fig 8 and S11 Table). However, this appeared only under certain circumstances. Namely, the strain PaWpheBA25 (obtained by the introduction of the phenol degradation operon *pheBA* into the strain *P*. *putida* PaW85) which was constructed in the laboratory immediately before its use in the mutagenesis assay exhibited on phenol minimal medium about 4.2-fold elevated Rif^r^ mutant frequency in comparison to the growth on glucose minimal medium (Fig 8). At the same time, the indigenous isolate PC24 exhibited comparable frequency of Rif^r^ mutants on both growth substrates, and the strain PC20 exhibited even ~ 2.7 times reduced Rif^r^ mutant frequency when grown on phenol in comparison with the growth on glucose minimal medium. Thus, taken together, our results indicated that the mutagenic effect of aromatic pollutants depends on the availability of other carbon sources in the environment. These results also suggested that mutagenicity could be affected by a factor of how long bacteria have evolved to use a particular pollutant as a carbon source. Further studies are needed to test this hypothesis.

## Discussion

The frequency of mutations is usually held at low level. However, although most mutations are deleterious, bacteria with elevated spontaneous mutation frequency have been isolated from populations of human pathogens [16, 17, 47]. One of the strongest mutator phenotypes is caused by the inactivation of DNA mismatch repair (MMR) gene *mutS* which leads up to 100-fold higher mutation frequency [10, 48]. Populations can be enriched with mutators via several population bottlenecks occurring after strong selective challenges. For example, *P*. *aeruginosa* populations chronically infecting the lungs of CF-patients have to adapt to the highly compartmentalized and anatomically deteriorating lung environment, as well as to the challenges of the immune defence and antibiotic therapy [16, 18].

Is it possible that the evolution of bacteria with the capacity to degrade toxic environmental pollutants is also accompanied with the appearance of constitutive mutators? It is known that above a certain threshold organic pollutants affect survival of bacteria by altering the cell membrane structure or by impairing biosynthetic pathways essential for bacterial growth [20, 21, 49]. In addition, incomplete degradation of such compounds by bacteria lacking entire functional pathways for their catabolism may lead to the formation of toxic dead-end metabolites. Thus, bacteria which are evolving to degrade pollutants might be faced with strong selective pressure to survive in the presence of these toxicants. In the current study we assessed the existence of constitutive mutators within 53 indigenous isolates, mostly pseudomonads, which can degrade various toxic pollutants (e.g., phenol, toluene, and their methyl derivatives; alkanes) as a carbon source. Notably, the strains examined for a mutator phenotype in the current study included a number of pseudomonads which were isolated six years after an accidental fire in the oil shale mine in Estonia in 1988 [24, 46]. To reduce the produced pollution *in situ*, phenol-degrading *P*. *putida* laboratory strains carrying the phenol degradation operon *pheBA* on plasmid were deliberately released in the contaminated water. Several indigenous isolates examined in the current study have acquired this operon by the horizontal gene transfer [24, 46]. Thus, at least some of the investigated strains carry a signature that they have evolved under the conditions of heavily polluted environments. However, we did not find any strong mutators among the examined bacterial strains. Only two of the investigated strains expressed a moderate mutator phenotype (Fig 1). These results implied that constitutively increased mutability has not been prevalent in the evolution of bacteria exposed to toxic environmental pollutants. However, it should be noted that constitutive mutators might arise and persist only on a limited basis [50, 51]. *In vivo* experiments have shown a potential benefit of hypermutation for bacterial adaptation to new environments, but once adapted, this advantage disappears and the transmissibility of the mutator strains is considerably reduced [52]. When passed through severe bottlenecks, mutator lineages also accumulate mutations that confer loss in fitness [52, 53]. We suppose that the growth of pathogens in a body of its host might be much more limited than the growth of environmental bacteria living in a soil or water reservoirs. Once adapted to consume a toxic pollutant, environmental bacteria could have passed many more generations in open environment than pathogens in their host. Therefore, we cannot exclude a possibility that some of the isolated indigenous strains have lost their mutator phenotype. Drawing parallels from the literature, the results of the *mutS* gene sequence analysis from natural *E*. *coli* isolates have indicated that MMR functions have been repeatedly lost and reacquired during the evolutionary history of *E*. *coli* [54, 55]. Also, the experimental evidence of evolution towards reduced mutation rates in a Δ*mutS* mutator *E*. *coli* population was recently demonstrated [56].

About 11% of the investigated indigenous strains were hypomutators (Fig 1). It should be noted that hypomutable isolates have also been identified in several other bacterial populations, e.g., among clinical and environmental isolates of *P*. *aeruginosa*, *E*. *coli*, and *Stenotrophomonas maltophilia* [57–60]. The presence of hypomutators (or antimutators) along with isolates exhibiting increased mutation frequency indicates that mutator and antimutator alleles can often arise and spread in natural bacterial populations. Evolutionary forces affecting the genomic mutation rate by natural selection and random genetic drift have been extensively discussed, e.g., in [61–64].

The frequency of mutations can also be temporally induced. For example, mistranslation, and accumulation of oxidative and alkylation damage may transiently increase mutagenesis [10, 65, 66]. In addition, under stressful conditions such as exposure to antibiotics or starvation, DNA synthesis may become more erroneous because of the induction of error-prone DNA polymerases, resulting in a situation in which DNA repair systems are unable to cope with increasing amounts of replication errors [67–69]. Several bacterial DNA polymerases are induced as a part of SOS regulon in response to DNA damage. These DNA polymerases perform translesion DNA synthesis (TLS) when replication forks have collapsed at a blocking lesion whereas the DNA synthesis may be error-prone resulting in increased frequency of mutations in bacteria [11, 12, 70]. The results of our work revealed that a large proportion of the studied indigenous strains exhibit UV-irradiation-induced mutagenesis (Fig 2 and S6 Table). In many bacteria the UV-mutagenesis occurs as a consequence of TLS either performed by DNA polymerase Pol V or ImuC (former DnaE2) [12]. Error-prone DNA polymerase Pol V homologues are frequently encoded by naturally occurring catabolic conjugative plasmids and may facilitate generation of metabolic diversity in bacteria [35, 71, 72]. A closer inspection of two indigenous strains *P*. *fluorescens* PC20 and PC24 revealed that both strains harbour genes for ImuC and more than one copies of genes for Pol V.

The transcription of the genes encoding for Pol V and ImuC was induced under the conditions of DNA damage (Fig 5A). However, the level of the induction of the transcription of the *rulAB* genes by MMC was lower in the strains PC20 and PC24 than that of the *rulAB*_*pWW0*_ in the positive control strain PaW1 (Fig 5A). At the same time, the *rulAB* operons of PC20 and PC24 were expressed at higher basal level in comparison with the *rulAB*_*pWW0*_ operon (Fig 5B). The transcription of the DNA damage-inducible genes is negatively regulated by the LexA repressor protein which in *E*. *coli* binds to the conserved sequences CTGT-N_8_-ACAG [73]. Functional *E*. *coli* LexA homologues have been found in many other bacterial species as well [reviewed e.g., in [74]]. For instance, we have previously shown that the promoter region of the *rulAB* genes on pWW0 contains a DNA sequence showing a perfect match to the *E*. *coli* LexA-binding consensus sequence [35]. Analysis of the putative promoter sequences of the *rulAB* genes in the strains PC20 and PC24 also revealed the presence of a sequence which matched completely to the *E*. *coli* LexA repressor-binding consensus (S5 Fig). However, although there is a minimal LexA-binding consensus sequence, the timing and the level of induction can vary for each LexA-regulated gene because of multiple factors. The nucleotides surrounding the LexA consensus can influence the LexA binding affinity, and the strength of the promoter and the location of the LexA boxes relative to the promoter could also have an impact on the induction level [75, 76]. In addition, it should be noted that although the binding sites for the *E*. *coli*-like LexA repressor are similar for *E*. *coli* and *Pseudomonas* species, there might be some differences in the binding affinity. This assumption is supported by our previously published results demonstrating that the promoters of the *rulAB*_*pWW0*_ and the promoter of the DNA polymerase Pol IV gene *dinB* of *P*. *putida* carrying the *E*. *coli*-like LexA box are induced at higher level in *E*. *coli* than in *P*. *putida* [35, 77]. Bacteria living in soil and water reservoirs are constantly confronted with stressful conditions such as starvation for nutrients, desiccation, and non-optimal pH and temperature, all of which can induce DNA damage. Thus, one may speculate that soil bacteria, e.g., pseudomonads, have evolved a regulatory system enabling higher basal level of transcription of error-prone DNA polymerase genes because they have adapted to live in harsh and fluctuating environmental conditions inducing frequent injury in DNA.

Analysis of the putative promoter regions of the *imuC*-containing operon from PC20 and PC24 revealed the presence of the LexA2 binding sequences GTAC-N_4_-GTRC upstream of the structural genes (S6 Fig). Treatment of bacteria by DNA damaging agent MMC induced transcription of the *imuC* (Fig 5) which is in good accordance with the *imuC* expression profile in the other bacterial species [41–43]. Interestingly, although the strains PC20 and PC24 possessed several copies of DNA damage-induced DNA polymerase genes for Pol V and ImuC, according to the results of the measurement of UV tolerance both strains classified to a group exhibiting reduced tolerance to the UV irradiation in comparison with the strains PaWrulAB and PaW1 (Table 2 and S5 Table). The strain PC24 was even more sensitive to the UV irradiation than the *rulAB*-deficient reference strain PaW85 (Table 2). Besides TLS, the tolerance against UV irradiation can be affected by several other mechanisms. The main mechanism to tolerate UV-damage is nucleotide excision repair pathway (NER) which repairs the damage caused by UV irradiation in non-mutagenic way [78, 79]. However, analysis of the draft genome sequence of PC20 and PC24 revealed the presence of all NER pathway genes *uvrA*, *uvrB*, *uvrC* and *uvrD* (S7 Fig).

Here we wish to note that the role of ImuC/DnaE2 as an error-prone TLS polymerase protecting cells against DNA damage may not be general. For example, *Streptomyces coelicolor dnaE2* was found to be SOS-inducible, but it was not involved in UV tolerance or mutagenesis [80]. Similarly, we have previously reported that the deletion of the *dnaE2* (*imuC*) gene from the chromosome of *P*. *putida* PaW85 did not affect cells’ tolerance to the UV irradiation [81]. At the same time, the role of *P*. *putida* ImuC as a mutagenic DNA polymerase seems to be controversial. We have observed that the absence of this DNA polymerase increases the frequency of accumulation of mutations in carbon-starved *P*. *putida* [82]. On the other hand, the presence of ImuC altered the spectrum of mutations in *P*. *putida* only in the case when bacteria were exposed to UV-C irradiation (no differences could be detected without treatment of cells by DNA damaging agents), suggesting its participation in DNA replication under the conditions of DNA damage [81]. Moreover, our recent data [83] have indicated that mutagenesis induced by DNA alkylating agent MMS is largely ImuC-dependent both in *P*. *putida* and *P*. *aeruginosa*. Thus, participation of this DNA polymerase in mutagenic pathways could vary depending on the environmental conditions of bacteria.

Mechanisms reducing mutation rates (e.g., proofreading during DNA replication, expression of DNA repair functions, export or degradation of mutagenic compounds) could be energetically costly for cells. Therefore, it is reasonable to assume that rich growth environment allows more resources for establishment of DNA integrity. If the amount of nutrients becomes limiting, bacteria could start saving energy for other cellular functions by shutting down replication fidelity mechanisms. For instance, there is evidence that starvation is accompanied by increased frequency of mutations [67, 68, 84, 85]. The results of the current study implied that the availability of nutrients may affect frequency of mutations in bacteria (compare the results in Figs 7 and 8). Specifically, when we examined the effect of pollutants (e.g., phenol) on the induction of Rif^r^ mutations, we did not observe any increasing effects of phenol on the mutation frequency if bacteria were grown in the environment supplemented by glucose and amino acids. The mutation frequency was increased only in minimal medium when phenol was served as an only carbon and energy source (Fig 8).

Nevertheless, the mutagenic effect of phenol appeared only with the strain PaWpheBA which was constructed by introducing the phenol degradation genes *pheBA* into the reference strain *P*. *putida* PaW85. On the contrary, the mutation frequency of the indigenous strains PC20 and PC24 was declined on phenol minimal medium. As already mentioned above, the strains PC20 and PC24 have most likely acquired the *pheBA* operon from laboratory strains released into contaminated water after fire accident in 1988 [46]. Indigenous bacteria present in the area of phenolic contamination possibly met high concentration of the pollutants both before and after the acquisition of the *pheBA* operon. Bacterial strains obtained the *pheBA* operon were isolated 6 years later, which have allowed mutual adaptation between the bacterial host and the acquired pathway genes by thousands of generations. Phenol like other environmental pollutants act both as nutrients and as a stressors for bacteria [20]. Therefore, efficient phenol degraders should evolve also efficient mechanisms to deal with phenolic stress. Hence, one may speculate that while degrading phenolic compounds, the indigenous strains PC20 and PC24 have developed stress response mechanisms which can reduce mutational load when exposed to these pollutants. Further studies are in progress to test this idea.

### Concluding remarks

The evolution of environmental pollutants degrading bacteria is an ongoing process. Both horizontal transfer of genes and mutagenesis induced under stressful conditions might facilitate development of new catabolic pathways. It has been suggested that growth of bacteria in the presence of pollutants is also mutagenic. The indigenous bacterial isolates characterized in the current study, many of them collected from polluted environments, did not display a strong constitutive mutator phenotype. Nevertheless, a large proportion of the investigated strains exhibited a potential for induced mutagenesis under the conditions of DNA damage. Two phenol and cresol degrading *P*. *fluorescens* strains selected for a deeper characterization possessed several copies of DNA damage-induced error-prone DNA polymerases Pol V and ImuC. However, it is possible that compared to the laboratory-constructed strains these indigenous bacteria have already evolved an efficient mechanism for degradation of these phenolic compounds without increasing mutation frequency. Further elucidation of mechanisms of stress response in these indigenous strains would be useful to understand processes of efficient bioremediation. Also, laboratory experimental evolution experiments with bacteria which have acquired novel catabolic genes for the degradation of pollutants only lately would shed light in the evolution of new metabolic properties of bacteria.

## Materials and methods

### Bacterial strains and media

The bacterial strains used in this study are described in Table 1. All indigenous strains listed in Table 1 are deposited in the Collection of Environmental and Laboratory Microbial Strains (CELMS; available in the Estonian Electronic Microbial dataBase (EEMB) website http://eemb.ut.ee). Complete medium used was lysogeny broth (LB) medium [86] or R2A [87], and minimal medium was M9 (Adams, 1959). Solid medium contained 1.5% Difco agar. Casamino acids (CAA) and glucose were added to the minimal medium at final concentrations of 0.4% and 0.2%, respectively. Aromatic compounds were added to the media in following concentrations: phenol (1.25 mM, 2.5 mM or 5mM); *m*-cresol (1.25 mM, 2.5 mM) and *p*-cresol (1.25 mM). Rif-LB plates contained 100 mg/ml of rifampicin (Rif) and Sm-LB plates contained 200μg/ml of streptomycin (Sm). All strains were incubated at 30°C and liquid cultures were grown on rotary shakers (180 rpm).

### Estimation of spontaneous mutation frequency by fluctuation test

In order to estimate spontaneous mutation frequency, we performed the fluctuation tests and calculated the median value for mutants per 1 x 10^9^ cells as described in [88]. The frequency of Rif^r^ mutants was determined at least in 13 independent cultures for each studied bacterial strain. Independent cultures were generated by growing cells to late logarithmic growth phase in M9 medium which contained glucose (0.2%) and CAA (0.4%). The cultures were then diluted by 10^5^ into fresh glucose and CAA-containing M9 medium. After that 2.3-ml aliquots were dispensed into test tubes and cells were allowed to grow 24 h to reach saturation. Approximately 2 × 10^9^ cells were plated onto Rif-LB plates. Emergence of Rif^r^ colonies were counted after 72 h of incubation. The same protocol was also used to study the frequency of streptomycin resistant mutants (Sm^r^) with an exception of plating cells to Sm-LB plates (Sm 200 μg/ml). To exclude possibility that the estimated mutant frequencies of the studied environmental isolates were affected by variations in the tolerance to Rif or Sm, we measured the minimal inhibitory concentrations (MIC) of used antibiotics on a selection of strains (10 strains). For MIC assay the cells were grown overnight in glucose and CAA-containing M9 medium and then diluted 100 fold into fresh medium containing serial dilutions of antibiotics. Cells were incubated in microtiter plates at 30°C for 24h and then culture density was measured at OD580. MIC was defined as the lowest concentration of antibiotic which prohibit bacterial growth.

### Estimation of the effect of aromatic compounds on the frequency of Rif^r^ mutants

The influence of aromatic compounds in the growth medium was studied in high and low nutrient M9 medium. In the case of high nutrient medium independent cultures were generated by growing cells overnight in M9 medium supplemented with glucose and CAA. The cultures were then diluted by 10^5^ into fresh glucose and CAA containing M9 medium without adding any other compounds or supplemented with either 2.5 mM or 5 mM phenol, or 1.25 mM or 2.5 mM m-cresol. Next 2.3 ml aliquots were dispensed into flasks and cells were allowed to grow 48 h to reach saturation. Approximately 2 × 10^9^ cells were plated onto Rif-LB plates. Emergence of Rif^r^ colonies was counted after 72 h of incubation. In the case of low nutrient medium independent cultures were generated by growing cells overnight in M9 medium supplemented with glucose. The cultures were then diluted by 100 fold into fresh M9 minimal medium containing either glucose, phenol (2.5 mM) or *p*-cresol (1.25 mM) as the only carbon source. Next 15 ml aliquots were dispensed into flasks and cells were allowed to grow 48 h to reach saturation. Approximately 2 × 10^9^ cells were plated onto Rif-LB plates. The emergence of Rif^r^ colonies were counted after 72 h of incubation. The median value for Rif^r^ mutants was calculated as described above.

### UV-irradiation survival studies

The studied strains were grown overnight in glucose and CAA containing M9 media, and the serial of dilutions, 10^−4^ to 10^−9^ from the initial overnight culture, were placed as 10 μl drops on the LB agarose plates. The plates with dilutions were then subjected to UV-C irradiation which was performed at a 254-nm wavelength at a dosage of 10, 20, or 40 J/m^2^ using a CX—2000 Crosslinker. Irradiated plates were incubated in the dark for 24 h before the enumeration of CFU. Survival was expressed as the number of CFU detected after irradiation as a percentage of those detected after no treatment. At least 3 independent experiments were performed for each strain.

### UV-mutagenesis assays

To ensure the exponential growth of bacteria, the cultures grown overnight in glucose and CAA containing M9 medium were diluted 1:100 into fresh medium. After the cultures reached mid-exponential phase (optical density at 580 nm of 0.6 to 0.8), 11 ml of each culture was transferred to a 100-mm-diameter sterile Petri dish for UV-irradiation. The irradiation with UV-C light was performed at a 254-nm wavelength at a dose of 100 J/m^2^ (if not stated otherwise in the Results section) using a CX—2000 Crosslinker. Aliquots (1.6 ml) were removed from the Petri dish and grown in test tubes overnight with shaking at 180 rpm. 500 μl of overnight culture was plated onto rifampicin plates and the appearance of mutants was analysed as described before. However, if the amount of Rif^r^ colonies was uncountable on the plates, the assay was repeated and only 100 μl of overnight culture was plated.

### Nucleotide sequencing and *in silico* analysis

The total genomic DNA from the bacterial strains PC20 and PC24 was isolated and the whole genome sequencing was performed as described in [89] (S1 Appendix). In case of PC20, the total length of the assembled contigs was 7,856,954 bp (230-fold sequencing coverage), in 3749 contigs. The contigs N50 was 52,853 bp with the longest scaffold 204,810 bp. In case of PC24, the total length of the assembled contigs was 6,457,994 bp (251-fold sequencing coverage), in 878 contigs. The contigs N50 was 101,312 bp with the longest scaffold 259,869 bp. Gaps in the contigs used in this study were sequenced with 3730*xl* DNA Analyzer (Applied Biosystems) using the BigDye® Terminator v3.1 Cycle Sequencing Kit (Applied Biosystems) and protocols provided by the manufacturer.

The contigs of PC20 and PC24 containing the genes involved in DNA replication and repair were detected from all obtained contigs using the program BLASTP version 2.2.31 with default parameters except for the E value cutoff which was 10^−3^. The database file for searches was created from sequences obtained from NCBI’s Protein Clusters database (1976 sequences of different proteins involved in DNA replication and repair). The ORFs of the selected contigs were subsequently predicted using the program Prodigal version 2.6.1 [90]. Sequences were aligned and phylogenetic trees were constructed with ClustalX version 2.1 [91] and further visualised with TreeViewX version 0.5.0 [92].

The nucleotide sequences analysed in the current study are deposited in GenBank (see S7 Table for GenBank accession numbers).

### RNA extraction and RT-qPCR

In order to study the expression rate of error-prone DNA polymerases, the cultures were grown overnight in glucose and CAA containing M9 medium and then diluted 1:100 into fresh medium. After two hours of growth, the expression of DNA damage tolerance mechanisms was induced for 2 h with the addition of MMC mitomycin C (MMC) with the final concentration of 2μg/ml. Next the RNA was extracted using RNAzol^®^RT kit (Molecular Research Centre, Inc) with manufacturer’s instructions from normal and MMC induced cells. Additional treatment with DNase I (Fermentas) was performed according to the protocol (Fermentas) after which the quantity of RNA was measured with spectrophotometer (The NanoDrop™ 1000, Thermo Scientific). The quality of RNA was first inspected by electrophoresis (1% agarose) for the integrity and secondly with PCR for contamination with DNA.

The quantitative reverse transcription-PCR (qRT-PCR) assay was performed with SuperScript® III One-Step RT-PCR System with Platinum® *Taq* DNA Polymerase (Invitrogen) in the total volume of 10 μl. The RT-qPCR was operated in the real-time PCR system Rotor-Gene® Q (Qiagen). In RT-qPCR the primers were used at concentration of 0.4 μM and their sequences are presented in S12 Table. The primers were designed to generate products of ~200 bp. The reaction conditions were: 50°C for 3 min, 95°C for 5 min, followed by 40 cycles of denaturation at 95°C for 15 s, annealing at 58°C for 30 s, and extension at 72°C for 20 s. The fluorescence intensity of SYBR Green was measured automatically at the end of the extension step. At the end of run, melting curve analyses was performed increasing the temperature from 72°C to 95°C, using 4 s and 1°C interval with continuous fluorescence recording. Raw data from the RT-qPCR was analyzed with Rotor-Gene Series software, version 2.0.2.4 (Qiagen) and mRNA quantities were calculated using the LinRegPCR software version 2015.3 [93]. Data from at least three separate RT-qPCR experiments were averaged and normalized against the *trpA* levels, which expression is not affected by DNA damage [75].

### Statistical analysis of the results

Sharipo-Wilk test for normality was used for all distributions and because normality was not addressed, the nonparametric Mann-Whitney U test or Kruskal-Wallis test was used for comparisons. The calculations were performed using Statistica 10 software [StatSoft, Inc. (2011). STATISTICA (data analysis software system), version 10. www.statsoft.com.]. For statistical tests the significance level was set at P = 0.05. In order to reduce the amount of potential errors on multiple comparisons, Benjamini-Hochberg procedure was used with false discovery rate (FDR) of 0.05 [94].

Original datasets used to compute statistics are available as a part of supporting information (S13 Table).

## Supporting information

S1 Appendix Experimental procedure for whole genome sequencing. (DOCX)

S1 Fig Comparison of Sm^r^ and Rif^r^ resistant mutant frequencies in representatives of the indigenous strains and the reference strain *P*. *putida* PaW85. The median value of the frequency of Sm^r^ mutants of the strains C70 and P4 was 0 (the mean values were 1.65 x 10^−9^ and 4.8 x 10^−10^, respectively).
(PDF)

S2 Fig Multiple sequence alignment of RulA homologs from *Pseudomonas fluorescens* PC20, PC24; *Pseudomonas putida* plasmid pWW0 RulA (Q8VMP5) and *E*. *coli* K-12 UmuD (P0AG11). Black boxes indicate the putative active sites residues according to NCBI Conserved Protein Domain Family database (source pfam; superfamily cl10465; Peptidase_S24_S26; [95]. Residues shaded orange represent the autocatalytic cleavage site shown to be required for posttranslational processing of UmuD/RulA homologs [96, 97]. Residues surrounded by pink boxes have been shown to be important in the UmuDC mediated mutagenesis [98]. Sequences were aligned with ClustalX2 and domains were marked according to Protein sequence analysis and classification portal Interpro (http://www.ebi.ac.uk/interpro/) based on the sequence of UmuD (P0AG11).
(PDF)

S3 Fig Multiple sequence alignment of RulB homologs from *Pseudomonas fluorescens* PC20, PC24; *Pseudomonas putida* plasmid pWW0 RulB (Q8VMP6) and *E*. *coli* K-12 UmuC (P04152). Black boxes indicate the putative active site residues according to NCBI Conserved Protein Domain Family database (source cd00424; superfamily cl12025; PolY_Pol_V_umuC; [95]. Residues surrounded by pink boxes have been shown to be important in the UmuDC mediated mutagenesis [98–106]. Sequences were aligned with ClustalX2 and domains were marked according to Protein sequence analysis and classification portal Interpro (http://www.ebi.ac.uk/interpro/) based on the sequence of UmuC (P04152).
(PDF)

S4 Fig Multiple sequence alignment of ImuC/DnaE2 homologs from *Pseudomonas fluorescens* PC20. **PC24, *Pseudomonas putida* KT2440 DnaE2/ImuC (Q88182), and *Mycobacterium tuberculosis* (A0A089QXJ1).** Black boxes indicate the putative active site residues according to NCBI Conserved Protein Domain Family database (source cd07431; superfamily cl23724; PHP_PolIIIA_DnaE2; [95]. Residues shaded gray represent active-site amino acids which are highly conserved among Y-family polymerases [107]. Residues underlined with red are conserved active site regions according to the study by [108]. Sequences were aligned with ClustalX2 and domains were marked according to Protein sequence analysis and classification portal Interpro (http://www.ebi.ac.uk/interpro/) based on the sequence of *P*. *putida* KT2440 DnaE2/ImuC (Q88182). Region underlined with black is a C-terminal amino acid motif -[S/T/G]R[D/N]F[D/R/H]- highly conserved in DnaE2-type proteins [107].
(PDF)

S5 Fig Multiple sequence alignment of putative promoter regions of *rulAB* genes and *E*. *coli umuDC* genes. The -35 and -10 hexamers of the promoters are marked by black boxes and transcriptional start site for *umuDC* promoter [109] is marked with red box. LexA-binding consensus sequence is aligned on the last row [110]. Sequences were aligned with ClustalX2.
(PDF)

S6 Fig Multiple sequence alignment of putative promoter regions of *imuABC*. The -35 and -10 hexamers of the promoters are marked by black boxes. LexA2-binding consensus sequence is aligned on the last row [43]. Sequences were aligned with ClustalX2.
(PDF)

S7 Fig **The phylogenetic trees of UvrA (A), UvrB (B), UvrC (C) and UvrD (D) protein sequences.** Sequences were aligned with ClustalX2 and further visualised with TreeViewX. Numbers at branch nodes indicate bootstrapping values for 1000 bootstrap replicates. Values under 500 were removed. The identifiers of the aligned sequences are presented in S7 Table.
(PDF)

S1 Table The median values of the frequency of spontaneous Rif^r^ mutants. Three laboratory strains were studied as a comparison in addition to the environmental strains. All of the mutant frequencies were compared to *P*. *putida* PaW85 laboratory reference strain with Kruskal-Wallis test. The statistically significant p-values according to Benjamini-Hochberg procedure are indicated with red (FRD = 0.05).
(DOCX)

S2 Table The minimal inhibitory concentrations of representatives of the indigenous strains and the reference strain *P*. *putida* PaW85. (DOCX)

S3 Table The results of Mann-Whitney U test for comparing the appearance frequency of mutants for two different antibiotics (rifampicin and streptomycin) within one strain. The statistically significant p-values according to Benjamini-Hochberg procedure are indicated with red (FRD = 0.05).
(DOCX)

S4 Table Comparison of Sm^r^ mutant frequencies with Kruskal-Wallis test against PaW85. The statistically significant p-values according to Benjamini-Hochberg procedure are indicated with red (FRD = 0.05).
(DOCX)

S5 Table The survival percentage of bacteria after UV-C irradiation. Underlined are the strains with at least 10 fold higher UV-tolerance (UV- 20 J/m^2^) compared to PaW85. Kruskal-Wallis test was performed to compare UV-tolerance (UV- 20 J/m^2^) of the studied strains against PaW1, which possesses PolV coding operon *rulAB*. The statistically significant p-values according to Benjamini-Hochberg procedure are indicated with red (FRD = 0.05).
(DOCX)

S6 Table The UV-induced (100 J/m^2^) Rif^r^ mutant frequencies. The Mann-Whitney U test was performed to distinguish statistically significant differences of UV-induced and spontaneous Rif^r^ mutant frequencies. The statistically significant p-values according to Benjamini-Hochberg procedure are indicated with red (FRD = 0.05). The difference between spontaneous and UV-induced mutant frequency is presented as fold of induction. Underlined are the strains with at least 10 fold higher tolerance against UV irradiation (UV-20 J/m^2^) compared to PaW85.
(DOCX)

S7 Table The sequences used in phylogenetic analyses and multiple sequence alignments. Protein sequences were translated based on the gene sequences listed below. Sequence ID-s were obtained from GenBank (https://www.ncbi.nlm.nih.gov/genbank/) and The European Nucleotide Archive (ENA, http://www.ebi.ac.uk/ena).
(DOCX)

S8 Table The statistical analysis for distinguishing significant changes in the expression rate of the genes of error-prone DNA polymerases after treatment with MMC. The RT-qPCR results were analyzed with Mann-Whitney U test and p-values indicated with red represent statistically significant differences according to Benjamini-Hochberg procedure (FRD = 0.05).
(DOCX)

S9 Table The comparison of spontaneous and UV-induced (5 J/m^2^) mutant frequencies. The Mann-Whitney U test was performed to distinguish statistically significant differences of UV-induced and spontaneous Rif^r^ mutant frequencies according to Benjamini-Hochberg procedure (FRD = 0.05). Red indicates the statistically significant differences. The difference between spontaneous and UV-induced mutant frequency is presented as a fold of induction.
(DOCX)

S10 Table The effect of aromatic compounds (2.5 mM *m*-cresol or 5 mM phenol with exception of PC20 for which we used 2.5 mM phenol and 1.25 mM *m*-cresol) to the appearance frequency of Rif^r^ mutants in rich medium (M9 medium supplemented with glucose (0.2%) and CAA (0.2%)). Multiple comparisons with Kruskal-Wallis test were performed to distinguish statistically significant differences in the appearance frequency of Rif^r^ mutants in the presence of aromatic compounds. Red indicates the statistically significant differences according to Benjamini-Hochberg procedure (FRD = 0.05). The effect of aromatic substrates to the frequency of Rif^r^ mutants is presented as a fold of induction compared to the cells grown on medium without aromatic compounds.
(DOCX)

S11 Table The frequency of Rif^r^ mutants grown in M9 minimal medium supplemented with either glucose (0.2%), phenol (2.5 mM) or *p*-cresol (1.25 mM) as a sole carbon and energy source. The Mann-Whitney U test was performed to distinguish statistically significant differences in the appearance frequency of Rif^r^ mutants in the presence of different carbon sources. Red indicates the statistically significant results according to Benjamini-Hochberg procedure (FRD = 0.05). The effect of aromatic substrates on the frequency of Rif^r^ mutants is presented as a fold of induction compared to the cells grown on glucose as a sole carbon source.
(DOCX)

S12 Table Primers used in the RT-qPCR experiment. (DOCX)

S13 Table Original datasets used to compute statistics. (DOCX)
