# Supplementary material for: Contribution of increased mutagenesis to the evolution of pollutants-degrading indigenous bacteria
Source: PLoS One. 2017 Aug 4;12(8):e0182484. doi: 10.1371/journal.pone.0182484 (PMC5544203; doi:10.1371/journal.pone.0182484)
Supplement: S1 Appendix — (DOCX) [file pone.0182484.s001.docx]

**Supporting information**

**S1 Appendix**

**Experimental procedure for whole genome sequencing**

The total genomic DNA from the bacterial strains PC20 and PC24 was isolated using UltraClean ^TM^ Microbial DNA Isolation Kit (MO BIO Laboratories, Inc.) according to the manufacturer’s instructions. The isolated DNA from the bacterial strains was fragmented, samples for whole genome sequencing were prepared using the Illumina’s TruSeq® DNA PCR-Free LT Sample Preparation Kit and sequenced by default paired-end read protocols (2 x 100 bp) using an Illumina HiSeq2500.

Whole genome assembly was performed using the Velvet version 1.2.09 software [[1](#_ENREF_1)]. In order to find the assembly with the best N50 value, different quality cutoff-values and different assembly program parameters were tested. Before assembly, reads with low quality at the 3’ end were trimmed using cutoff-value 20, 30 and 35 using program fastq_quality_trimmer from FASTX-Toolkit (http://hannonlab.cshl.edu/fastx_toolkit/). Trimmed reads shorter than 40 bp were discarded. Reads trimmed with quality cutoff-value 30 resulted in the best N50 value. Velvet K-mer values 31, 41 and 51 were tested. The best N50 values were achieved using the k-mer value of 31.

1. Zerbino DR, Birney E (2008) Velvet: algorithms for de novo short read assembly using de Bruijn graphs. Genome Res 18: 821-829.
