# Supplementary material for: Contribution of increased mutagenesis to the evolution of pollutants-degrading indigenous bacteria
Source: PLoS One. 2017 Aug 4;12(8):e0182484. doi: 10.1371/journal.pone.0182484 (PMC5544203; doi:10.1371/journal.pone.0182484)
Supplement: S1 Fig — The median value of the frequency of Smr mutants of the strains C70 and P4 was 0 (the mean values were 1.65 x 10^−9^ and 4.8 x 10^−10^, respectively). (PDF) [file pone.0182484.s002.pdf]

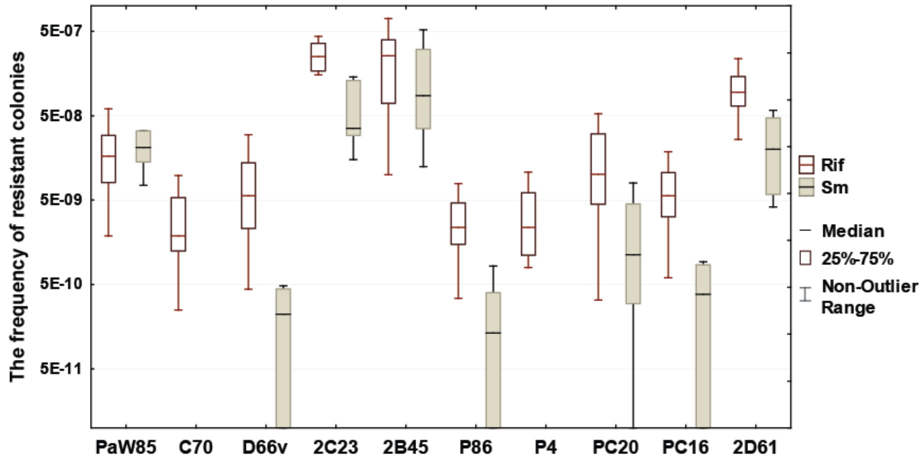

S1 Figure. Comparison of Sm^r and Rif^r resistant mutant frequencies in representatives of the indigenous strains and the reference strain *P. putida* PaW85. The median value of the frequency of Sm^r mutants of the strains C70 and P4 was 0 (the mean values were 1.65 x 10^{-9} and 4.8 x 10^{-10}, respectively).
