# Supplementary material for: Contribution of increased mutagenesis to the evolution of pollutants-degrading indigenous bacteria
Source: PLoS One. 2017 Aug 4;12(8):e0182484. doi: 10.1371/journal.pone.0182484 (PMC5544203; doi:10.1371/journal.pone.0182484)
Supplement: S3 Fig — Black boxes indicate the putative active site residues according to NCBI Conserved Protein Domain Family database (source cd00424; superfamily cl12025; PolY_Pol_V_umuC; [95]. Residues surrounded by pink boxes have been shown to be important in the UmuDC mediated mutagenesis [98–106]. Sequences were aligned with ClustalX2 and domains were marked according to Protein sequence analysis and classification portal Interpro (http://www.ebi.ac.uk/interpro/) based on the sequence of UmuC (P04152). (PDF) [file pone.0182484.s004.pdf]

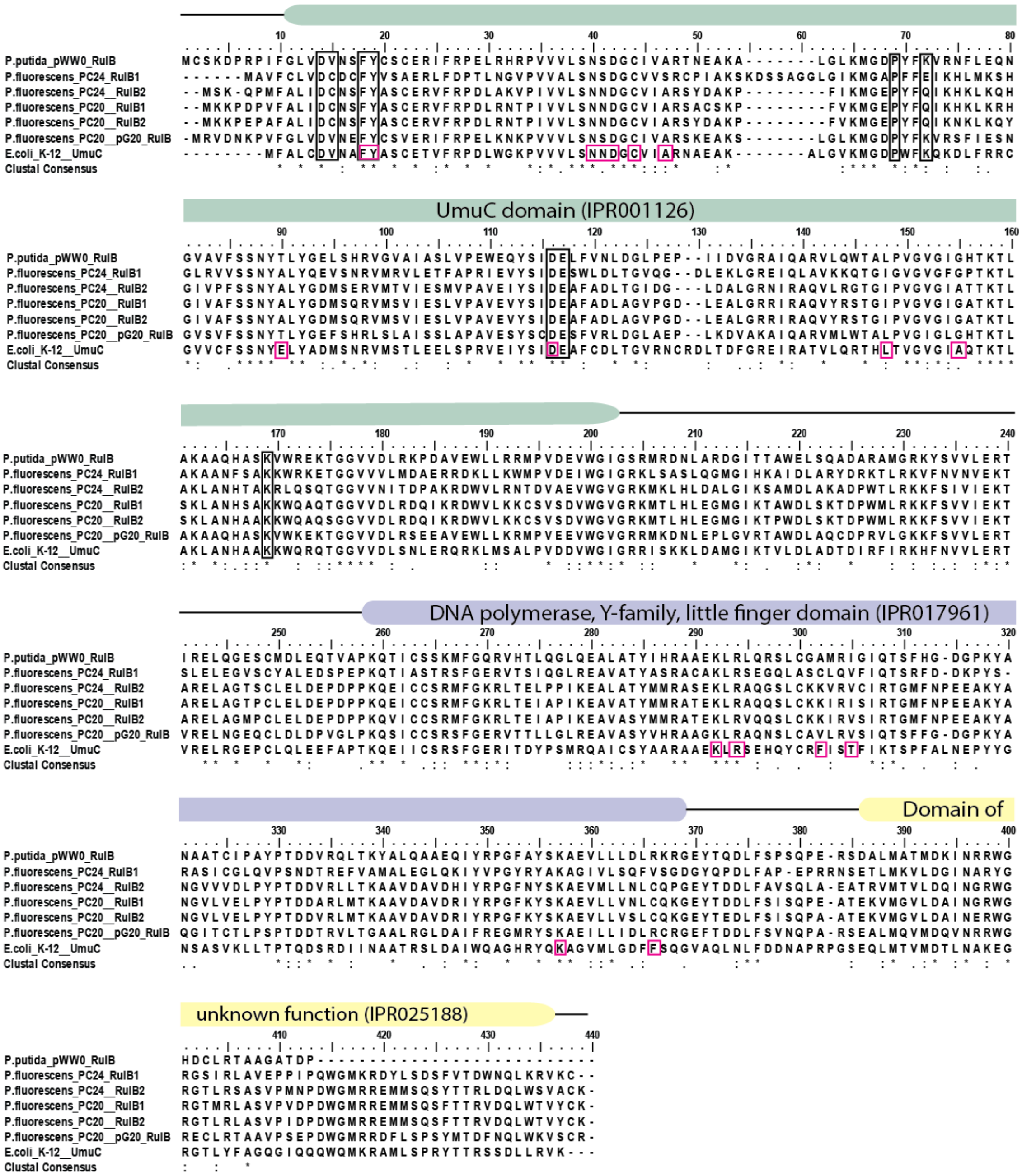

S3 Figure. Multiple sequence alignment of RulB homologs from *Pseudomonas fluorescens* PC20, PC24; *Pseudomonas putida* plasmid pWW0 RulB (Q8VMP6) and *E. coli* K-12 UmuC (P04152). Black boxes indicate the putative active site residues according to NCBI Conserved Protein Domain Family database (source cd00424; superfamily cl12025; PolY_Pol_V_umuC; [1]. Residues surrounded by pink boxes have been shown to be important in the UmuDC mediated mutagenesis [2-10]. Sequences were aligned with ClustalX2 and domains were marked according to Protein sequence analysis and classification portal Interpro (http://www.ebi.ac.uk/interpro/) based on the sequence of UmuC (P04152).

1. Marchler-Bauer A, Derbyshire MK, Gonzales NR, Lu S, Chitsaz F, Geer LY, et al. (2015) CDD: NCBI's conserved domain database. Nucleic Acids Res 43: D222-226.
2. Koch WH, Ennis DG, Levine AS, Woodgate R (1992) *Escherichia coli umuDC* mutants: DNA sequence alterations and UmuD cleavage. Mol Gen Genet 233: 443-448.
3. Kato T, Shinoura Y (1977) Isolation and characterization of mutants of *Escherichia coli* deficient in induction of mutations by ultraviolet light. Mol Gen Genet 156: 121-131.
4. Steinborn G (1978) Uvm mutants of *Escherichia coli* K12 deficient in UV mutagenesis. I. Isolation of *uvm* mutants and their phenotypical characterization in DNA repair and mutagenesis. Mol Gen Genet 165: 87-93.
5. Woodgate R, Singh M, Kulaeva OI, Frank EG, Levine AS, Koch WH (1994) Isolation and characterization of novel plasmid-encoded *umuC* mutants. J Bacteriol 176: 5011-5021.
6. Sutton MD, Walker GC (2001) *umuDC*-mediated cold sensitivity is a manifestation of functions of the UmuD(2)C complex involved in a DNA damage checkpoint control. J Bacteriol 183: 1215-1224.
7. Shurtleff BW, Ollivierre JN, Tehrani M, Walker GC, Beuning PJ (2009) Steric gate variants of UmuC confer UV hypersensitivity on *Escherichia coli*. J Bacteriol 191: 4815-4823.
8. Hawver LA, Gillooly CA, Beuning PJ (2011) Characterization of *Escherichia coli* UmuC active-site loops identifies variants that confer UV hypersensitivity. J Bacteriol 193: 5400-5411.
9. Vaisman A, Kuban W, McDonald JP, Karata K, Yang W, Goodman MF, et al. (2012) Critical amino acids in *Escherichia coli* UmuC responsible for sugar discrimination and base-substitution fidelity. Nucleic Acids Res 40: 6144-6157.
10. Boudsocq F, Ling H, Yang W, Woodgate R (2002) Structure-based interpretation of missense mutations in Y-family DNA polymerases and their implications for polymerase function and lesion bypass. DNA Repair (Amst) 1: 343-358.
