# Supplementary material for: Contribution of increased mutagenesis to the evolution of pollutants-degrading indigenous bacteria
Source: PLoS One. 2017 Aug 4;12(8):e0182484. doi: 10.1371/journal.pone.0182484 (PMC5544203; doi:10.1371/journal.pone.0182484)
Supplement: S4 Fig — PC24, Pseudomonas putida KT2440 DnaE2/ImuC (Q88182), and Mycobacterium tuberculosis (A0A089QXJ1). Black boxes indicate the putative active site residues according to NCBI Conserved Protein Domain Family database (source cd07431; superfamily cl23724; PHP_PolIIIA_DnaE2; [95]. Residues shaded gray represent active-site amino acids which are highly conserved among Y-family polymerases [107]. Residues underlined with red are conserved active site regions according to the study by [108]. Sequences were aligned with ClustalX2 and domains were marked according to Protein sequence analysis and classification portal Interpro (http://www.ebi.ac.uk/interpro/) based on the sequence of P. putida KT2440 DnaE2/ImuC (Q88182). Region underlined with black is a C-terminal amino acid motif -[S/T/G]R[D/N]F[D/R/H]- highly conserved in DnaE2-type proteins [107]. (PDF) [file pone.0182484.s005.pdf]

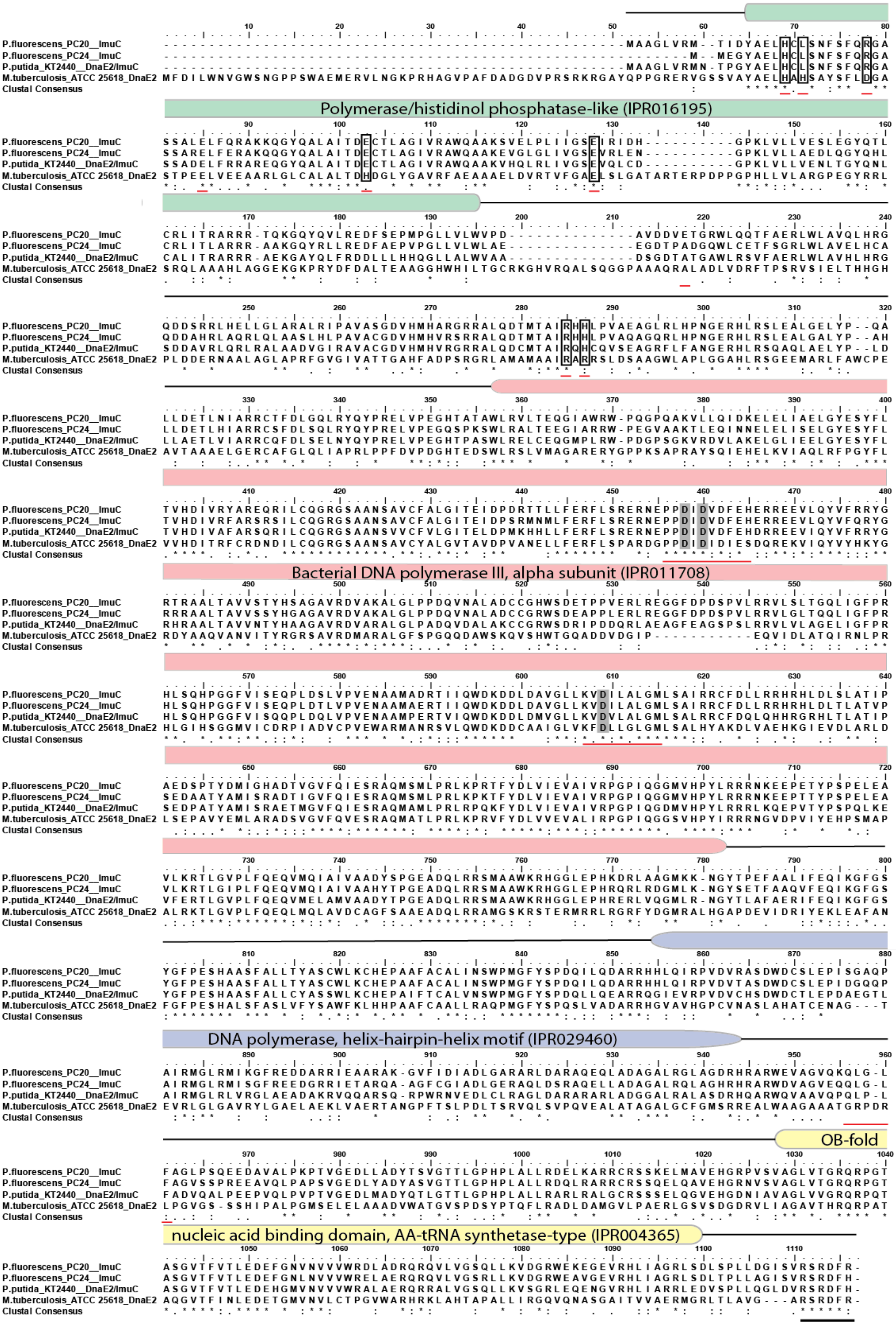
P.fluorescens_PC20__ImuC
P.fluorescens_PC24__ImuC
P.putida_KT2440__DnaE2/ImuC
M.tuberculosis_ATCC 25618_DnaE2
Clustal Consensus
Polymerase/histidinol phosphatase-like (IPR016195)
Bacterial DNA polymerase III, alpha subunit (IPR011708)
DNA polymerase, helix-hairpin-helix motif (IPR029460)
OB-fold
nucleic acid binding domain, AA-tRNA synthetase-type (IPR004365)

S4 Figure. Multiple sequence alignment of ImuC/DnaE2 homologs from *Pseudomonas fluorescens* PC20. PC24, *Pseudomonas putida* KT2440 DnaE2/ImuC (Q88182), and *Mycobacterium tuberculosis* (A0A089QXJ1). Black boxes indicate the putative active site residues according to NCBI Conserved Protein Domain Family database (source cd07431; superfamily cl23724; PHP_PolIIIA_DnaE2; [1]. Residues shaded gray represent active-site amino acids which are highly conserved among Y-family polymerases [2]. Residues underlined with red are conserved active site regions according to the study by [3]. Sequences were aligned with ClustalX2 and domains were marked according to Protein sequence analysis and classification portal Interpro (http://www.ebi.ac.uk/interpro/) based on the sequence of *P. putida* KT2440 DnaE2/ImuC (Q88182). Region underlined with black is a C-terminal amino acid motif -[S/T/G]R[D/N]F[D/R/H]- highly conserved in DnaE2-type proteins [2].

1. Marchler-Bauer A, Derbyshire MK, Gonzales NR, Lu S, Chitsaz F, Geer LY, et al. (2015) CDD: NCBI's conserved domain database. Nucleic Acids Res 43: D222-226.
2. Kim DR, Pritchard AE, McHenry CS (1997) Localization of the active site of the alpha subunit of the *Escherichia coli* DNA polymerase III holoenzyme. J Bacteriol 179: 6721-6728.
3. Timinskas K, Balvociute M, Timinskas A, Venclovas C (2014) Comprehensive analysis of DNA polymerase III alpha subunits and their homologs in bacterial genomes. Nucleic Acids Res 42: 1393-1413.
