# Supplementary material for: Contribution of increased mutagenesis to the evolution of pollutants-degrading indigenous bacteria
Source: PLoS One. 2017 Aug 4;12(8):e0182484. doi: 10.1371/journal.pone.0182484 (PMC5544203; doi:10.1371/journal.pone.0182484)
Supplement: S5 Fig — The -35 and -10 hexamers of the promoters are marked by black boxes and transcriptional start site for umuDC promoter [109] is marked with red box. LexA-binding consensus sequence is aligned on the last row [110]. Sequences were aligned with ClustalX2. (PDF) [file pone.0182484.s006.pdf]

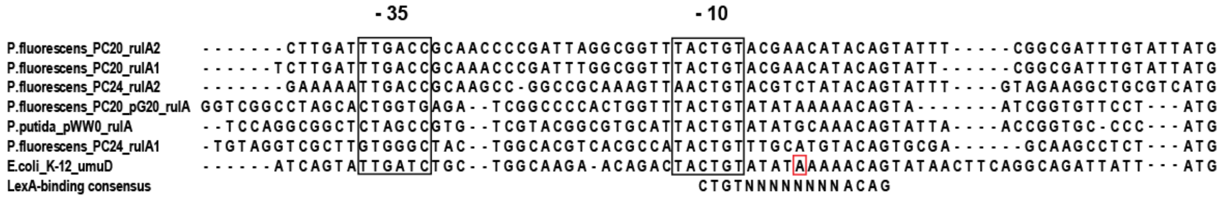

S5 Figure. Multiple sequence alignment of putative promoter regions of *rulAB* genes and *E. coli umuDC* genes. The -35 and -10 hexamers of the promoters are marked by black boxes and transcriptional start site for *umuDC* promoter [1] is marked with red box. LexA-binding consensus sequence is aligned on the last row [2]. Sequences were aligned with ClustalX2.

1. Kitagawa Y, Akaboshi E, Shinagawa H, Horii T, Ogawa H, Kato T. (1985) Structural analysis of the *umu* operon required for inducible mutagenesis in *Escherichia coli*. Proc Natl Acad Sci U S A 82: 4336-4340.
2. Wertman KF, Mount DW (1985) Nucleotide sequence binding specificity of the LexA repressor of *Escherichia coli* K-12. J Bacteriol 163: 376-384.
