# Supplementary material for: Contribution of increased mutagenesis to the evolution of pollutants-degrading indigenous bacteria
Source: PLoS One. 2017 Aug 4;12(8):e0182484. doi: 10.1371/journal.pone.0182484 (PMC5544203; doi:10.1371/journal.pone.0182484)
Supplement: S6 Fig — The -35 and -10 hexamers of the promoters are marked by black boxes. LexA2-binding consensus sequence is aligned on the last row [43]. Sequences were aligned with ClustalX2. (PDF) [file pone.0182484.s007.pdf]

```
                                               -35                    -10
P.fluorescens_PC24_lexA2imuABC  -GACTGGCGGCTA[TTGTCA]GGAATTTCCCCAAAAGAG[TACAAAT]GTACTCCGATG
P.fluorescens_PC20_lexA2imuABC  GGATTGACGGTCC[TTGCCA]GGAAAA-CACCAAAAGAG[TACAAAT]GTACTCC-ATG
P.putida_KT2440_lexA2imuABC     -CGACGAAGGGGG[CTGTTC]AGCGTGCTCCGAAAAGAG[TACAAAT]GTGCTCC-ATG
LexA2-binding consensus                                             GTACNNNNGTGC
```

S6 Figure. Multiple sequence alignment of putative promoter regions of *imuABC*. The -35 and -10 hexamers of the promoters are marked by black boxes. LexA2-binding consensus sequence is aligned on the last row [1]. Sequences were aligned with ClustalX2.

1. Abella M, Erill I, Jara M, Mazon G, Campoy S, Barbe J (2004) Widespread distribution of a *lexA*-regulated DNA damage-inducible multiple gene cassette in the *Proteobacteria* phylum. Mol Microbiol 54: 212-222.
