# Supplementary material for: Contribution of increased mutagenesis to the evolution of pollutants-degrading indigenous bacteria
Source: PLoS One. 2017 Aug 4;12(8):e0182484. doi: 10.1371/journal.pone.0182484 (PMC5544203; doi:10.1371/journal.pone.0182484)
Supplement: S7 Fig — The phylogenetic trees of UvrA (A), UvrB (B), UvrC (C) and UvrD (D) protein sequences. Sequences were aligned with ClustalX2 and further visualised with TreeViewX. Numbers at branch nodes indicate bootstrapping values for 1000 bootstrap replicates. Values under 500 were removed. The identifiers of the aligned sequences are presented in S7 Table. (PDF) [file pone.0182484.s008.pdf]

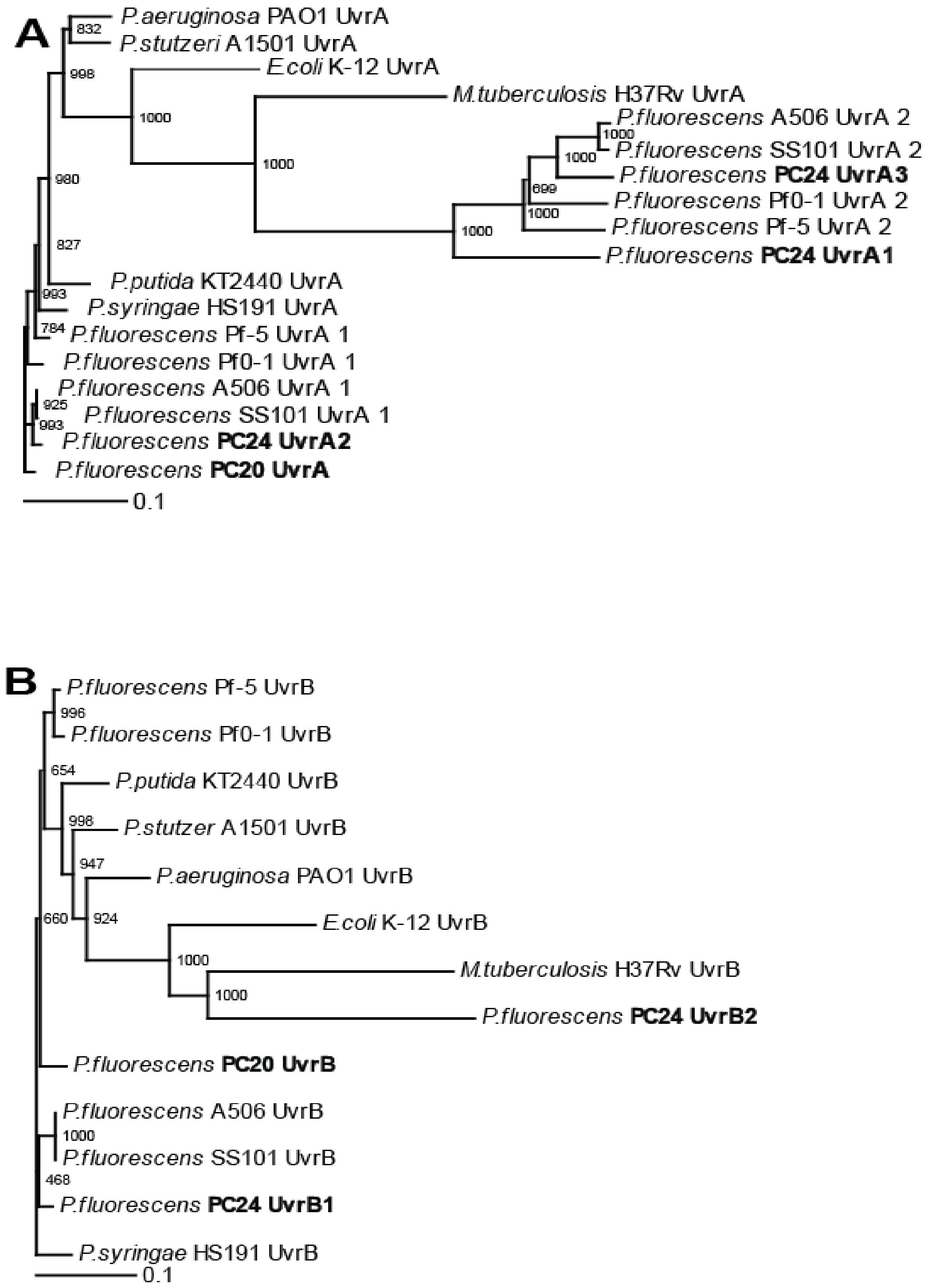
A
P.aeruginosa PAO1 UvrA
P.stutzeri A1501 UvrA
E.coli K-12 UvrA
M.tuberculosis H37Rv UvrA
P.fluorescens A506 UvrA 2
P.fluorescens SS101 UvrA 2
P.fluorescens PC24 UvrA3
P.fluorescens Pf0-1 UvrA 2
P.fluorescens Pf-5 UvrA 2
P.fluorescens PC24 UvrA1
P.putida KT2440 UvrA
P.syringae HS191 UvrA
P.fluorescens Pf-5 UvrA 1
P.fluorescens Pf0-1 UvrA 1
P.fluorescens A506 UvrA 1
P.fluorescens SS101 UvrA 1
P.fluorescens PC24 UvrA2
P.fluorescens PC20 UvrA
832
998
1000
1000
1000
1000
1000
699
1000
980
827
993
784
925
993
0.1
B
P.fluorescens Pf-5 UvrB
P.fluorescens Pf0-1 UvrB
P.putida KT2440 UvrB
P.stutzer A1501 UvrB
P.aeruginosa PAO1 UvrB
E.coli K-12 UvrB
M.tuberculosis H37Rv UvrB
P.fluorescens PC24 UvrB2
P.fluorescens PC20 UvrB
P.fluorescens A506 UvrB
P.fluorescens SS101 UvrB
P.fluorescens PC24 UvrB1
P.syringae HS191 UvrB
996
654
998
947
660
924
1000
1000
1000
468
0.1

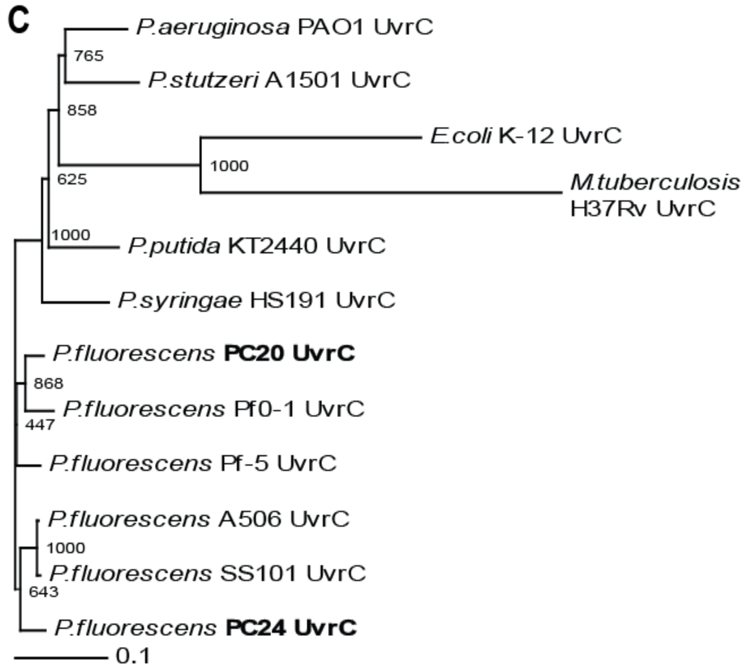

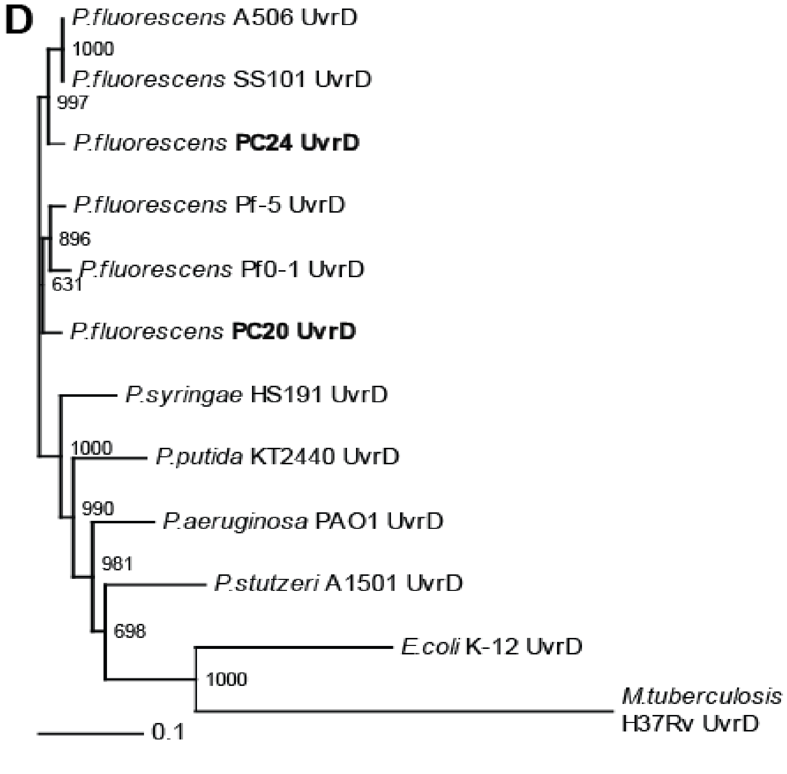

S7 Figure. The phylogenetic trees of UvrA (A), UvrB (B), UvrC (C) and UvrD (D) protein sequences. Sequences were aligned with ClustalX2 and further visualised with TreeViewX. Numbers at branch nodes indicate bootstrapping values for 1000 bootstrap replicates. Values under 500 were removed. The identifiers of the aligned sequences are presented in S7 Table.
