# Supplementary material for: Contribution of increased mutagenesis to the evolution of pollutants-degrading indigenous bacteria
Source: PLoS One. 2017 Aug 4;12(8):e0182484. doi: 10.1371/journal.pone.0182484 (PMC5544203; doi:10.1371/journal.pone.0182484)
Supplement: S1 Table — Three laboratory strains were studied as a comparison in addition to the environmental strains. All of the mutant frequencies were compared to P. putida PaW85 laboratory reference strain with Kruskal-Wallis test. The statistically significant p-values according to Benjamini-Hochberg procedure are indicated with red (FRD = 0.05). (DOCX) [file pone.0182484.s009.docx]

**S1 Table.** **The median values of the frequency of spontaneous Rif^r^ mutants.** Three laboratory strains were studied as a comparison in addition to the environmental strains. All of the mutant frequencies were compared to *P. putida* PaW85 laboratory reference strain with Kruskal-Wallis test. The statistically significant p-values according to Benjamini-Hochberg procedure are indicated with red (FRD = 0.05).

| Strain | Valid N | Median | Lower quartile | Upper quartile | p-values |
| --- | --- | --- | --- | --- | --- |
| PaW85 | 78 | 1.63E-08 | 7.94E-09 | 2.91E-08 |  |
| PaWrulAB | 14 | 8.52E-09 | 5.40E-09 | 1.78E-08 | 1.0000 |
| PaW1 | 14 | 6.89E-09 | 4.58E-09 | 1.03E-08 | 1.0000 |
| 2A20 | 34 | 3.60E-08 | 2.51E-08 | 7.00E-08 | 1.0000 |
| 2A38 | 12 | 5.30E-08 | 4.01E-09 | 7.88E-08 | 1.0000 |
| 2A54 | 13 | 2.72E-08 | 2.38E-08 | 3.21E-08 | 1.0000 |
| 2Anah4 | 13 | 4.09E-08 | 2.19E-08 | 5.91E-08 | 1.0000 |
| 2B45 | 13 | 2.52E-07 | 6.00E-08 | 3.91E-07 | 1.0000 |
| 2C23 | 13 | 2.51E-07 | 1.70E-07 | 3.62E-07 | 0.0094 |
| 2C41 | 14 | 5.29E-08 | 3.06E-08 | 1.07E-07 | 1.0000 |
| 2C56 | 10 | 1.88E-09 | 1.25E-09 | 4.62E-09 | 0.3945 |
| 2C63 | 14 | 3.97E-08 | 2.76E-08 | 9.26E-08 | 1.0000 |
| 2D47 | 15 | 1.16E-08 | 5.16E-09 | 1.30E-08 | 1.0000 |
| 2D61 | 48 | 9.49E-08 | 6.52E-08 | 1.45E-07 | <0.0001 |
| 2D66 | 14 | 4.83E-08 | 3.47E-08 | 6.46E-08 | 1.0000 |
| 2D67 | 32 | 2.56E-09 | 5.26E-10 | 5.71E-09 | <0.00001 |
| C52 | 13 | 9.45E-08 | 6.99E-08 | 1.10E-07 | 0.3528 |
| C70 | 13 | 1.62E-09 | 1.00E-09 | 5.00E-09 | 0.0039 |
| D113 | 15 | 3.79E-08 | 2.80E-08 | 5.51E-08 | 1.0000 |
| D14 | 35 | 2.35E-09 | 0.00E+00 | 4.29E-09 | <0.0001 |
| D28 | 14 | 1.64E-08 | 1.12E-08 | 3.28E-08 | 1.0000 |
| D2RT | 13 | 0.00E+00 | 0.00E+00 | 0.00E+00 | <0.0001 |
| D3 | 15 | 1.62E-08 | 8.89E-09 | 2.89E-08 | 1.0000 |
| D45 | 13 | 6.90E-09 | 4.75E-09 | 1.58E-08 | 1.0000 |
| D66v | 35 | 5.00E-09 | 1.54E-09 | 1.33E-08 | 0.1119 |
| D67 | 13 | 1.11E-08 | 6.79E-09 | 1.20E-08 | 1.0000 |
| Hd1 | 14 | 4.66E-09 | 3.29E-09 | 1.04E-08 | 1.0000 |
| Hd16 | 13 | 4.22E-09 | 3.57E-09 | 7.11E-09 | 0.5594 |
| Hd6 | 15 | 4.27E-09 | 2.09E-09 | 8.37E-09 | 0.1525 |
| Hp2 | 15 | 9.46E-09 | 4.30E-09 | 1.63E-08 | 1.0000 |
| Hp5 | 14 | 1.56E-08 | 1.19E-08 | 1.96E-08 | 1.0000 |
| Hp6 | 13 | 6.18E-09 | 3.41E-09 | 8.50E-09 | 1.0000 |
| Nah4 | 11 | 2.81E-09 | 1.08E-09 | 7.57E-09 | 0.0785 |
| P3 | 15 | 5.96E-09 | 4.00E-09 | 1.26E-08 | 1.0000 |
| P37 | 13 | 7.53E-09 | 3.90E-09 | 1.06E-08 | 1.0000 |
| P4 | 12 | 2.20E-09 | 9.52E-10 | 5.94E-09 | 0.0130 |
| P48 | 13 | 9.67E-09 | 8.67E-09 | 1.67E-08 | 1.0000 |
| P49 | 13 | 4.89E-09 | 3.11E-09 | 9.27E-09 | 1.0000 |
| P6 | 13 | 6.85E-09 | 4.93E-09 | 1.07E-08 | 1.0000 |
| P69 | 13 | 3.02E-09 | 2.00E-09 | 6.04E-09 | 0.0226 |
| P85 | 13 | 1.57E-08 | 1.40E-08 | 2.02E-08 | 1.0000 |
| P86 | 14 | 2.26E-09 | 1.38E-09 | 4.50E-09 | 0.0008 |
| P94 | 15 | 3.67E-09 | 2.00E-09 | 6.51E-09 | 0.0227 |
| PC13 | 24 | 8.77E-09 | 5.48E-09 | 1.50E-08 | 1.0000 |
| PC14 | 24 | 1.48E-08 | 1.14E-08 | 2.16E-08 | 1.0000 |
| PC15 | 14 | 1.62E-08 | 8.35E-09 | 1.94E-08 | 1.0000 |
| PC16 | 48 | 5.39E-09 | 3.01E-09 | 1.04E-08 | 0.0026 |
| PC17 | 13 | 1.21E-08 | 5.00E-09 | 1.65E-08 | 1.0000 |
| PC18 | 38 | 6.64E-09 | 2.60E-09 | 1.18E-08 | 0.7535 |
| PC20 | 49 | 9.63E-09 | 4.26E-09 | 3.03E-08 | 1.0000 |
| PC24 | 49 | 1.73E-08 | 9.52E-09 | 2.91E-08 | 1.0000 |
| PC30 | 13 | 1.50E-08 | 1.13E-08 | 2.10E-08 | 1.0000 |
| PC34 | 14 | 6.44E-09 | 3.87E-09 | 1.24E-08 | 1.0000 |
| PC36 | 15 | 1.08E-08 | 6.33E-09 | 2.23E-08 | 1.0000 |
| PC38 | 15 | 3.45E-09 | 1.90E-09 | 7.94E-09 | 0.0458 |
| PC39 | 13 | 8.60E-09 | 7.50E-09 | 9.50E-09 | 1.0000 |
