# Supplementary material for: Contribution of increased mutagenesis to the evolution of pollutants-degrading indigenous bacteria
Source: PLoS One. 2017 Aug 4;12(8):e0182484. doi: 10.1371/journal.pone.0182484 (PMC5544203; doi:10.1371/journal.pone.0182484)
Supplement: S2 Table — (DOCX) [file pone.0182484.s010.docx]

**S1 Table.** **The minimal inhibitory concentrations of representatives of the indigenous strains and the reference strain *P. putida* PaW85.**

| Strain | MIC Rif (µg/ml) | MIC Sm (µg/ml) |
| --- | --- | --- |
| PaW85 | 6 | 100 |
| C70 | 3 | 50 |
| D66v | 0.75 | 25 |
| 2C23 | 1.5 | 50 |
| 2B45 | 1.5 | 12.5 |
| P86 | 3 | 50 |
| P4 | 0.32 | 50 |
| PC20 | 3 | 12.5 |
| PC16 | 6 | 100 |
| 2D61 | 12 | 100 |
