# Supplementary material for: Contribution of increased mutagenesis to the evolution of pollutants-degrading indigenous bacteria
Source: PLoS One. 2017 Aug 4;12(8):e0182484. doi: 10.1371/journal.pone.0182484 (PMC5544203; doi:10.1371/journal.pone.0182484)
Supplement: S3 Table — The statistically significant p-values according to Benjamini-Hochberg procedure are indicated with red (FRD = 0.05). (DOCX) [file pone.0182484.s011.docx]

**S3 Table.** **The results of Mann-Whitney U test for comparing the appearance frequency of mutants for two different antibiotics (rifampicin and streptomycin) within one strain.** The statistically significant p-values according to Benjamini-Hochberg procedure are indicated with red (FRD = 0.05).

| Strain | P-values |
| --- | --- |
| PaW85 | 0.1746 |
| C70 | 0.0083 |
| D66v | 0.0002 |
| 2C23 | 0.0001 |
| 2B45 | 0.2539 |
| P86 | <0.0001 |
| P4 | <0.0001 |
| PC20 | <0.0001 |
| PC16 | <0.0001 |
| 2D61 | <0.0001 |
