# Supplementary material for: Contribution of increased mutagenesis to the evolution of pollutants-degrading indigenous bacteria
Source: PLoS One. 2017 Aug 4;12(8):e0182484. doi: 10.1371/journal.pone.0182484 (PMC5544203; doi:10.1371/journal.pone.0182484)
Supplement: S4 Table — The statistically significant p-values according to Benjamini-Hochberg procedure are indicated with red (FRD = 0.05). (DOCX) [file pone.0182484.s012.docx]

**S4 Table.** **Comparison of Sm^r^ mutant frequencies with Kruskal-Wallis test against PaW85.** The statistically significant p-values according to Benjamini-Hochberg procedure are indicated with red (FRD = 0.05).

| Strain | p-values |
| --- | --- |
| C70 | 0.0014 |
| D66v | 0.0037 |
| 2C23 | 1.0000 |
| 2B45 | 1.0000 |
| P86 | 0.0003 |
| P4 | <0.0001 |
| PC20 | 0.1985 |
| PC16 | 0.0032 |
| 2D61 | 1.0000 |
