# Supplementary material for: Contribution of increased mutagenesis to the evolution of pollutants-degrading indigenous bacteria
Source: PLoS One. 2017 Aug 4;12(8):e0182484. doi: 10.1371/journal.pone.0182484 (PMC5544203; doi:10.1371/journal.pone.0182484)
Supplement: S5 Table — Underlined are the strains with at least 10 fold higher UV-tolerance (UV- 20 J/m2) compared to PaW85. Kruskal-Wallis test was performed to compare UV-tolerance (UV- 20 J/m2) of the studied strains against PaW1, which possesses PolV coding operon rulAB. The statistically significant p-values according to Benjamini-Hochberg procedure are indicated with red (FRD = 0.05). (DOCX) [file pone.0182484.s013.docx]

**S5 Table.** **The survival percentage of bacteria after UV-C irradiation.** Underlined are the strains with at least 10 fold higher UV-tolerance (UV- 20 J/m^2^) compared to PaW85. Kruskal-Wallis test was performed to compare UV-tolerance (UV- 20 J/m^2^) of the studied strains against PaW1, which possesses PolV coding operon *rulAB.* The statistically significant p-values according to Benjamini-Hochberg procedure are indicated with red (FRD = 0.05).

| Strain | UV- 10 J/m^2^ | | | UV- 20 J/m2 | | | |
| --- | --- | --- | --- | --- | --- | --- | --- |
|  | Valid N | Mean | Std.Dev. | Valid N | Mean | Std.Dev. | P-value |
| PaW85 | 28 | 16.23 | 22.47 | 28 | 0.23 | 0.40 | 0.001 |
| PaWrulAB | 28 | 47.81 | 38.50 | 28 | 5.13 | 5.43 | 1.000 |
| PaW1 | 28 | 46.28 | 24.97 | 28 | 8.14 | 7.90 | - |
| 2A20 | 6 | 26.11 | 14.10 | 6 | 5.82 | 3.59 | 1.000 |
| 2A38 | 8 | 98.64 | 34.23 | 8 | 77.41 | 30.27 | 1.000 |
| 2A54 | 6 | 83.29 | 33.54 | 6 | 44.85 | 16.95 | 1.000 |
| 2ANah4 | 6 | 40.98 | 24.13 | 6 | 18.93 | 11.72 | 1.000 |
| 2C23 | 6 | 32.59 | 10.82 | 6 | 13.40 | 11.23 | 1.000 |
| 2C41 | 5 | 61.76 | 35.42 | 5 | 40.82 | 32.99 | 1.000 |
| 2C63 | 6 | 46.27 | 22.93 | 6 | 27.78 | 17.45 | 1.000 |
| 2D61 | 7 | 26.35 | 14.25 | 7 | 1.89 | 1.05 | 1.000 |
| 2D66 | 6 | 43.96 | 22.70 | 6 | 28.80 | 20.60 | 1.000 |
| 2D67 | 12 | 60.84 | 48.09 | 12 | 36.89 | 47.53 | 1.000 |
| C52 | 10 | 89.82 | 39.90 | 10 | 58.71 | 34.44 | 1.000 |
| D113 | 5 | 6.82 | 2.53 | 5 | 0.31 | 0.30 | 1.000 |
| D14 | 7 | 25.66 | 22.68 | 7 | 2.13 | 2.36 | 1.000 |
| D28 | 6 | 0.00 | 0.00 | 6 | 0.00 | 0.00 | 0.001 |
| D3 | 7 | 6.72 | 5.12 | 7 | 0.31 | 0.24 | 1.000 |
| D45 | 8 | 8.22 | 20.92 | 8 | 8.77 | 18.07 | 0.933 |
| D66v | 8 | 83.19 | 76.63 | 8 | 35.49 | 20.71 | 1.000 |
| Hd1 | 7 | 5.45 | 5.30 | 7 | 0.09 | 0.11 | 0.415 |
| Hd16 | 5 | 35.03 | 11.47 | 5 | 4.51 | 1.46 | 1.000 |
| Hd6 | 7 | 10.45 | 9.01 | 7 | 0.38 | 0.37 | 1.000 |
| Hp2 | 6 | 47.00 | 41.72 | 6 | 13.76 | 14.10 | 1.000 |
| Hp5 | 8 | 46.00 | 29.34 | 8 | 7.86 | 4.93 | 1.000 |
| Hp6 | 6 | 37.45 | 14.88 | 6 | 10.88 | 4.62 | 1.000 |
| P3 | 8 | 14.79 | 19.07 | 8 | 1.54 | 3.42 | 1.000 |
| P4 | 5 | 0.07 | 0.06 | 5 | 0.01 | 0.01 | 0.132 |
| P48 | 8 | 42.11 | 41.48 | 8 | 10.74 | 6.71 | 1.000 |
| P49 | 5 | 3.06 | 2.50 | 5 | 0.48 | 0.52 | 1.000 |
| P6 | 7 | 13.37 | 17.82 | 7 | 0.26 | 0.25 | 1.000 |
| P69 | 6 | 14.95 | 9.31 | 6 | 0.16 | 0.15 | 1.000 |
| P85 | 7 | 11.97 | 8.22 | 7 | 0.88 | 0.60 | 1.000 |
| P86 | 6 | 35.67 | 24.89 | 7 | 8.35 | 6.26 | 1.000 |
| P87 | 5 | 9.55 | 2.84 | 5 | 0.46 | 0.44 | 1.000 |
| P94 | 5 | 48.11 | 18.29 | 5 | 7.51 | 6.69 | 1.000 |
| PC13 | 6 | 0.03 | 0.04 | 6 | 0.00 | 0.00 | 0.003 |
| PC14 | 6 | 0.21 | 0.21 | 6 | 0.01 | 0.01 | 0.018 |
| PC15 | 6 | 0.16 | 0.22 | 6 | 0.00 | 0.00 | 0.002 |
| PC16 | 7 | 0.23 | 0.24 | 7 | 0.02 | 0.03 | 0.018 |
| PC17 | 6 | 38.33 | 34.88 | 6 | 5.26 | 5.47 | 1.000 |
| PC18 | 13 | 12.94 | 11.96 | 13 | 0.62 | 1.20 | 0.163 |
| PC20 | 7 | 21.05 | 11.09 | 7 | 1.48 | 1.91 | 1.000 |
| PC24 | 7 | 3.73 | 3.43 | 7 | 0.05 | 0.07 | 0.087 |
| PC30 | 6 | 0.15 | 0.12 | 6 | 0.00 | 0.00 | 0.003 |
| PC34 | 6 | 0.00 | 0.00 | 6 | 0.00 | 0.00 | <0.0001 |
| PC36 | 6 | 0.81 | 0.86 | 6 | 0.03 | 0.05 | 0.044 |
| PC38 | 6 | 20.28 | 14.16 | 6 | 0.92 | 1.19 | 1.000 |
| PC39 | 6 | 1.47 | 0.91 | 6 | 0.01 | 0.01 | 0.014 |
