# Supplementary material for: Contribution of increased mutagenesis to the evolution of pollutants-degrading indigenous bacteria
Source: PLoS One. 2017 Aug 4;12(8):e0182484. doi: 10.1371/journal.pone.0182484 (PMC5544203; doi:10.1371/journal.pone.0182484)
Supplement: S6 Table — The Mann-Whitney U test was performed to distinguish statistically significant differences of UV-induced and spontaneous Rifr mutant frequencies. The statistically significant p-values according to Benjamini-Hochberg procedure are indicated with red (FRD = 0.05). The difference between spontaneous and UV-induced mutant frequency is presented as fold of induction. Underlined are the strains with at least 10 fold higher tolerance against UV irradiation (UV-20 J/m2) compared to PaW85. (DOCX) [file pone.0182484.s014.docx]

**S6 Table.** **The UV-induced (100 J/m^2^) Rif^r^ mutant frequencies.** The Mann-Whitney U test was performed to distinguish differences of UV-induced and spontaneous Rif^r^ mutant frequencies. The statistically significant p-values according to Benjamini-Hochberg procedure are indicated with red (FRD = 0.05). The difference between spontaneous and UV-induced mutant frequency is presented as fold of induction. Underlined are the strains with at least 10 fold higher tolerance against UV irradiation (UV-20 J/m^2^) compared to PaW85.

| Strain | Valid N | Median | Lower quartile | Upper quartile | p-values | Fold of induction |
| --- | --- | --- | --- | --- | --- | --- |
| PaW85 | 39 | 1.27E-08 | 7.72E-09 | 2.03E-08 | 0.275 | 0.78 |
| PaWrulAB | 19 | 4.75E-08 | 1.27E-08 | 2.33E-07 | 0.003 | 5.58 |
| PaW1 | 39 | 1.22E-07 | 4.61E-08 | 4.00E-07 | <0.0001 | 17.71 |
| 2A20 | 14 | 2.88E-07 | 1.75E-07 | 3.68E-07 | <0.0001 | 8.00 |
| 2A38 | 19 | 2.03E-06 | 1.03E-06 | 4.88E-06 | <0.0001 | 38.27 |
| 2A54 | 15 | 9.28E-07 | 8.48E-07 | 1.15E-06 | <0.0001 | 34.16 |
| 2Anah4 | 19 | 2.44E-07 | 1.89E-07 | 3.24E-07 | <0.0001 | 5.96 |
| 2C23 | 20 | 8.90E-08 | 6.24E-08 | 1.08E-07 | <0.0001 | 0.36 |
| 2C41 | 19 | 3.46E-07 | 2.58E-07 | 6.04E-07 | <0.0001 | 6.54 |
| 2C63 | 14 | 6.22E-07 | 3.85E-07 | 7.30E-07 | <0.0001 | 15.65 |
| 2D61 | 19 | 1.47E-07 | 6.57E-08 | 3.00E-07 | 0.074 | 1.55 |
| 2D66 | 20 | 5.13E-07 | 3.96E-07 | 6.48E-07 | <0.0001 | 10.62 |
| 2D67 | 24 | 7.34E-09 | 2.12E-09 | 4.63E-08 | 0.005 | 2.86 |
| C52 | 18 | 2.51E-06 | 3.30E-07 | 4.25E-06 | <0.0001 | 26.56 |
| D14 | 14 | 0.00 | 0.00 | 0.00 | 0.015 | 0.00 |
| D66v | 24 | 5.10E-07 | 2.63E-07 | 6.38E-07 | <0.0001 | 102.00 |
| Hd16 | 19 | 1.19E-07 | 8.40E-08 | 1.56E-07 | <0.0001 | 28.15 |
| Hp2 | 19 | 1.42E-07 | 9.77E-08 | 1.62E-07 | <0.0001 | 15.01 |
| Hp5 | 19 | 2.12E-07 | 1.26E-07 | 2.28E-07 | <0.0001 | 13.59 |
| Hp6 | 33 | 5.66E-08 | 3.13E-08 | 7.92E-08 | <0.0001 | 9.16 |
| P48 | 19 | 3.36E-07 | 2.54E-08 | 5.11E-07 | 0.005 | 34.76 |
| P86 | 19 | 9.30E-08 | 7.57E-09 | 1.33E-07 | <0.0001 | 41.07 |
| P94 | 19 | 1.06E-07 | 6.00E-08 | 1.70E-07 | <0.0001 | 28.91 |
| PC16 | 19 | 1.35E-08 | 4.00E-09 | 2.70E-08 | 0.015 | 2.51 |
| PC17 | 19 | 1.26E-08 | 2.00E-09 | 5.13E-08 | 0.520 | 1.04 |
| PC18 | 19 | 3.73E-08 | 4.67E-09 | 1.27E-07 | 0.007 | 5.62 |
| PC20 | 14 | 0.00 | 0.00 | 0.00 | <0.0001 | 0.00 |
| PC24 | 19 | 7.79E-08 | 1.16E-08 | 1.17E-07 | 0.009 | 4.51 |
