# Supplementary material for: Contribution of increased mutagenesis to the evolution of pollutants-degrading indigenous bacteria
Source: PLoS One. 2017 Aug 4;12(8):e0182484. doi: 10.1371/journal.pone.0182484 (PMC5544203; doi:10.1371/journal.pone.0182484)
Supplement: S7 Table — Protein sequences were translated based on the gene sequences listed below. Sequence ID-s were obtained from GenBank (https://www.ncbi.nlm.nih.gov/genbank/) and The European Nucleotide Archive (ENA, http://www.ebi.ac.uk/ena). (DOCX) [file pone.0182484.s015.docx]

**S7 Table.** **The sequences used in phylogenetic analyses and multiple sequence alignments.** Protein sequences were translated based on the gene sequences listed below. Sequence ID-s were obtained from GenBank (https://www.ncbi.nlm.nih.gov/genbank/) and The European Nucleotide Archive (ENA, <http://www.ebi.ac.uk/ena>).

| **Strain** | **Plasmid** | **GenBank acc.nr / ENA** | **Positions** | **Gene** |
| --- | --- | --- | --- | --- |
| *P.fluorescens* PC20 |  | KX893522 | 1571..4405 | *uvrA* |
|  |  | KX893523 | 1677..3692 | *uvrB* |
|  |  | KX893524 | 1497..3320 | *uvrC* |
|  |  | KX893525 | 993..3176 | *uvrD* |
|  |  | KX893518 | 1580..2008 | *rulA1* |
|  |  | KX893519 | 1862..2290 | *rulA2* |
|  |  | KX893518 | 1995..3275 | *rulB1* |
|  |  | KX893519 | 2277..3557 | *rulB2* |
|  |  | KX893520 | 3753..6851 | *imuC* |
|  | pG20 | KX893538 | 43407..43835 | *rulA_pG20_* |
|  | pG20 | KX893538 | 43825..45108 | *rulB_pG20_* |
| *P.fluorescens* PC24 |  | KX893537 | 1202..3853 | *uvrA1* |
|  |  | KX893531 | 1554..4388 | *uvrA2* |
|  |  | KX893532 | 1171.3708 | *uvrA3* |
|  |  | KX893533 | 1531..3546 | *uvrB1* |
|  |  | KX893534 | 1870..3846 | *uvrB2* |
|  |  | KX893535 | 626..2464 | *uvrC* |
|  |  | KX893536 | 1265..3448 | *uvrD* |
|  |  | KX893526 | 1482..1913 | *rulA1* |
|  |  | KX893527 | 2022..2447 | *rulA2* |
|  |  | KX893526 | 3719..5002 | *rulB1* |
|  |  | KX893527 | 2440..3714 | *rulB2* |
|  |  | KX893530 | 3816..6893 | *imuC* |
| *P.putida* KT2440 |  | NC_002947.4 | 566661..569495 c | *uvrA* |
|  |  |  | 2234890..2236905 | *uvrB* |
|  |  |  | 4633287..4635110 c | *uvrC* |
|  |  |  | 6100849..6103035 | *uvrD* |
|  |  |  | 3528184..3531285 | *dnaE2/imuC* |
| *E.coli* K-12 |  | NC_000913.3 | 4271049..4273871 c | *uvrA* |
|  |  |  | 813526..815547 | *uvrB* |
|  |  |  | 1992874..1994706 c | *uvrC* |
|  |  |  | 3997983..4000145 | *uvrD* |
|  |  |  | 1231186..1232454 | *umuC* |
|  |  |  | 1230767..1231186 | *umuD* |
| *M.tuberculosis* H37Rv |  | NC_000962.3 | 1843741..1846659 | *uvrA* |
|  |  |  | 1837075..1839171 | *uvrB* |
|  |  |  | 1594042..1595982 | *uvrC* |
|  |  |  | 1058260..1060575 | *uvrD* |
|  |  |  | 3781501..3784740 c | *dnaE2* |
| *P.fluorescens* Pf0-1 |  | NC_007492.2 | 5686728..5689562 | *uvrA1* |
|  |  |  | 3601777..3604410 c | *uvrA2* |
|  |  |  | 2137616..2139631 | *uvrB* |
|  |  |  | 3507583..3509406 c | *uvrC* |
|  |  |  | 6325758..6327941 | *uvrD* |
|  |  |  | 3614981..3618058 c | *dnaE2* |
| *P.fluorescens* Pf-5 |  | NC_004129.6 | 6343672..6346506 | *uvrA1* |
|  |  |  | 4281594..4284260 c | *uvrA2* |
|  |  |  | 2272690..2274705 | *uvrB* |
|  |  |  | 4108688..4110511 c | *uvrC* |
|  |  |  | 6999297..7001480 | *uvrD* |
|  |  |  | 5420936..5421361 | *rulA* |
|  |  |  | 5421354..5422625 | *rulB* |
|  |  |  | 4289410..4292514 c | *dnaE2* |
| *P.fluorescens* SS101 |  | NZ_CM001513.1 | 5487380..5490214 | *uvrA1* |
|  |  |  | 3498188..3500707 c | *uvrA2* |
|  |  |  | 4056483..4058498 c | *uvrB* |
|  |  |  | 2269647..2271470 | *uvrC* |
|  |  |  | 6117689..6119872 | *uvrD* |
|  |  |  | 3191789..3192220 | *rulA* |
|  |  |  | 3192207..3193487 | *rulB* |
|  |  |  | 3502518..3505589 c | *dnaE2* |
| *P.fluorescens* A506 |  | NC_017911.1 | 5279223..5282057 | *uvrA1* |
|  |  |  | 2571306..2573825 | *uvrA2* |
|  |  |  | 3922600..3924615 c | *uvrB* |
|  |  |  | 2329454..2331277 | *uvrC* |
|  |  |  | 5901665..5903842 | *uvrD* |
|  |  |  | 2566424..2569495 | *dnaE2* |
|  | pA506 | CP003042.1 | 2435..2863 | *rulA* |
|  | pA506 |  | 2853..4151 | *rulB* |
| *P.stutzeri* A1501 |  | NC_009434.1 | 908090..910927 c | *uvrA* |
|  |  |  | 3226204..3228219 c | *uvrB* |
|  |  |  | 2267450..2269273 | *uvrC* |
|  |  |  | 201237..203426 | *uvrD* |
|  |  |  | 2564811..2567891 | *dnaE2* |
|  |  | CP000304.1 | 3631391..3631822 c | *rulA* |
| *P.aeruginosa* PAO1 |  | NC_002516.2 | 4748756..4751593 | *uvrA* |
|  |  |  | 3520548..3522560 c | *uvrB* |
|  |  |  | 2923961..2925787 c | *uvrC* |
|  |  |  | 6131088.6133277 | *uvrD* |
|  |  |  | 727608..730703 c | *dnaE2* |
| *P.syringae* HS191 |  | NZ_CP006256.1 | 799241..802075 c | *uvrA* |
|  |  |  | 3789158..3791173 c | *uvrB* |
|  |  |  | 2670602..2672392 | *uvrC* |
|  |  |  | 3129390..3131864 c | *uvrD* |
|  |  |  | 3116909..3120004 c | *dnaE2* |
|  | unnamed | CP006257.1 | 1528..1953 | *rulA* |
|  | unnamed |  | 2048..3229 | *rulB* |
| *P. cichorii* 302959 |  | AY228702.1 | 1..426 | *rulA* |
|  |  |  | 434..1696 | *rulB* |
| *P. fluorescens* Q8r1-96 |  | AHPO01000004.1 | 1328347..1328772 c | *rulA* |
|  |  |  | 1327077..1328354 c | *rulB* |
|  |  |  | 1147007..1150084 c | *dnaE2* |
| *P. fluorescens* Q2-87 |  | AGBM01000001.1 | 2164033..2164458 | *rulA* |
|  |  |  | 2164451..2165728 | *rulB* |
|  |  |  | 3877060..3880137 c | *dnaE2* |
| *P. stutzeri* CGMCC1.1803 |  | CP002881.1 | 3000641..3000970 c | *rulA* |
|  |  |  | 1516938..1520459 | *dnaE2* |
| *P. syringae* A2 | pPSR1 | U43696.3 | 185..610 | *rulA* |
|  | pPSR1 |  | 588..1886 | *rulB* |
| *P. syringae* SM |  | CM001986.1 | 1598122..1598556 | *rulA1* |
|  |  |  | 2223922..2224365 | *rulA2* |
|  |  |  | 1598543..1599823 | *rulB1* |
|  | pSM1 | CM001987.1 | 3221..3646 | *rulA3* |
|  | pSM1 |  | 3660..4922 | *rulB2* |
| *P.syringae* ICMP_9617 |  | AOKP01000181.1 | 5254..5697 c | *rulA* |
|  |  | AOKP01000282.1 | 4340..4588 | *rulB* |
| *Pseudomonas sp.* GLE121 | pGLE121P3 | KC542383.1 | 4603..4965 c | *rulA* |
|  | pGLE121P3 |  | 3342..4619 c | *rulB* |
| *P. putida* PaW1 | pWW0 | AJ344068.1 | 6603..7034 c | *rulA* |
|  | pWW0 |  | 5405..6613 c | *rulB* |
| *E. coli* RW96 | R391 | U13633.3 | 12368..12817 | *rumA/rulA* |
|  | R391 |  | 12825..14093 | *rumB/rulB* |
| *P. putida* G7 | NAH7 | AB237655.1 | 9053..9481 c | *rulA* |
|  | NAH7 |  | 7780..9063 c | *rulB* |
