# Supplementary material for: Contribution of increased mutagenesis to the evolution of pollutants-degrading indigenous bacteria
Source: PLoS One. 2017 Aug 4;12(8):e0182484. doi: 10.1371/journal.pone.0182484 (PMC5544203; doi:10.1371/journal.pone.0182484)
Supplement: S8 Table — The RT-qPCR results were analyzed with Mann-Whitney U test and p-values indicated with red represent statistically significant differences according to Benjamini-Hochberg procedure (FRD = 0.05). (DOCX) [file pone.0182484.s016.docx]

**S8 Table.** **The statistical analysis for distinguishing significant changes in the expression rate of the genes of error-prone DNA polymerases after treatment with MMC.** The RT-qPCR results were analyzed with Mann-Whitney U test and p-values indicated with red represent statistically significant differences according to Benjamini-Hochberg procedure (FRD = 0.05).

| Gene | P-value |
| --- | --- |
| PaW1 *dnaE2/imuC* | <0.0001 |
| PaW1 *rulB_pWW0_* | <0.0001 |
| PC20 *imuC* | 0.0096 |
| PC20 *rulB1* | <0.0001 |
| PC20 *rulB_pG20_* | 0.0061 |
| PC20 *rulB2* | <0.0001 |
| PC24 *rulB1* | <0.0001 |
| PC24 *rulB2* | <0.0001 |
| PC24 *imuC* | <0.0001 |
