# Supplementary material for: Contribution of increased mutagenesis to the evolution of pollutants-degrading indigenous bacteria
Source: PLoS One. 2017 Aug 4;12(8):e0182484. doi: 10.1371/journal.pone.0182484 (PMC5544203; doi:10.1371/journal.pone.0182484)
Supplement: S9 Table — The Mann-Whitney U test was performed to distinguish statistically significant differences of UV-induced and spontaneous Rifr mutant frequencies according to Benjamini-Hochberg procedure (FRD = 0.05). Red indicates the statistically significant differences. The difference between spontaneous and UV-induced mutant frequency is presented as a fold of induction. (DOCX) [file pone.0182484.s017.docx]

**S9 Table.** **The comparison of spontaneous and UV-induced (5 J/m^2^) mutant frequencies.** The Mann-Whitney U test was performed to distinguish statistically significant differences of UV-induced and spontaneous Rif^r^ mutant frequencies according to Benjamini-Hochberg procedure (FRD = 0.05). Red indicates the statistically significant differences. The difference between spontaneous and UV-induced mutant frequency is presented as a fold of induction.

| Strain | UV-induced (5 J/m^2^) | | | | P-value | Fold of induction |
| --- | --- | --- | --- | --- | --- | --- |
|  | Valid N | Median | Lower Quartile | Upper Quartile |  |  |
| PaW85 | 20 | 7.77E-08 | 6.90E-08 | 1.13E-07 | <0.0001 | 4.76 |
| PaWrulAB | 15 | 5.26E-07 | 3.57E-07 | 5.60E-07 | <0.0001 | 61.78 |
| PaW1 | 15 | 2.88E-07 | 2.61E-07 | 3.54E-07 | <0.0001 | 41.81 |
| PC20 | 15 | 5.81E-07 | 4.10E-07 | 8.35E-07 | <0.0001 | 60.38 |
| PC24 | 15 | 6.58E-07 | 4.46E-07 | 1.20E-06 | <0.0001 | 38.09 |
