# Supplementary material for: Contribution of increased mutagenesis to the evolution of pollutants-degrading indigenous bacteria
Source: PLoS One. 2017 Aug 4;12(8):e0182484. doi: 10.1371/journal.pone.0182484 (PMC5544203; doi:10.1371/journal.pone.0182484)
Supplement: S10 Table — Multiple comparisons with Kruskal-Wallis test were performed to distinguish statistically significant differences in the appearance frequency of Rifr mutants in the presence of aromatic compounds. Red indicates the statistically significant differences according to Benjamini-Hochberg procedure (FRD = 0.05). The effect of aromatic substrates to the frequency of Rifr mutants is presented as a fold of induction compared to the cells grown on medium without aromatic compounds. (DOCX) [file pone.0182484.s018.docx]

**S10 Table.** **The effect of aromatic compounds (2.5 mM *m*-cresol or 5 mM phenol with exception of PC20 for which we used 2.5 mM phenol and 1.25 mM *m*-cresol) to the appearance frequency of Rif^r^ mutants in rich medium (M9 medium supplemented with glucose (0.2%) and CAA (0.2%)).** Multiple comparisons with Kruskal-Wallis test were performed to distinguish statistically significant differences in the appearance frequency of Rif^r^ mutants in the presence of aromatic compounds. Red indicates the statistically significant differences according to Benjamini-Hochberg procedure (FRD = 0.05). The effect of aromatic substrates to the frequency of Rif^r^ mutants is presented as a fold of induction compared to the cells grown on medium without aromatic compounds.

| Substrate | Strain | Valid N | Median | Lower Quartile | Upper Quartile | P-value | Fold of induction |
| --- | --- | --- | --- | --- | --- | --- | --- |
| Glc + CAA | PaW85 | 28 | 9.48E-09 | 6.91E-09 | 1.70E-08 |  |  |
| Glc+CAA+Phe | PaW85 | 28 | 6.86E-09 | 2.27E-09 | 1.07E-08 | 0.135 | 0.72 |
| Glc+CAA+m-Cre | PaW85 | 28 | 1.09E-08 | 7.23E-09 | 2.50E-08 | 1.000 | 1.15 |
| Glc + CAA | PaWpheBA25 | 28 | 1.50E-08 | 8.40E-09 | 2.21E-08 |  |  |
| Glc+CAA+Phe | PaWpheBA25 | 29 | 1.46E-08 | 1.02E-08 | 2.21E-08 | 1.000 | 0.97 |
| Glc+CAA+m-Cre | PaWpheBA25 | 29 | 1.32E-08 | 7.30E-09 | 2.63E-08 | 1.000 | 0.88 |
| Glc + CAA | PaWrulAB | 28 | 1.37E-08 | 1.01E-08 | 1.89E-08 |  |  |
| Glc+CAA+Phe | PaWrulAB | 28 | 1.18E-08 | 5.63E-09 | 2.56E-08 | 1.000 | 0.86 |
| Glc+CAA+m-Cre | PaWrulAB | 30 | 9.03E-09 | 3.48E-09 | 2.94E-08 | 0.246 | 0.66 |
| Glc + CAA | PC20 | 28 | 9.43E-09 | 5.05E-09 | 1.42E-08 |  |  |
| Glc+CAA+Phe | PC20 | 29 | 5.06E-09 | 2.84E-09 | 7.39E-09 | 0.047 | 0.54 |
| Glc+CAA+m-Cre | PC20 | 28 | 2.14E-09 | 1.53E-09 | 4.33E-09 | <0.0001 | 0.23 |
| Glc + CAA | PC24 | 29 | 7.73E-09 | 4.77E-09 | 1.13E-08 |  |  |
| Glc+CAA+Phe | PC24 | 30 | 5.31E-09 | 4.52E-09 | 7.57E-09 | 0.293 | 0.69 |
| Glc+CAA+m-Cre | PC24 | 29 | 9.06E-09 | 5.08E-09 | 1.67E-08 | 1.000 | 1.17 |
