# Supplementary material for: Contribution of increased mutagenesis to the evolution of pollutants-degrading indigenous bacteria
Source: PLoS One. 2017 Aug 4;12(8):e0182484. doi: 10.1371/journal.pone.0182484 (PMC5544203; doi:10.1371/journal.pone.0182484)
Supplement: S11 Table — The Mann-Whitney U test was performed to distinguish statistically significant differences in the appearance frequency of Rifr mutants in the presence of different carbon sources. Red indicates the statistically significant results according to Benjamini-Hochberg procedure (FRD = 0.05). The effect of aromatic substrates on the frequency of Rifr mutants is presented as a fold of induction compared to the cells grown on glucose as a sole carbon source. (DOCX) [file pone.0182484.s019.docx]

**S11 Table.** **The frequency of Rif^r^ mutants grown in M9 minimal medium supplemented with either glucose (0.2%), phenol (2.5 mM) or *p*-cresol (1.25 mM) as a sole carbon and energy source.** The Mann-Whitney U test was performed to distinguish statistically significant differences in the appearance frequency of Rif^r^ mutants in the presence of different carbon sources. Red indicates the statistically significant results according to Benjamini-Hochberg procedure (FRD = 0.05). The effect of aromatic substrates on the frequency of Rif^r^ mutants is presented as a fold of induction compared to the cells grown on glucose as a sole carbon source.

| Carbon source | Strain | Valid N | Median | Lower Quartile | Upper Quartile | P-value | Fold of induction |
| --- | --- | --- | --- | --- | --- | --- | --- |
| Glucose | PaWpheBA25 | 19 | 1.24E-08 | 5.00E-09 | 1.72E-08 | <0.0001 | 4.20 |
| Phenol | PaWpheBA25 | 19 | 5.22E-08 | 1.20E-08 | 1.32E-07 |  |  |
| Glucose | PC20 | 20 | 5.67E-08 | 2.50E-08 | 6.98E-08 | 0.024 | 0.37 |
| Phenol | PC20 | 20 | 2.11E-08 | 1.63E-08 | 5.31E-08 |  |  |
| Glucose | PC24 | 20 | 2.25E-08 | 1.15E-08 | 9.73E-08 | 0.583 | 0.72 |
| Phenol | PC24 | 20 | 1.62E-08 | 9.80E-09 | 1.15E-07 |  |  |
| p-Cresol | PC24 | 20 | 1.41E-08 | 7.16E-09 | 4.67E-08 | 0.108 | 0.63 |
