# Supplementary material for: Contribution of increased mutagenesis to the evolution of pollutants-degrading indigenous bacteria
Source: PLoS One. 2017 Aug 4;12(8):e0182484. doi: 10.1371/journal.pone.0182484 (PMC5544203; doi:10.1371/journal.pone.0182484)
Supplement: S12 Table — (DOCX) [file pone.0182484.s020.docx]

**S12 Table. Primers used in the RT-qPCR experiment.**

| Strain | Name | Sequence (5´→ 3´) |
| --- | --- | --- |
| PaW1 | imuC -PaW-Fw | CGA GGA GGT GAT CCA GTA TG |
|  | imuC -PaW-Rev | CCA GCG ACC ACA ACA TTT |
|  | rulB-PaW-Fw | GCC CTG TCG TCG TCG TTC TTT |
|  | rulB-PaW-Rev | ATT CCG CAC CTT GAA GTA GG |
|  | trpA-PaW-Fw | ACG CCT CGT TGC AGA TCC TC |
|  | trpA-PaW-Rev | GTT GTA GTA GCC CAT CAG CAC G |
|  |  |  |
| PC24 | imuC-PC24-Fw | ACT GCC TGT CGA ACT TCA GC |
|  | imuC -PC24-Rev | GCA CCT CAC TGC CGA CGA TC |
|  | rulB1-PC24-Fw | ACT CGA TTG ACG AAA GTT GGC |
|  | rulB1-PC24-Rev | CCA CGA CAC CAC CAG TTT CC |
|  | trpA-PC24-Fw | ACA CCT CCC TGG CGA TCC TC |
|  | trpA-PC24-Rev | GTT GAA GTA GCC CAT CAG CAC C |
|  | rulB2-PC24-Fw | CAT TCG CCG ATT TGA CTG GC |
|  | rulB2-PC24-Rev | TCA CGC TTG GCC GGA TCA G |
|  |  |  |
| PC20 | imuC -PC20-Fw | CAG TGG GAC AAG GAC GAC C |
|  | imuC -PC20-Rev | TGG CTG ATC ATG TCG TAG G |
|  | rulB-pG20-PC20-Fw | GAA CCG CTC AAA GAC GTT GC |
|  | rulB-pG20-PC20-Rev | TAC GCT TCA GGA GCC ACT CC |
|  | rulB1-PC20-Fw | CCG GTG TGC CTG GTG ACT TT |
|  | rulB1-PC20-Rev | CAG ATC AAC GAC AAC TCC GG |
|  | rulB2-PC20-Fw | CTG GAG TGC CAG GTG ATC TC |
|  | rulB2-PC20-Rev | AAG ATC GAC CAC TCC ACC AC |
|  | trpA-PC20-Fw | ACA CGT TTT GCC GAA CTC AAG G |
|  | trpA-PC20-Rev | TTT GCA GGG TTT TCG CCA GG |
|  |  |  |
