# Supplementary material for: Contribution of increased mutagenesis to the evolution of pollutants-degrading indigenous bacteria
Source: PLoS One. 2017 Aug 4;12(8):e0182484. doi: 10.1371/journal.pone.0182484 (PMC5544203; doi:10.1371/journal.pone.0182484)
Supplement: S13 Table — (DOCX) [file pone.0182484.s021.docx]

**S13 Table. Original datasets used to compute statistics.**

The frequency of spontaneous Rif^r^ mutants.

| Strain | Mutant frequency | | Strain | Mutant frequency | | Strain | Mutant frequency |
| --- | --- | --- | --- | --- | --- | --- | --- |
| 2A20 | 1.46E-08 |  | D45 | 1.2E-09 |  | PaW85 | 6.1E-08 |
| 2A20 | 4.53E-08 |  | D45 | 0 |  | PaW85 | 6.21E-08 |
| 2A20 | 7.01E-08 |  | D45 | 3.6E-09 |  | PaW85 | 7.34E-08 |
| 2A20 | 4.96E-08 |  | D45 | 1.59E-08 |  | PaW85 | 2.82E-08 |
| 2A20 | 2.51E-08 |  | D45 | 6.21E-09 |  | PaW85 | 4.18E-08 |
| 2A20 | 3.11E-08 |  | D45 | 8.97E-09 |  | PaW85 | 2.47E-08 |
| 2A20 | 2.51E-08 |  | D45 | 1.34E-08 |  | PaW85 | 1.23E-08 |
| 2A20 | 3.59E-08 |  | D45 | 6.9E-09 |  | PaW85 | 2.47E-08 |
| 2A20 | 5.51E-08 |  | D45 | 4.75E-09 |  | PaW85 | 1.2E-08 |
| 2A20 | 6E-09 |  | D45 | 1.58E-08 |  | PaW85 | 1.2E-08 |
| 2A20 | 2.8E-08 |  | D45 | 1.63E-08 |  | PaW85 | 4.67E-09 |
| 2A20 | 3.6E-08 |  | D45 | 2.45E-08 |  | PaW85 | 3.58E-08 |
| 2A20 | 8.8E-08 |  | D45 | 5.75E-09 |  | PaW85 | 4.44E-09 |
| 2A20 | 6.09E-08 |  | D66v | 2.93E-10 |  | PaW85 | 1.53E-08 |
| 2A20 | 2.7E-08 |  | D66v | 5.86E-10 |  | PaW85 | 1.11E-08 |
| 2A20 | 2.7E-08 |  | D66v | 2.08E-08 |  | PaW85 | 7.94E-09 |
| 2A20 | 1.13E-08 |  | D66v | 2.93E-10 |  | PaW85 | 7.62E-09 |
| 2A20 | 2.43E-08 |  | D66v | 3.08E-09 |  | PaW85 | 1.02E-08 |
| 2A20 | 1.91E-08 |  | D66v | 0 |  | PaW85 | 4.44E-08 |
| 2A20 | 4.61E-08 |  | D66v | 1.38E-08 |  | PaWrulAB | 8.83E-09 |
| 2A20 | 1.48E-08 |  | D66v | 1.54E-09 |  | PaWrulAB | 4.94E-09 |
| 2A20 | 7.82E-08 |  | D66v | 8.62E-09 |  | PaWrulAB | 1.82E-08 |
| 2A20 | 7.09E-08 |  | D66v | 2.41E-08 |  | PaWrulAB | 5.45E-09 |
| 2A20 | 8.18E-08 |  | D66v | 0 |  | PaWrulAB | 1.56E-09 |
| 2A20 | 8.73E-08 |  | D66v | 2.24E-08 |  | PaWrulAB | 7.4E-09 |
| 2A20 | 6.91E-08 |  | D66v | 4.8E-09 |  | PaWrulAB | 1.38E-08 |
| 2A20 | 1.5E-07 |  | D66v | 7.2E-09 |  | PaWrulAB | 3.8E-09 |
| 2A20 | 7E-08 |  | D66v | 8E-10 |  | PaWrulAB | 5.4E-09 |
| 2A20 | 1.24E-07 |  | D66v | 8E-10 |  | PaWrulAB | 8.2E-09 |
| 2A20 | 7E-08 |  | D66v | 6.4E-09 |  | PaWrulAB | 1.3E-08 |
| 2A20 | 3.6E-08 |  | D66v | 1.33E-08 |  | PaWrulAB | 1.78E-08 |
| 2A20 | 2E-08 |  | D66v | 7.78E-09 |  | PaWrulAB | 2.13E-08 |
| 2A20 | 1.07E-08 |  | D66v | 6.11E-08 |  | PaWrulAB | 3.3E-08 |
| 2A20 | 2.8E-08 |  | D66v | 8.89E-09 |  | PC13 | 1.03E-08 |
| 2A38 | 6.57E-08 |  | D66v | 8.89E-09 |  | PC13 | 1.43E-09 |
| 2A38 | 1.17E-07 |  | D66v | 3.75E-09 |  | PC13 | 2.51E-08 |
| 2A38 | 1.6E-07 |  | D66v | 3.75E-09 |  | PC13 | 8.57E-09 |
| 2A38 | 5.71E-09 |  | D66v | 4.38E-08 |  | PC13 | 8.86E-09 |
| 2A38 | 2.3E-09 |  | D66v | 5E-09 |  | PC13 | 4.66E-09 |
| 2A38 | 6.44E-08 |  | D66v | 5E-09 |  | PC13 | 1.84E-08 |
| 2A38 | 9.2E-08 |  | D66v | 3.64E-09 |  | PC13 | 6.3E-09 |
| 2A38 | 2.3E-09 |  | D66v | 1.27E-08 |  | PC13 | 4.93E-09 |
| 2A38 | 0 |  | D66v | 2.91E-08 |  | PC13 | 6.58E-09 |
| 2A38 | 6.35E-08 |  | D66v | 5E-09 |  | PC13 | 4.19E-09 |
| 2A38 | 1.13E-08 |  | D66v | 3.75E-09 |  | PC13 | 3.26E-08 |
| 2A38 | 4.26E-08 |  | D66v | 8.75E-08 |  | PC13 | 2.19E-08 |
| 2A54 | 3.45E-08 |  | D66v | 5E-09 |  | PC13 | 1.07E-08 |
| 2A54 | 3.21E-08 |  | D66v | 0 |  | PC13 | 6.57E-09 |
| 2A54 | 2.17E-08 |  | D67 | 1.35E-08 |  | PC13 | 2.99E-09 |
| 2A54 | 2.62E-08 |  | D67 | 1.14E-08 |  | PC13 | 1.37E-08 |
| 2A54 | 2.21E-08 |  | D67 | 1.18E-08 |  | PC13 | 3.58E-09 |
| 2A54 | 4.75E-08 |  | D67 | 6.12E-09 |  | PC13 | 8.96E-09 |
| 2A54 | 3.82E-08 |  | D67 | 7.17E-09 |  | PC13 | 8.68E-09 |
| 2A54 | 2.48E-08 |  | D67 | 4.15E-09 |  | PC13 | 6.04E-09 |
| 2A54 | 1.37E-08 |  | D67 | 6.79E-09 |  | PC13 | 2.04E-08 |
| 2A54 | 2.38E-08 |  | D67 | 1.28E-08 |  | PC13 | 1.62E-08 |
| 2A54 | 3.09E-08 |  | D67 | 8.57E-09 |  | PC13 | 9.81E-09 |
| 2A54 | 2.72E-08 |  | D67 | 1.11E-08 |  | PC14 | 3.71E-08 |
| 2A54 | 2.94E-08 |  | D67 | 1.2E-08 |  | PC14 | 2.48E-08 |
| 2Anah4 | 2.19E-08 |  | D67 | 5.71E-09 |  | PC14 | 2.02E-08 |
| 2Anah4 | 3E-08 |  | D67 | 1.83E-08 |  | PC14 | 1.61E-08 |
| 2Anah4 | 1.94E-08 |  | Hd1 | 4.66E-09 |  | PC14 | 1.98E-08 |
| 2Anah4 | 1.75E-08 |  | Hd1 | 1.95E-08 |  | PC14 | 8.18E-09 |
| 2Anah4 | 6.09E-08 |  | Hd1 | 3.29E-09 |  | PC14 | 1.33E-08 |
| 2Anah4 | 6.45E-08 |  | Hd1 | 1.34E-08 |  | PC14 | 1.29E-08 |
| 2Anah4 | 6.09E-08 |  | Hd1 | 6.03E-09 |  | PC14 | 2.98E-08 |
| 2Anah4 | 3.73E-08 |  | Hd1 | 1.01E-08 |  | PC14 | 9.82E-09 |
| 2Anah4 | 4.73E-08 |  | Hd1 | 2.36E-08 |  | PC14 | 3.35E-08 |
| 2Anah4 | 5.91E-08 |  | Hd1 | 3.56E-09 |  | PC14 | 2.12E-08 |
| 2Anah4 | 6.36E-09 |  | Hd1 | 1.04E-08 |  | PC14 | 6.17E-09 |
| 2Anah4 | 4.55E-08 |  | Hd1 | 2.76E-09 |  | PC14 | 9.17E-09 |
| 2Anah4 | 4.09E-08 |  | Hd1 | 1.72E-09 |  | PC14 | 2.2E-08 |
| 2B45 | 2.52E-07 |  | Hd1 | 4.66E-09 |  | PC14 | 1.3E-08 |
| 2B45 | 1.74E-07 |  | Hd1 | 4.66E-09 |  | PC14 | 1.32E-08 |
| 2B45 | 5.39E-07 |  | Hd1 | 2.59E-09 |  | PC14 | 8.62E-09 |
| 2B45 | 3.91E-07 |  | Hd16 | 4.22E-09 |  | PC14 | 1.4E-08 |
| 2B45 | 3.65E-07 |  | Hd16 | 7.11E-09 |  | PC14 | 3.27E-08 |
| 2B45 | 5.38E-07 |  | Hd16 | 6.67E-09 |  | PC14 | 1.44E-08 |
| 2B45 | 1.54E-08 |  | Hd16 | 2.89E-09 |  | PC14 | 2E-08 |
| 2B45 | 3.38E-07 |  | Hd16 | 3.57E-09 |  | PC14 | 8.67E-09 |
| 2B45 | 6E-08 |  | Hd16 | 2.86E-09 |  | PC14 | 1.51E-08 |
| 2B45 | 0 |  | Hd16 | 8.21E-09 |  | PC15 | 7.96E-09 |
| 2B45 | 1E-07 |  | Hd16 | 7.86E-09 |  | PC15 | 1.51E-08 |
| 2B45 | 7E-07 |  | Hd16 | 2E-09 |  | PC15 | 8.35E-09 |
| 2B45 | 4E-08 |  | Hd16 | 4.22E-09 |  | PC15 | 5.83E-09 |
| 2C23 | 4.37E-07 |  | Hd16 | 4.22E-09 |  | PC15 | 1.44E-08 |
| 2C23 | 4.26E-07 |  | Hd16 | 7.11E-09 |  | PC15 | 1.87E-08 |
| 2C23 | 2.51E-07 |  | Hd16 | 7.56E-09 |  | PC15 | 1.49E-08 |
| 2C23 | 1.58E-07 |  | Hd6 | 4.27E-09 |  | PC15 | 7.33E-09 |
| 2C23 | 3.63E-07 |  | Hd6 | 6.4E-09 |  | PC15 | 1.89E-08 |
| 2C23 | 1.55E-07 |  | Hd6 | 7.47E-09 |  | PC15 | 1.73E-08 |
| 2C23 | 3.62E-07 |  | Hd6 | 5.6E-09 |  | PC15 | 2.76E-08 |
| 2C23 | 2.63E-07 |  | Hd6 | 3.47E-09 |  | PC15 | 2.25E-08 |
| 2C23 | 1.7E-07 |  | Hd6 | 0 |  | PC15 | 1.94E-08 |
| 2C23 | 1.98E-07 |  | Hd6 | 9.56E-09 |  | PC15 | 3.33E-08 |
| 2C23 | 2.49E-07 |  | Hd6 | 1.11E-09 |  | PC16 | 2.29E-09 |
| 2C23 | 1.52E-07 |  | Hd6 | 1.04E-08 |  | PC16 | 7.14E-09 |
| 2C23 | 3.32E-07 |  | Hd6 | 1.56E-09 |  | PC16 | 8.57E-09 |
| 2C41 | 3.06E-08 |  | Hd6 | 3.72E-09 |  | PC16 | 3.14E-09 |
| 2C41 | 1.43E-07 |  | Hd6 | 2.09E-09 |  | PC16 | 1.26E-08 |
| 2C41 | 2.43E-08 |  | Hd6 | 3.26E-09 |  | PC16 | 1.8E-08 |
| 2C41 | 5.57E-08 |  | Hd6 | 1.23E-08 |  | PC16 | 9E-09 |
| 2C41 | 3.15E-08 |  | Hd6 | 8.37E-09 |  | PC16 | 3E-09 |
| 2C41 | 8.43E-08 |  | Hp2 | 9.28E-09 |  | PC16 | 1.33E-09 |
| 2C41 | 1.46E-07 |  | Hp2 | 3.13E-08 |  | PC16 | 1.33E-09 |
| 2C41 | 1.07E-07 |  | Hp2 | 1.22E-08 |  | PC16 | 9.85E-10 |
| 2C41 | 1.34E-07 |  | Hp2 | 2.89E-09 |  | PC16 | 2.56E-08 |
| 2C41 | 5E-08 |  | Hp2 | 1.63E-08 |  | PC16 | 1.28E-08 |
| 2C41 | 2.6E-08 |  | Hp2 | 9.46E-09 |  | PC16 | 1.97E-09 |
| 2C41 | 8.8E-08 |  | Hp2 | 4.73E-09 |  | PC16 | 4.44E-09 |
| 2C41 | 4E-08 |  | Hp2 | 3.23E-09 |  | PC16 | 6.67E-09 |
| 2C41 | 2.8E-08 |  | Hp2 | 1.23E-08 |  | PC16 | 1.44E-08 |
| 2C56 | 2.63E-08 |  | Hp2 | 4.3E-09 |  | PC16 | 1.56E-08 |
| 2C56 | 0 |  | Hp2 | 1.13E-08 |  | PC16 | 2.35E-08 |
| 2C56 | 1.25E-09 |  | Hp2 | 3.08E-08 |  | PC16 | 2.48E-08 |
| 2C56 | 1.25E-09 |  | Hp2 | 2.03E-08 |  | PC16 | 1.05E-08 |
| 2C56 | 2.5E-09 |  | Hp2 | 8.42E-09 |  | PC16 | 3.72E-09 |
| 2C56 | 0 |  | Hp2 | 3.68E-09 |  | PC16 | 5.57E-09 |
| 2C56 | 3.08E-09 |  | Hp5 | 1.42E-08 |  | PC16 | 2E-09 |
| 2C56 | 4.62E-09 |  | Hp5 | 1.51E-08 |  | PC16 | 5.2E-09 |
| 2C56 | 3E-08 |  | Hp5 | 1.12E-08 |  | PC16 | 4E-09 |
| 2C56 | 1.25E-09 |  | Hp5 | 1.32E-08 |  | PC16 | 6E-09 |
| 2C63 | 4.94E-08 |  | Hp5 | 3.7E-08 |  | PC16 | 4E-10 |
| 2C63 | 2.71E-08 |  | Hp5 | 9.89E-09 |  | PC16 | 3.02E-09 |
| 2C63 | 2.76E-08 |  | Hp5 | 1.96E-08 |  | PC16 | 8.3E-09 |
| 2C63 | 8.76E-08 |  | Hp5 | 9.89E-09 |  | PC16 | 3.77E-09 |
| 2C63 | 8.82E-09 |  | Hp5 | 1.74E-08 |  | PC16 | 6.04E-09 |
| 2C63 | 2E-07 |  | Hp5 | 1.76E-08 |  | PC16 | 4.91E-09 |
| 2C63 | 1E-07 |  | Hp5 | 2.42E-08 |  | PC16 | 7.5E-09 |
| 2C63 | 9.26E-08 |  | Hp5 | 1.19E-08 |  | PC16 | 1.5E-09 |
| 2C63 | 1.74E-07 |  | Hp5 | 1.61E-08 |  | PC16 | 4.5E-09 |
| 2C63 | 3E-08 |  | Hp5 | 3.16E-08 |  | PC16 | 6E-09 |
| 2C63 | 5.8E-08 |  | Hp6 | 1.43E-08 |  | PC16 | 1.03E-08 |
| 2C63 | 3E-08 |  | Hp6 | 7.67E-09 |  | PC16 | 1.4E-08 |
| 2C63 | 9E-09 |  | Hp6 | 8.83E-09 |  | PC16 | 2.69E-09 |
| 2C63 | 2.8E-08 |  | Hp6 | 2.93E-09 |  | PC16 | 1.25E-08 |
| 2D47 | 4.19E-09 |  | Hp6 | 2.11E-09 |  | PC16 | 2.39E-09 |
| 2D47 | 2.37E-08 |  | Hp6 | 3.41E-09 |  | PC16 | 4.78E-09 |
| 2D47 | 1.3E-08 |  | Hp6 | 2.28E-09 |  | PC16 | 4.16E-09 |
| 2D47 | 1.21E-08 |  | Hp6 | 6.18E-09 |  | PC16 | 4.94E-09 |
| 2D47 | 1.16E-08 |  | Hp6 | 6E-09 |  | PC16 | 7.79E-09 |
| 2D47 | 1.16E-08 |  | Hp6 | 8E-09 |  | PC16 | 1.87E-08 |
| 2D47 | 1.87E-08 |  | Hp6 | 1.18E-08 |  | PC16 | 2.08E-09 |
| 2D47 | 5.16E-09 |  | Hp6 | 4.25E-09 |  | PC17 | 2E-08 |
| 2D47 | 1.23E-08 |  | Hp6 | 8.5E-09 |  | PC17 | 4.91E-09 |
| 2D47 | 3.23E-09 |  | Nah4 | 7.57E-09 |  | PC17 | 1.4E-08 |
| 2D47 | 1.31E-08 |  | Nah4 | 4.86E-09 |  | PC17 | 1.21E-08 |
| 2D47 | 7.93E-09 |  | Nah4 | 4.86E-09 |  | PC17 | 1.13E-08 |
| 2D47 | 4.14E-09 |  | Nah4 | 1.08E-09 |  | PC17 | 5.58E-09 |
| 2D47 | 5.17E-09 |  | Nah4 | 8.57E-09 |  | PC17 | 1.95E-08 |
| 2D47 | 5.86E-09 |  | Nah4 | 1.27E-09 |  | PC17 | 1.26E-08 |
| 2D61 | 2.59E-08 |  | Nah4 | 9.52E-10 |  | PC17 | 1.4E-09 |
| 2D61 | 1.35E-07 |  | Nah4 | 2.46E-09 |  | PC17 | 1.95E-08 |
| 2D61 | 8.59E-08 |  | Nah4 | 3.51E-10 |  | PC17 | 1.65E-08 |
| 2D61 | 5.19E-08 |  | Nah4 | 2.81E-09 |  | PC17 | 2.5E-09 |
| 2D61 | 2.03E-07 |  | Nah4 | 8.42E-09 |  | PC17 | 5E-09 |
| 2D61 | 3.75E-08 |  | P3 | 4.26E-09 |  | PC18 | 1E-08 |
| 2D61 | 3.19E-08 |  | P3 | 1.15E-08 |  | PC18 | 8E-10 |
| 2D61 | 4.19E-08 |  | P3 | 5.96E-09 |  | PC18 | 1E-08 |
| 2D61 | 4.19E-08 |  | P3 | 1.7E-09 |  | PC18 | 4E-09 |
| 2D61 | 1.09E-07 |  | P3 | 1.19E-08 |  | PC18 | 3.53E-08 |
| 2D61 | 1.43E-07 |  | P3 | 3E-09 |  | PC18 | 1.18E-08 |
| 2D61 | 7.17E-08 |  | P3 | 2.43E-08 |  | PC18 | 2.24E-08 |
| 2D61 | 2.23E-07 |  | P3 | 4.67E-09 |  | PC18 | 1.15E-07 |
| 2D61 | 1.02E-07 |  | P3 | 4E-09 |  | PC18 | 8.58E-10 |
| 2D61 | 8.68E-08 |  | P3 | 3.4E-08 |  | PC18 | 4.64E-08 |
| 2D61 | 2.04E-07 |  | P3 | 1.26E-08 |  | PC18 | 8.58E-10 |
| 2D61 | 2.7E-07 |  | P3 | 3.72E-09 |  | PC18 | 5.49E-08 |
| 2D61 | 1.47E-07 |  | P3 | 2.23E-08 |  | PC18 | 6.87E-09 |
| 2D61 | 1.26E-07 |  | P3 | 4.65E-09 |  | PC18 | 3.7E-08 |
| 2D61 | 2.13E-07 |  | P3 | 1.07E-08 |  | PC18 | 4.26E-10 |
| 2D61 | 2.37E-07 |  | P37 | 1.19E-08 |  | PC18 | 3.57E-08 |
| 2D61 | 1.07E-07 |  | P37 | 1.06E-08 |  | PC18 | 2.55E-09 |
| 2D61 | 1.26E-07 |  | P37 | 7.53E-09 |  | PC18 | 7.66E-09 |
| 2D61 | 1.6E-07 |  | P37 | 3.9E-09 |  | PC18 | 1.82E-09 |
| 2D61 | 9.36E-08 |  | P37 | 7.53E-09 |  | PC18 | 4.94E-09 |
| 2D61 | 1.98E-07 |  | P37 | 2.6E-10 |  | PC18 | 1.66E-08 |
| 2D61 | 8.14E-08 |  | P37 | 4.68E-09 |  | PC18 | 2.34E-08 |
| 2D61 | 2.71E-07 |  | P37 | 3.9E-09 |  | PC18 | 1.01E-08 |
| 2D61 | 8.36E-08 |  | P37 | 2.6E-09 |  | PC18 | 4.15E-09 |
| 2D61 | 7.72E-08 |  | P37 | 4.68E-09 |  | PC18 | 3.02E-09 |
| 2D61 | 9.63E-08 |  | P37 | 1.49E-08 |  | PC18 | 6.42E-09 |
| 2D61 | 1.35E-07 |  | P37 | 8.94E-09 |  | PC18 | 1.17E-08 |
| 2D61 | 4.84E-08 |  | P37 | 1.49E-08 |  | PC18 | 6.04E-09 |
| 2D61 | 6.74E-08 |  | P4 | 8.7E-10 |  | PC18 | 2.6E-09 |
| 2D61 | 1.01E-07 |  | P4 | 8.26E-09 |  | PC18 | 2.6E-09 |
| 2D61 | 6.71E-08 |  | P4 | 2.17E-09 |  | PC18 | 9.8E-09 |
| 2D61 | 1.23E-07 |  | P4 | 1.74E-09 |  | PC18 | 2E-09 |
| 2D61 | 6.65E-08 |  | P4 | 1.06E-08 |  | PC18 | 9.8E-09 |
| 2D61 | 6.39E-08 |  | P4 | 8.43E-09 |  | PC18 | 1.33E-09 |
| 2D61 | 6.8E-08 |  | P4 | 3.61E-09 |  | PC18 | 5.11E-09 |
| 2D61 | 2.94E-07 |  | P4 | 9.52E-10 |  | PC18 | 7.11E-09 |
| 2D61 | 1.33E-07 |  | P4 | 2.22E-09 |  | PC18 | 1.56E-09 |
| 2D61 | 7.52E-08 |  | P4 | 9.52E-10 |  | PC18 | 4.22E-09 |
| 2D61 | 2.87E-08 |  | P4 | 3.49E-09 |  | PC20 | 2.19E-09 |
| 2D61 | 1.51E-07 |  | P4 | 6.35E-10 |  | PC20 | 9.32E-09 |
| 2D61 | 3.28E-08 |  | P48 | 1.67E-08 |  | PC20 | 3.29E-09 |
| 2D61 | 4.72E-08 |  | P48 | 6.67E-09 |  | PC20 | 1.67E-08 |
| 2D61 | 6.34E-08 |  | P48 | 9.67E-09 |  | PC20 | 2.47E-08 |
| 2D66 | 6.46E-08 |  | P48 | 1.9E-08 |  | PC20 | 4.26E-09 |
| 2D66 | 4E-08 |  | P48 | 8.67E-09 |  | PC20 | 4.68E-09 |
| 2D66 | 1.26E-08 |  | P48 | 5.67E-09 |  | PC20 | 3.4E-09 |
| 2D66 | 1.13E-07 |  | P48 | 8.67E-09 |  | PC20 | 5.53E-09 |
| 2D66 | 1.31E-08 |  | P48 | 1.7E-08 |  | PC20 | 0 |
| 2D66 | 6.53E-08 |  | P48 | 2E-08 |  | PC20 | 7.84E-09 |
| 2D66 | 4.74E-08 |  | P48 | 1.2E-08 |  | PC20 | 8.5E-09 |
| 2D66 | 4.53E-08 |  | P48 | 1.4E-08 |  | PC20 | 1.31E-08 |
| 2D66 | 3.47E-08 |  | P48 | 9E-09 |  | PC20 | 5.23E-09 |
| 2D66 | 1.41E-07 |  | P48 | 4.67E-09 |  | PC20 | 3.11E-09 |
| 2D66 | 5E-08 |  | P49 | 4.89E-09 |  | PC20 | 1.04E-09 |
| 2D66 | 4.93E-08 |  | P49 | 1.29E-08 |  | PC20 | 8.29E-09 |
| 2D66 | 2.43E-08 |  | P49 | 3.11E-09 |  | PC20 | 0 |
| 2D66 | 6.07E-08 |  | P49 | 8.67E-09 |  | PC20 | 9.33E-09 |
| 2D67 | 6.3E-09 |  | P49 | 1.02E-08 |  | PC20 | 1.07E-09 |
| 2D67 | 0 |  | P49 | 3.64E-09 |  | PC20 | 1.07E-09 |
| 2D67 | 0 |  | P49 | 4.36E-09 |  | PC20 | 2.14E-09 |
| 2D67 | 0 |  | P49 | 9.27E-09 |  | PC20 | 9.63E-09 |
| 2D67 | 1.57E-09 |  | P49 | 6.91E-09 |  | PC20 | 1.5E-08 |
| 2D67 | 5.68E-09 |  | P49 | 1.09E-08 |  | PC20 | 4.1E-08 |
| 2D67 | 7.95E-09 |  | P49 | 2.89E-09 |  | PC20 | 1E-08 |
| 2D67 | 2.27E-09 |  | P49 | 3.11E-09 |  | PC20 | 3.9E-08 |
| 2D67 | 2.27E-09 |  | P49 | 2.67E-09 |  | PC20 | 6E-09 |
| 2D67 | 4.55E-09 |  | P6 | 1.04E-08 |  | PC20 | 0 |
| 2D67 | 8.57E-09 |  | P6 | 4.66E-09 |  | PC20 | 2.13E-08 |
| 2D67 | 1.9E-09 |  | P6 | 6.85E-09 |  | PC20 | 6.23E-09 |
| 2D67 | 1.9E-09 |  | P6 | 4.93E-09 |  | PC20 | 1.79E-08 |
| 2D67 | 1.24E-08 |  | P6 | 1.16E-08 |  | PC20 | 1.9E-08 |
| 2D67 | 2.86E-09 |  | P6 | 2.16E-08 |  | PC20 | 4.16E-09 |
| 2D67 | 2.86E-09 |  | P6 | 1.07E-08 |  | PC20 | 7.33E-08 |
| 2D67 | 0 |  | P6 | 7.33E-09 |  | PC20 | 4.38E-08 |
| 2D67 | 1.37E-08 |  | P6 | 5.11E-09 |  | PC20 | 4.19E-08 |
| 2D67 | 5.26E-09 |  | P6 | 4.33E-09 |  | PC20 | 7.43E-08 |
| 2D67 | 4.21E-09 |  | P6 | 3.67E-09 |  | PC20 | 5.24E-08 |
| 2D67 | 1.05E-09 |  | P6 | 5.67E-09 |  | PC20 | 7.32E-08 |
| 2D67 | 1.05E-09 |  | P6 | 2.73E-08 |  | PC20 | 3.03E-08 |
| 2D67 | 0 |  | P69 | 1.62E-09 |  | PC20 | 4.42E-08 |
| 2D67 | 9.23E-09 |  | P69 | 6.49E-09 |  | PC20 | 4.67E-08 |
| 2D67 | 0 |  | P69 | 2.7E-09 |  | PC20 | 4.23E-08 |
| 2D67 | 1.54E-09 |  | P69 | 2E-09 |  | PC20 | 1.46E-08 |
| 2D67 | 0 |  | P69 | 6.67E-09 |  | PC20 | 6.49E-09 |
| 2D67 | 5.71E-09 |  | P69 | 2E-09 |  | PC20 | 2.97E-08 |
| 2D67 | 0 |  | P69 | 6E-09 |  | PC20 | 4.81E-08 |
| 2D67 | 8.57E-09 |  | P69 | 6.67E-09 |  | PC20 | 2.7E-08 |
| 2D67 | 5.71E-09 |  | P69 | 3.02E-09 |  | PC24 | 1.25E-08 |
| 2D67 | 4.29E-09 |  | P69 | 6.04E-09 |  | PC24 | 8.73E-09 |
| C52 | 1.28E-07 |  | P69 | 3.77E-10 |  | PC24 | 2.49E-08 |
| C52 | 2.67E-08 |  | P69 | 2.26E-09 |  | PC24 | 2.16E-08 |
| C52 | 1.05E-07 |  | P69 | 4.15E-09 |  | PC24 | 4E-09 |
| C52 | 6.48E-08 |  | P85 | 1.29E-08 |  | PC24 | 1.11E-08 |
| C52 | 8.95E-08 |  | P85 | 2.2E-08 |  | PC24 | 3.42E-08 |
| C52 | 9.45E-08 |  | P85 | 1.57E-08 |  | PC24 | 7.08E-09 |
| C52 | 1.1E-07 |  | P85 | 1.4E-08 |  | PC24 | 4.15E-09 |
| C52 | 1.03E-07 |  | P85 | 9.11E-09 |  | PC24 | 2.62E-09 |
| C52 | 6.99E-08 |  | P85 | 2.02E-08 |  | PC24 | 1.04E-08 |
| C52 | 9.45E-08 |  | P85 | 2.18E-08 |  | PC24 | 3.46E-08 |
| C52 | 1.11E-07 |  | P85 | 4.2E-08 |  | PC24 | 9.52E-09 |
| C52 | 1.2E-07 |  | P85 | 7.74E-09 |  | PC24 | 6.42E-09 |
| C52 | 5.18E-08 |  | P85 | 1.57E-08 |  | PC24 | 9.52E-09 |
| C70 | 0 |  | P85 | 1.51E-08 |  | PC24 | 2.22E-08 |
| C70 | 5.41E-10 |  | P85 | 1.42E-08 |  | PC24 | 2.76E-08 |
| C70 | 1.08E-09 |  | P85 | 1.98E-08 |  | PC24 | 4.32E-08 |
| C70 | 1.62E-09 |  | P86 | 3.72E-09 |  | PC24 | 4.68E-08 |
| C70 | 1.62E-09 |  | P86 | 1.86E-09 |  | PC24 | 4.08E-08 |
| C70 | 5E-09 |  | P86 | 9.3E-10 |  | PC24 | 1.08E-08 |
| C70 | 6.43E-09 |  | P86 | 9.3E-10 |  | PC24 | 1.54E-08 |
| C70 | 2.14E-09 |  | P86 | 3.68E-09 |  | PC24 | 1.42E-08 |
| C70 | 0 |  | P86 | 2.53E-09 |  | PC24 | 1.61E-08 |
| C70 | 2.5E-09 |  | P86 | 2.3E-10 |  | PC24 | 2.14E-08 |
| C70 | 1.45E-08 |  | P86 | 5.29E-09 |  | PC24 | 3.78E-08 |
| C70 | 9.5E-09 |  | P86 | 1.38E-09 |  | PC24 | 8.49E-09 |
| C70 | 1E-09 |  | P86 | 2E-09 |  | PC24 | 8.22E-09 |
| D113 | 1.03E-07 |  | P86 | 5.25E-09 |  | PC24 | 1.84E-08 |
| D113 | 4.91E-08 |  | P86 | 7.75E-09 |  | PC24 | 5.05E-09 |
| D113 | 3.34E-08 |  | P86 | 4.5E-09 |  | PC24 | 9.35E-09 |
| D113 | 8.29E-08 |  | P86 | 2E-09 |  | PC24 | 1.2E-08 |
| D113 | 7.71E-08 |  | P94 | 1.89E-09 |  | PC24 | 1.35E-08 |
| D113 | 3.79E-08 |  | P94 | 5.66E-09 |  | PC24 | 4.11E-09 |
| D113 | 1.94E-08 |  | P94 | 3.4E-09 |  | PC24 | 2.95E-08 |
| D113 | 3.9E-08 |  | P94 | 3.02E-09 |  | PC24 | 1.73E-08 |
| D113 | 2.99E-08 |  | P94 | 0 |  | PC24 | 3.95E-08 |
| D113 | 5.51E-08 |  | P94 | 1E-09 |  | PC24 | 3E-08 |
| D113 | 4.27E-08 |  | P94 | 3.67E-09 |  | PC24 | 2.82E-08 |
| D113 | 2.04E-08 |  | P94 | 2E-09 |  | PC24 | 2.28E-08 |
| D113 | 2.8E-08 |  | P94 | 2.67E-09 |  | PC24 | 1.48E-08 |
| D113 | 3.27E-08 |  | P94 | 4E-09 |  | PC24 | 1.97E-08 |
| D113 | 2.47E-08 |  | P94 | 1.02E-08 |  | PC24 | 3.66E-08 |
| D14 | 0 |  | P94 | 9.77E-09 |  | PC24 | 4.54E-08 |
| D14 | 2.8E-09 |  | P94 | 1.02E-08 |  | PC24 | 1.77E-08 |
| D14 | 0 |  | P94 | 3.72E-09 |  | PC24 | 2.91E-08 |
| D14 | 0 |  | P94 | 6.51E-09 |  | PC24 | 1.29E-08 |
| D14 | 2.35E-09 |  | PaW1 | 2.54E-09 |  | PC24 | 3.4E-08 |
| D14 | 0 |  | PaW1 | 1.59E-08 |  | PC24 | 1.97E-08 |
| D14 | 2.72E-09 |  | PaW1 | 1.43E-08 |  | PC30 | 1E-08 |
| D14 | 0 |  | PaW1 | 6.35E-09 |  | PC30 | 2.47E-08 |
| D14 | 1.36E-09 |  | PaW1 | 4.58E-09 |  | PC30 | 4.57E-09 |
| D14 | 0 |  | PaW1 | 1.47E-08 |  | PC30 | 1.6E-08 |
| D14 | 5.22E-09 |  | PaW1 | 9.16E-09 |  | PC30 | 1.5E-08 |
| D14 | 2.61E-09 |  | PaW1 | 9.64E-09 |  | PC30 | 1.42E-08 |
| D14 | 5.22E-09 |  | PaW1 | 4.58E-09 |  | PC30 | 1.13E-08 |
| D14 | 2.61E-09 |  | PaW1 | 5.71E-09 |  | PC30 | 2.1E-08 |
| D14 | 0 |  | PaW1 | 8.57E-10 |  | PC30 | 1.13E-08 |
| D14 | 6.15E-09 |  | PaW1 | 7.43E-09 |  | PC30 | 7.23E-09 |
| D14 | 4.62E-09 |  | PaW1 | 4E-09 |  | PC30 | 2.38E-08 |
| D14 | 1.54E-09 |  | PaW1 | 1.03E-08 |  | PC30 | 3.76E-08 |
| D14 | 4.29E-09 |  | PaW85 | 2.19E-08 |  | PC30 | 1.96E-08 |
| D14 | 7.14E-09 |  | PaW85 | 3.56E-08 |  | PC34 | 3.87E-09 |
| D14 | 1.43E-09 |  | PaW85 | 1.63E-08 |  | PC34 | 1.51E-09 |
| D14 | 4.29E-09 |  | PaW85 | 1.44E-08 |  | PC34 | 1.72E-09 |
| D14 | 2.86E-09 |  | PaW85 | 4.13E-08 |  | PC34 | 3.87E-09 |
| D14 | 3.64E-09 |  | PaW85 | 2.29E-08 |  | PC34 | 8.05E-09 |
| D14 | 1.82E-09 |  | PaW85 | 5.64E-08 |  | PC34 | 5.06E-09 |
| D14 | 7.27E-09 |  | PaW85 | 7.5E-08 |  | PC34 | 7.82E-09 |
| D14 | 5.45E-09 |  | PaW85 | 5.57E-08 |  | PC34 | 1.24E-08 |
| D14 | 2.22E-09 |  | PaW85 | 1.64E-08 |  | PC34 | 1.36E-08 |
| D14 | 4.44E-09 |  | PaW85 | 9.55E-09 |  | PC34 | 4.91E-09 |
| D14 | 2.22E-09 |  | PaW85 | 1.73E-08 |  | PC34 | 8.91E-09 |
| D14 | 0 |  | PaW85 | 2.15E-08 |  | PC34 | 1.89E-08 |
| D14 | 0 |  | PaW85 | 2.33E-08 |  | PC34 | 1.95E-08 |
| D14 | 1.43E-09 |  | PaW85 | 2.71E-08 |  | PC34 | 4E-09 |
| D14 | 0 |  | PaW85 | 3.86E-08 |  | PC36 | 3.87E-09 |
| D14 | 2.86E-09 |  | PaW85 | 6E-08 |  | PC36 | 2.97E-08 |
| D28 | 2.15E-08 |  | PaW85 | 8.14E-08 |  | PC36 | 1.08E-08 |
| D28 | 1.07E-08 |  | PaW85 | 7.5E-08 |  | PC36 | 9.25E-09 |
| D28 | 1.12E-08 |  | PaW85 | 1.79E-08 |  | PC36 | 1.1E-08 |
| D28 | 5.71E-08 |  | PaW85 | 2E-08 |  | PC36 | 1.35E-08 |
| D28 | 1.9E-08 |  | PaW85 | 5.86E-08 |  | PC36 | 8.54E-09 |
| D28 | 3.96E-08 |  | PaW85 | 7.4E-09 |  | PC36 | 6.67E-09 |
| D28 | 1.02E-08 |  | PaW85 | 2.71E-08 |  | PC36 | 2.23E-08 |
| D28 | 1.81E-08 |  | PaW85 | 3.71E-09 |  | PC36 | 2.35E-08 |
| D28 | 1.28E-08 |  | PaW85 | 4.57E-09 |  | PC36 | 1.67E-09 |
| D28 | 1.25E-08 |  | PaW85 | 3.14E-09 |  | PC36 | 2.5E-09 |
| D28 | 3.28E-08 |  | PaW85 | 1.26E-08 |  | PC36 | 3.7E-08 |
| D28 | 5.28E-09 |  | PaW85 | 2.86E-09 |  | PC36 | 6.33E-09 |
| D28 | 4.57E-08 |  | PaW85 | 4.12E-08 |  | PC36 | 1.93E-08 |
| D28 | 1.47E-08 |  | PaW85 | 3.68E-08 |  | PC38 | 2.54E-09 |
| D2RT | 0 |  | PaW85 | 1.48E-08 |  | PC38 | 1.27E-09 |
| D2RT | 0 |  | PaW85 | 3.88E-08 |  | PC38 | 7.94E-09 |
| D2RT | 3.64E-09 |  | PaW85 | 2.84E-08 |  | PC38 | 1.9E-09 |
| D2RT | 5.45E-09 |  | PaW85 | 1.33E-08 |  | PC38 | 1.05E-08 |
| D2RT | 4.82E-09 |  | PaW85 | 2.04E-08 |  | PC38 | 2.3E-09 |
| D2RT | 0 |  | PaW85 | 2.91E-08 |  | PC38 | 3.22E-09 |
| D2RT | 0 |  | PaW85 | 2.35E-08 |  | PC38 | 1.84E-09 |
| D2RT | 0 |  | PaW85 | 1.05E-08 |  | PC38 | 4.14E-09 |
| D2RT | 0 |  | PaW85 | 7.47E-09 |  | PC38 | 3.45E-09 |
| D2RT | 0 |  | PaW85 | 6.02E-09 |  | PC38 | 1.49E-09 |
| D2RT | 0 |  | PaW85 | 1.95E-08 |  | PC38 | 1.01E-08 |
| D2RT | 0 |  | PaW85 | 1.86E-08 |  | PC38 | 4.48E-09 |
| D2RT | 0 |  | PaW85 | 1.06E-08 |  | PC38 | 1.19E-08 |
| D3 | 5.6E-09 |  | PaW85 | 1.12E-08 |  | PC38 | 6.27E-09 |
| D3 | 1.86E-08 |  | PaW85 | 2.42E-08 |  | PC39 | 1.86E-08 |
| D3 | 3.6E-09 |  | PaW85 | 4.42E-09 |  | PC39 | 1.59E-08 |
| D3 | 4.2E-09 |  | PaW85 | 1.51E-08 |  | PC39 | 7.95E-09 |
| D3 | 1.32E-08 |  | PaW85 | 6.23E-09 |  | PC39 | 2.52E-08 |
| D3 | 4.32E-08 |  | PaW85 | 7.11E-09 |  | PC39 | 8.22E-09 |
| D3 | 1.52E-08 |  | PaW85 | 2.44E-09 |  | PC39 | 8.6E-09 |
| D3 | 4.35E-08 |  | PaW85 | 6.44E-09 |  | PC39 | 8.8E-09 |
| D3 | 2.22E-08 |  | PaW85 | 7.11E-09 |  | PC39 | 6.4E-09 |
| D3 | 8.89E-09 |  | PaW85 | 1.78E-09 |  | PC39 | 4.8E-09 |
| D3 | 3.17E-08 |  | PaW85 | 6E-09 |  | PC39 | 9E-09 |
| D3 | 2.89E-08 |  | PaW85 | 8.4E-09 |  | PC39 | 7.5E-09 |
| D3 | 1.62E-08 |  | PaW85 | 8.4E-09 |  | PC39 | 4.5E-09 |
| D3 | 1.3E-08 |  | PaW85 | 4E-09 |  | PC39 | 9.5E-09 |
| D3 | 1.94E-08 |  | PaW85 | 1.1E-08 |  |  |  |

The survival of bacteria after UV-C irradiation.

| Strain | Without UV | Exposure to UV-irradiation | | |
| --- | --- | --- | --- | --- |
|  |  | 10 J/m2 | 20 J/m2 | 40 J/m2 |
| 2A20 | 4.00E+07 | 7.00E+06 | 6.00E+05 | 0.00E+00 |
| 2A20 | 4.00E+07 | 9.00E+06 | 1.10E+06 | 0.00E+00 |
| 2A20 | 3.00E+06 | 1.60E+06 | 3.00E+05 | 0.00E+00 |
| 2A20 | 4.00E+06 | 1.00E+06 | 4.00E+05 | 1.00E+04 |
| 2A20 | 1.50E+07 | 2.00E+06 | 7.00E+05 | 2.00E+04 |
| 2A20 | 2.00E+07 | 5.00E+06 | 1.20E+06 | 1.00E+05 |
| 2A38 | 9.00E+06 | 4.00E+06 | 1.30E+07 | 3.00E+06 |
| 2A38 | 1.00E+07 | 8.00E+06 | 1.20E+07 | 7.00E+06 |
| 2A38 | 9.00E+07 | 4.00E+07 | 6.00E+07 | 1.00E+07 |
| 2A38 | 2.00E+07 | 6.00E+07 | 9.00E+06 | 1.10E+07 |
| 2A38 | 9.00E+06 | 4.00E+07 | 4.00E+07 | 1.10E+07 |
| 2A38 | 5.00E+07 | 7.00E+07 | 7.00E+07 | 1.50E+07 |
| 2A38 | 2.50E+08 | 1.90E+08 | 1.20E+08 | 2.50E+07 |
| 2A38 | 2.30E+08 | 2.20E+08 | 2.20E+08 | 4.40E+07 |
| 2A38 | 2.80E+08 | 2.20E+08 | 1.90E+08 | 7.00E+07 |
| 2A38 | 3.00E+08 | 2.70E+08 | 2.30E+08 | 9.00E+07 |
| 2A54 | 1.30E+09 | 1.60E+09 | 4.00E+08 | 5.00E+07 |
| 2A54 | 3.00E+09 | 8.00E+08 | 8.00E+08 | 7.00E+07 |
| 2A54 | 6.00E+09 | 5.00E+09 | 2.30E+09 | 2.70E+08 |
| 2A54 | 6.00E+09 | 6.00E+09 | 3.00E+09 | 4.00E+08 |
| 2A54 | 3.00E+09 | 2.00E+09 | 2.20E+09 | 7.00E+08 |
| 2A54 | 6.00E+09 | 6.00E+09 | 3.00E+09 | 9.00E+08 |
| 2ANah4 | 1.10E+09 | 2.40E+08 | 7.00E+07 | 2.00E+05 |
| 2ANah4 | 1.30E+08 | 3.00E+07 | 1.90E+07 | 4.00E+05 |
| 2ANah4 | 4.00E+08 | 3.00E+08 | 1.40E+08 | 5.00E+05 |
| 2ANah4 | 2.50E+08 | 4.00E+07 | 1.90E+07 | 2.00E+06 |
| 2ANah4 | 4.00E+08 | 2.40E+08 | 8.00E+07 | 2.00E+06 |
| 2ANah4 | 4.00E+08 | 2.00E+08 | 1.20E+08 | 1.20E+07 |
| 2C23 | 8.00E+08 | 2.90E+08 | 8.00E+07 | 3.00E+05 |
| 2C23 | 1.20E+09 | 3.00E+08 | 4.00E+08 | 8.00E+05 |
| 2C23 | 1.40E+09 | 6.00E+08 | 8.00E+07 | 1.40E+06 |
| 2C23 | 1.40E+09 | 3.00E+08 | 8.00E+07 | 1.60E+06 |
| 2C23 | 1.50E+09 | 7.00E+08 | 3.00E+08 | 2.00E+06 |
| 2C23 | 3.00E+09 | 7.00E+08 | 1.70E+08 | 4.00E+06 |
| 2C41 | 7.00E+08 | 2.00E+08 | 1.00E+08 | 3.00E+05 |
| 2C41 | 6.00E+08 | 3.00E+08 | 5.00E+07 | 2.30E+06 |
| 2C41 | 5.00E+08 | 1.30E+08 | 9.00E+08 | 1.10E+07 |
| 2C41 | 5.00E+08 | 1.60E+08 | 1.70E+08 | 1.40E+07 |
| 2C41 | 1.60E+09 | 1.70E+09 | 1.40E+09 | 2.40E+08 |
| 2C41 | 2.50E+09 | 2.30E+09 | 1.50E+09 | 4.00E+08 |
| 2C63 | 7.00E+08 | 4.00E+08 | 8.00E+07 | 8.00E+05 |
| 2C63 | 1.00E+09 | 2.30E+08 | 1.70E+08 | 9.00E+05 |
| 2C63 | 2.00E+08 | 1.70E+08 | 7.00E+07 | 1.40E+06 |
| 2C63 | 3.00E+08 | 9.00E+07 | 8.00E+07 | 3.00E+06 |
| 2C63 | 2.20E+08 | 1.10E+08 | 1.30E+08 | 3.00E+06 |
| 2C63 | 4.00E+08 | 1.30E+08 | 7.00E+07 | 1.00E+07 |
| 2D61 | 2.00E+09 | 8.00E+08 | 6.00E+06 | 7.00E+04 |
| 2D61 | 1.80E+09 | 3.00E+08 | 1.50E+07 | 1.00E+05 |
| 2D61 | 2.00E+09 | 4.00E+08 | 4.00E+07 | 2.00E+05 |
| 2D61 | 1.80E+09 | 2.00E+08 | 5.00E+07 | 2.00E+05 |
| 2D61 | 3.00E+09 | 5.00E+08 | 1.00E+08 | 3.00E+05 |
| 2D61 | 2.00E+09 | 1.00E+09 | 4.00E+07 | 3.00E+05 |
| 2D61 | 2.00E+09 | 6.00E+08 | 4.00E+07 | 4.00E+05 |
| 2D66 | 7.00E+09 | 1.80E+09 | 1.50E+09 | 2.30E+07 |
| 2D66 | 3.50E+09 | 2.70E+09 | 4.00E+08 | 2.60E+07 |
| 2D66 | 5.00E+09 | 1.60E+09 | 1.50E+09 | 3.20E+07 |
| 2D66 | 4.00E+09 | 1.40E+09 | 1.10E+09 | 9.00E+07 |
| 2D66 | 7.00E+09 | 1.80E+09 | 1.00E+09 | 9.00E+07 |
| 2D66 | 2.20E+09 | 1.50E+09 | 1.50E+09 | 1.30E+08 |
| 2D67 | 4.90E+09 | 1.50E+09 | 1.20E+08 | 2.50E+06 |
| 2D67 | 8.00E+09 | 4.00E+08 | 8.00E+08 | 3.00E+06 |
| 2D67 | 4.00E+09 | 1.30E+09 | 4.00E+07 | 3.00E+06 |
| 2D67 | 4.00E+09 | 9.00E+08 | 4.00E+08 | 4.00E+06 |
| 2D67 | 5.00E+09 | 1.90E+09 | 4.00E+08 | 5.00E+06 |
| 2D67 | 7.00E+09 | 5.00E+09 | 1.00E+08 | 8.00E+06 |
| 2D67 | 5.00E+09 | 1.20E+09 | 2.20E+08 | 9.00E+06 |
| 2D67 | 3.00E+09 | 2.00E+09 | 1.30E+09 | 2.00E+07 |
| 2D67 | 2.20E+09 | 2.00E+09 | 1.00E+09 | 2.00E+07 |
| 2D67 | 3.00E+09 | 2.00E+09 | 2.00E+09 | 3.00E+07 |
| 2D67 | 1.40E+09 | 3.00E+09 | 3.00E+09 | 1.00E+08 |
| 2D67 | 2.00E+09 | 2.00E+09 | 3.00E+09 | 1.30E+08 |
| 2D67 | 1.10E+09 | 2.00E+09 | 1.10E+09 | 1.70E+08 |
| C52 | 1.50E+09 | 1.40E+09 | 4.00E+08 | 3.00E+06 |
| C52 | 4.00E+09 | 1.10E+09 | 1.80E+09 | 1.50E+07 |
| C52 | 3.00E+09 | 3.00E+09 | 3.80E+09 | 1.60E+07 |
| C52 | 1.30E+09 | 1.60E+09 | 6.00E+08 | 3.00E+07 |
| C52 | 3.00E+09 | 1.80E+09 | 4.00E+08 | 4.00E+07 |
| C52 | 2.40E+09 | 1.10E+09 | 1.40E+09 | 6.00E+07 |
| C52 | 1.00E+09 | 1.60E+09 | 8.00E+08 | 3.00E+08 |
| C52 | 1.90E+09 | 1.30E+09 | 5.00E+08 | 3.50E+08 |
| C52 | 1.30E+09 | 1.30E+09 | 1.10E+09 | 3.60E+08 |
| C52 | 1.00E+09 | 1.20E+09 | 8.00E+08 | 1.40E+09 |
| D113 | 1.30E+10 | 1.30E+09 | 4.00E+06 | 3.00E+04 |
| D113 | 1.10E+10 | 1.00E+09 | 1.80E+07 | 6.00E+04 |
| D113 | 1.10E+10 | 5.00E+08 | 1.90E+07 | 1.80E+05 |
| D113 | 1.00E+10 | 5.00E+08 | 8.00E+07 | 2.00E+05 |
| D113 | 1.10E+10 | 6.00E+08 | 4.00E+07 | 3.00E+05 |
| D14 | 7.00E+08 | 6.00E+07 | 4.00E+07 | 0.00E+00 |
| D14 | 3.00E+09 | 3.00E+08 | 5.00E+06 | 0.00E+00 |
| D14 | 8.00E+08 | 1.00E+08 | 4.00E+07 | 1.00E+04 |
| D14 | 1.00E+09 | 7.00E+08 | 2.00E+06 | 2.00E+04 |
| D14 | 7.00E+08 | 2.00E+08 | 4.00E+06 | 1.00E+05 |
| D14 | 1.20E+09 | 1.30E+08 | 8.00E+06 | 2.00E+05 |
| D14 | 2.30E+09 | 9.00E+08 | 6.00E+07 | 9.00E+05 |
| D28 | 5.00E+09 | 3.00E+04 | 1.00E+04 | 0.00E+00 |
| D28 | 9.00E+09 | 4.00E+05 | 1.10E+05 | 0.00E+00 |
| D28 | 8.00E+09 | 1.30E+05 | 1.00E+04 | 0.00E+00 |
| D28 | 5.00E+09 | 3.00E+05 | 5.00E+04 | 0.00E+00 |
| D28 | 1.20E+10 | 4.00E+04 | 1.00E+04 | 1.00E+04 |
| D28 | 7.00E+09 | 6.00E+05 | 1.00E+05 | 2.00E+04 |
| D3 | 5.00E+09 | 5.00E+08 | 1.50E+07 | 1.60E+05 |
| D3 | 1.10E+10 | 1.40E+09 | 6.00E+07 | 2.00E+05 |
| D3 | 1.10E+10 | 8.00E+07 | 7.00E+07 | 2.00E+05 |
| D3 | 7.00E+09 | 8.00E+08 | 1.80E+06 | 2.00E+05 |
| D3 | 1.10E+10 | 1.90E+08 | 1.80E+07 | 2.80E+05 |
| D3 | 1.30E+10 | 2.40E+08 | 7.00E+06 | 3.50E+05 |
| D3 | 7.00E+09 | 6.00E+08 | 3.00E+07 | 4.00E+05 |
| D45 | 9.00E+09 | 5.00E+07 | 2.00E+06 | 0.00E+00 |
| D45 | 1.40E+10 | 3.00E+07 | 6.00E+06 | 1.00E+04 |
| D45 | 1.50E+10 | 1.60E+08 | 2.00E+06 | 1.00E+04 |
| D45 | 1.20E+10 | 1.80E+08 | 2.20E+06 | 1.00E+04 |
| D45 | 1.10E+10 | 7.00E+07 | 1.80E+05 | 1.00E+04 |
| D45 | 1.10E+10 | 9.00E+07 | 3.00E+06 | 3.00E+04 |
| D45 | 6.00E+09 | 6.00E+07 | 3.00E+09 | 3.00E+06 |
| D45 | 5.00E+09 | 3.00E+09 | 1.00E+09 | 8.00E+06 |
| D66v | 1.20E+09 | 4.00E+09 | 2.00E+09 | 1.20E+07 |
| D66v | 1.80E+09 | 1.30E+09 | 1.00E+09 | 1.60E+07 |
| D66v | 1.30E+09 | 9.00E+07 | 4.00E+07 | 4.00E+07 |
| D66v | 1.70E+09 | 2.00E+09 | 6.00E+08 | 1.00E+08 |
| D66v | 1.20E+09 | 3.00E+09 | 8.00E+08 | 1.10E+08 |
| D66v | 1.60E+09 | 1.10E+09 | 4.00E+08 | 1.10E+08 |
| D66v | 4.00E+09 | 1.00E+09 | 1.00E+09 | 2.00E+08 |
| D66v | 1.20E+09 | 1.10E+09 | 6.00E+08 | 2.00E+08 |
| D66v | 6.00E+09 | 2.00E+09 | 1.40E+09 | 4.00E+08 |
| Hd1 | 2.40E+10 | 1.90E+08 | 8.00E+06 | 0.00E+00 |
| Hd1 | 8.00E+09 | 5.00E+08 | 2.20E+06 | 0.00E+00 |
| Hd1 | 2.10E+10 | 1.30E+08 | 8.00E+06 | 0.00E+00 |
| Hd1 | 7.00E+09 | 8.00E+08 | 1.80E+07 | 1.00E+04 |
| Hd1 | 1.30E+10 | 4.00E+08 | 1.40E+06 | 2.00E+04 |
| Hd1 | 1.30E+10 | 1.80E+09 | 3.30E+07 | 3.00E+04 |
| Hd1 | 1.90E+10 | 4.00E+08 | 6.00E+06 | 1.50E+05 |
| Hd16 | 2.30E+10 | 8.00E+09 | 9.00E+08 | 0.00E+00 |
| Hd16 | 9.00E+09 | 2.20E+09 | 3.80E+08 | 1.50E+05 |
| Hd16 | 1.50E+10 | 8.00E+09 | 1.00E+09 | 7.00E+05 |
| Hd16 | 8.00E+09 | 2.10E+09 | 4.00E+08 | 5.00E+06 |
| Hd16 | 1.10E+10 | 4.00E+09 | 3.00E+08 | 6.00E+06 |
| Hd6 | 1.50E+10 | 6.00E+08 | 3.00E+07 | 0.00E+00 |
| Hd6 | 1.00E+10 | 8.00E+08 | 3.00E+05 | 1.00E+04 |
| Hd6 | 1.00E+10 | 8.00E+08 | 1.00E+08 | 2.00E+04 |
| Hd6 | 1.00E+10 | 1.50E+09 | 6.00E+07 | 2.00E+04 |
| Hd6 | 9.00E+09 | 2.60E+09 | 3.00E+06 | 4.00E+04 |
| Hd6 | 8.00E+09 | 5.00E+08 | 5.00E+07 | 4.00E+04 |
| Hd6 | 1.00E+10 | 3.00E+08 | 2.00E+07 | 1.00E+05 |
| Hp2 | 3.90E+09 | 3.00E+08 | 1.30E+07 | 1.00E+04 |
| Hp2 | 5.00E+09 | 9.00E+07 | 3.00E+08 | 2.00E+04 |
| Hp2 | 6.00E+09 | 5.00E+09 | 1.00E+09 | 3.00E+05 |
| Hp2 | 8.00E+09 | 1.80E+09 | 5.00E+08 | 4.00E+05 |
| Hp2 | 6.00E+09 | 4.00E+09 | 8.00E+08 | 3.00E+06 |
| Hp2 | 4.00E+09 | 4.00E+09 | 1.60E+09 | 6.00E+06 |
| Hp5 | 1.10E+10 | 2.00E+09 | 9.00E+08 | 9.00E+04 |
| Hp5 | 1.00E+10 | 1.90E+09 | 1.20E+09 | 2.00E+05 |
| Hp5 | 8.00E+09 | 3.00E+09 | 5.00E+08 | 3.00E+05 |
| Hp5 | 1.10E+10 | 1.50E+09 | 8.00E+07 | 3.00E+05 |
| Hp5 | 6.00E+09 | 5.00E+09 | 3.00E+08 | 5.00E+05 |
| Hp5 | 5.00E+09 | 2.20E+09 | 4.00E+08 | 6.00E+05 |
| Hp5 | 7.00E+09 | 6.00E+09 | 1.20E+09 | 9.00E+05 |
| Hp5 | 9.00E+09 | 6.00E+09 | 5.00E+08 | 4.00E+06 |
| Hp6 | 8.00E+09 | 4.00E+09 | 8.00E+08 | 2.00E+05 |
| Hp6 | 8.00E+09 | 1.30E+09 | 9.00E+08 | 2.00E+05 |
| Hp6 | 5.00E+09 | 1.40E+09 | 1.10E+08 | 3.00E+05 |
| Hp6 | 6.00E+09 | 2.00E+09 | 9.00E+08 | 4.00E+05 |
| Hp6 | 5.00E+09 | 2.00E+09 | 7.00E+08 | 3.00E+06 |
| Hp6 | 7.00E+09 | 4.00E+09 | 9.00E+08 | 1.30E+07 |
| P3 | 6.00E+09 | 3.00E+08 | 2.00E+07 | 1.00E+04 |
| P3 | 5.00E+09 | 7.00E+08 | 2.30E+07 | 1.00E+04 |
| P3 | 7.00E+09 | 1.30E+08 | 4.00E+07 | 3.00E+04 |
| P3 | 5.00E+09 | 3.00E+09 | 5.00E+08 | 5.00E+04 |
| P3 | 1.00E+10 | 4.00E+08 | 1.90E+07 | 5.00E+04 |
| P3 | 7.00E+09 | 1.00E+09 | 4.00E+07 | 8.00E+04 |
| P3 | 1.10E+10 | 1.70E+09 | 2.00E+07 | 2.00E+05 |
| P3 | 8.00E+09 | 3.00E+08 | 3.00E+06 | 2.00E+05 |
| P4 | 1.20E+10 | 1.60E+07 | 3.00E+05 | 0.00E+00 |
| P4 | 7.00E+09 | 1.50E+06 | 9.00E+05 | 0.00E+00 |
| P4 | 1.60E+10 | 4.00E+06 | 1.10E+06 | 0.00E+00 |
| P4 | 9.00E+09 | 1.30E+07 | 7.00E+05 | 0.00E+00 |
| P4 | 6.00E+09 | 2.00E+06 | 2.00E+06 | 1.00E+04 |
| P48 | 1.40E+10 | 9.00E+07 | 7.00E+06 | 1.00E+04 |
| P48 | 2.00E+10 | 1.00E+08 | 2.00E+05 | 2.00E+04 |
| P48 | 7.00E+09 | 1.50E+09 | 1.00E+09 | 3.00E+05 |
| P48 | 7.00E+09 | 5.00E+09 | 1.00E+09 | 6.00E+05 |
| P48 | 8.00E+09 | 4.00E+09 | 1.20E+09 | 1.30E+06 |
| P48 | 7.00E+09 | 9.00E+08 | 1.00E+09 | 9.00E+06 |
| P48 | 5.00E+09 | 6.00E+09 | 8.00E+08 | 1.10E+07 |
| P48 | 5.00E+09 | 3.00E+09 | 6.00E+08 | 5.00E+07 |
| P49 | 1.00E+10 | 2.60E+08 | 5.00E+06 | 2.00E+04 |
| P49 | 6.00E+09 | 3.00E+08 | 7.00E+07 | 2.00E+04 |
| P49 | 9.00E+09 | 5.00E+07 | 1.80E+07 | 2.00E+04 |
| P49 | 1.20E+10 | 1.10E+08 | 1.10E+08 | 3.00E+04 |
| P49 | 8.00E+09 | 5.00E+08 | 5.00E+06 | 7.00E+04 |
| P6 | 6.00E+09 | 3.00E+08 | 4.00E+07 | 2.00E+04 |
| P6 | 7.00E+09 | 6.00E+08 | 1.50E+07 | 2.00E+04 |
| P6 | 8.00E+09 | 4.00E+09 | 4.00E+07 | 4.00E+04 |
| P6 | 1.30E+10 | 3.00E+08 | 5.00E+06 | 5.00E+04 |
| P6 | 6.00E+09 | 1.40E+08 | 4.00E+06 | 1.00E+05 |
| P6 | 6.00E+09 | 1.40E+09 | 2.00E+07 | 1.30E+05 |
| P6 | 1.30E+10 | 2.70E+08 | 1.60E+06 | 1.80E+05 |
| P69 | 8.00E+09 | 7.00E+08 | 1.00E+07 | 1.00E+04 |
| P69 | 1.00E+10 | 1.20E+09 | 2.00E+07 | 1.00E+04 |
| P69 | 3.00E+09 | 1.00E+09 | 0.00E+00 | 2.00E+04 |
| P69 | 6.00E+09 | 5.00E+08 | 2.00E+06 | 6.00E+04 |
| P69 | 7.00E+09 | 9.00E+08 | 3.00E+07 | 7.00E+04 |
| P69 | 9.00E+09 | 1.30E+09 | 1.30E+07 | 1.00E+05 |
| P85 | 1.20E+10 | 1.30E+09 | 1.80E+08 | 9.00E+04 |
| P85 | 8.00E+09 | 6.00E+08 | 3.00E+07 | 4.00E+05 |
| P85 | 9.00E+09 | 8.00E+08 | 9.00E+07 | 7.00E+05 |
| P85 | 4.00E+09 | 6.00E+08 | 9.00E+06 | 9.00E+05 |
| P85 | 7.00E+09 | 6.00E+08 | 6.00E+07 | 1.10E+06 |
| P85 | 1.00E+10 | 2.90E+09 | 1.80E+08 | 1.20E+06 |
| P85 | 1.00E+10 | 4.00E+08 | 4.00E+07 | 1.50E+06 |
| P86 | 1.30E+10 | 1.10E+09 | 4.00E+07 | 8.00E+04 |
| P86 | 3.00E+09 | - | 3.00E+08 | 7.00E+05 |
| P86 | 1.00E+10 | 3.60E+09 | 7.00E+08 | 1.70E+06 |
| P86 | 1.00E+10 | 7.00E+09 | 5.00E+08 | 2.00E+06 |
| P86 | 9.00E+09 | 1.20E+09 | 1.00E+09 | 2.40E+06 |
| P86 | 5.00E+09 | 3.00E+09 | 1.00E+09 | 4.40E+06 |
| P86 | 8.00E+09 | 2.10E+09 | 4.00E+08 | 7.00E+06 |
| P87 | 7.00E+09 | 5.00E+08 | 7.00E+06 | 0.00E+00 |
| P87 | 7.00E+09 | 1.00E+09 | 6.00E+07 | 1.00E+04 |
| P87 | 1.00E+10 | 8.00E+08 | 1.00E+08 | 1.00E+04 |
| P87 | 9.00E+09 | 9.00E+08 | 7.00E+06 | 1.00E+04 |
| P87 | 1.20E+10 | 1.00E+09 | 3.00E+07 | 4.00E+04 |
| P94 | 1.00E+10 | 7.00E+09 | 9.00E+08 | 2.00E+04 |
| P94 | 4.00E+09 | 1.80E+09 | 4.00E+08 | 2.00E+05 |
| P94 | 3.00E+09 | 1.50E+09 | 5.00E+08 | 2.00E+05 |
| P94 | 5.00E+09 | 1.00E+09 | 2.70E+07 | 5.00E+05 |
| P94 | 4.50E+09 | 2.50E+09 | 6.00E+07 | 1.60E+06 |
| PaW1 | 9.00E+09 | 4.00E+09 | 3.00E+08 | 5.00E+05 |
| PaW1 | 5.00E+09 | 3.00E+09 | 2.00E+08 | 8.00E+05 |
| PaW1 | 6.00E+09 | 4.00E+09 | 4.00E+08 | 8.00E+05 |
| PaW1 | 7.00E+09 | 1.80E+09 | 3.00E+08 | 1.00E+06 |
| PaW1 | 6.00E+09 | 3.00E+09 | 1.30E+08 | 1.70E+06 |
| PaW1 | 1.00E+10 | 1.70E+09 | 2.30E+08 | 1.70E+06 |
| PaW1 | 9.00E+09 | 1.50E+09 | 8.00E+07 | 1.90E+06 |
| PaW1 | 8.00E+09 | 3.00E+09 | 3.00E+08 | 2.00E+06 |
| PaW1 | 6.00E+09 | 2.00E+09 | 7.00E+08 | 2.60E+06 |
| PaW1 | 1.00E+10 | 6.00E+09 | 1.20E+08 | 3.00E+06 |
| PaW1 | 8.00E+09 | 3.00E+09 | 2.90E+08 | 4.00E+06 |
| PaW1 | 4.40E+09 | 9.00E+08 | 7.00E+08 | 4.00E+06 |
| PaW1 | 5.00E+09 | 1.40E+09 | 7.00E+08 | 4.00E+06 |
| PaW1 | 1.30E+10 | 5.00E+09 | 5.00E+08 | 4.00E+06 |
| PaW1 | 1.10E+10 | 5.00E+09 | 3.00E+08 | 4.00E+06 |
| PaW1 | 6.00E+09 | 2.80E+09 | 9.00E+07 | 5.00E+06 |
| PaW1 | 4.00E+09 | 4.00E+09 | 2.00E+08 | 5.00E+06 |
| PaW1 | 7.00E+09 | 4.00E+09 | 4.00E+08 | 6.00E+06 |
| PaW1 | 1.10E+10 | 1.30E+09 | 3.00E+08 | 6.00E+06 |
| PaW1 | 9.00E+09 | 4.00E+09 | 8.00E+08 | 8.00E+06 |
| PaW1 | 5.00E+09 | 1.90E+09 | 2.70E+08 | 8.00E+06 |
| PaW1 | 6.00E+09 | 1.30E+09 | 2.30E+08 | 9.00E+06 |
| PaW1 | 8.00E+09 | 3.00E+09 | 1.30E+09 | 1.00E+07 |
| PaW1 | 8.00E+09 | 7.00E+09 | 7.00E+08 | 1.00E+07 |
| PaW1 | 9.00E+09 | 8.00E+09 | 1.40E+09 | 2.00E+07 |
| PaW1 | 6.00E+09 | 6.00E+09 | 2.00E+09 | 3.00E+07 |
| PaW1 | 1.10E+10 | 7.00E+09 | 3.00E+09 | 5.00E+07 |
| PaW1 | 1.50E+10 | 2.60E+09 | 2.00E+09 | 6.00E+07 |
| PaW85 | 8.00E+09 | 7.00E+08 | 2.00E+06 | 0.00E+00 |
| PaW85 | 4.00E+09 | 7.00E+07 | 1.30E+07 | 1.00E+04 |
| PaW85 | 8.00E+09 | 4.00E+09 | 1.00E+07 | 2.00E+04 |
| PaW85 | 9.00E+09 | 6.00E+08 | 2.00E+06 | 2.00E+04 |
| PaW85 | 4.00E+09 | 6.00E+08 | 9.00E+06 | 2.00E+04 |
| PaW85 | 7.00E+09 | 3.00E+08 | 4.00E+06 | 3.00E+04 |
| PaW85 | 1.10E+10 | 7.00E+07 | 8.00E+06 | 3.00E+04 |
| PaW85 | 5.00E+09 | 1.00E+08 | 5.00E+06 | 4.00E+04 |
| PaW85 | 6.00E+09 | 1.40E+08 | 4.00E+06 | 4.00E+04 |
| PaW85 | 5.00E+09 | 7.00E+08 | 2.00E+06 | 5.00E+04 |
| PaW85 | 7.00E+09 | 5.00E+09 | 4.00E+07 | 8.00E+04 |
| PaW85 | 6.00E+09 | 1.10E+08 | 2.20E+06 | 9.00E+04 |
| PaW85 | 8.00E+09 | 8.00E+08 | 6.00E+06 | 1.00E+05 |
| PaW85 | 9.00E+09 | 6.00E+08 | 9.00E+06 | 1.00E+05 |
| PaW85 | 5.00E+09 | 2.20E+08 | 2.00E+06 | 1.00E+05 |
| PaW85 | 7.00E+09 | 9.00E+07 | 6.00E+06 | 1.20E+05 |
| PaW85 | 9.00E+09 | 1.80E+08 | 2.10E+06 | 1.20E+05 |
| PaW85 | 5.00E+09 | 1.00E+08 | 3.00E+06 | 1.40E+05 |
| PaW85 | 6.00E+09 | 4.00E+09 | 5.00E+07 | 2.00E+05 |
| PaW85 | 1.00E+10 | 2.00E+09 | 2.00E+08 | 2.00E+05 |
| PaW85 | 1.00E+10 | 4.00E+09 | 5.00E+07 | 2.00E+05 |
| PaW85 | 5.00E+09 | 6.00E+08 | 2.00E+06 | 2.00E+05 |
| PaW85 | 1.20E+10 | 5.00E+08 | 5.00E+06 | 2.00E+05 |
| PaW85 | 7.00E+09 | 1.00E+09 | 2.00E+07 | 3.00E+05 |
| PaW85 | 1.10E+10 | 1.10E+08 | 1.20E+07 | 3.00E+05 |
| PaW85 | 8.00E+09 | 6.00E+09 | 1.20E+07 | 4.00E+05 |
| PaW85 | 7.00E+09 | 1.20E+08 | 1.60E+07 | 5.00E+05 |
| PaW85 | 9.00E+09 | 1.30E+09 | 2.00E+07 | 1.20E+06 |
| PaWrulAB | 5.00E+09 | 1.30E+08 | 1.40E+08 | 2.00E+05 |
| PaWrulAB | 3.00E+09 | 9.00E+08 | 4.00E+08 | 2.60E+05 |
| PaWrulAB | 1.00E+10 | 4.00E+09 | 9.00E+07 | 3.40E+05 |
| PaWrulAB | 4.00E+09 | 8.00E+08 | 9.00E+07 | 4.00E+05 |
| PaWrulAB | 8.00E+09 | 3.00E+09 | 2.40E+08 | 4.00E+05 |
| PaWrulAB | 1.10E+10 | 2.00E+09 | 8.00E+07 | 6.00E+05 |
| PaWrulAB | 5.00E+09 | 2.40E+09 | 1.30E+08 | 6.00E+05 |
| PaWrulAB | 9.00E+09 | 1.30E+09 | 1.60E+08 | 6.00E+05 |
| PaWrulAB | 5.00E+09 | 8.00E+08 | 5.00E+07 | 7.00E+05 |
| PaWrulAB | 9.00E+09 | 1.80E+09 | 2.00E+08 | 7.00E+05 |
| PaWrulAB | 9.00E+09 | 6.00E+09 | 8.00E+08 | 8.00E+05 |
| PaWrulAB | 5.00E+09 | 1.40E+09 | 2.20E+07 | 8.00E+05 |
| PaWrulAB | 1.10E+10 | 3.00E+09 | 2.00E+08 | 1.00E+06 |
| PaWrulAB | 8.00E+09 | 4.00E+09 | 4.00E+08 | 1.00E+06 |
| PaWrulAB | 8.00E+09 | 3.00E+09 | 5.00E+08 | 1.20E+06 |
| PaWrulAB | 8.00E+09 | 9.00E+08 | 2.00E+07 | 1.30E+06 |
| PaWrulAB | 6.00E+09 | 5.00E+09 | 8.00E+07 | 1.40E+06 |
| PaWrulAB | 8.00E+09 | 6.00E+08 | 1.70E+08 | 1.40E+06 |
| PaWrulAB | 6.00E+09 | 1.80E+09 | 5.00E+08 | 3.00E+06 |
| PaWrulAB | 9.00E+09 | 9.00E+09 | 9.00E+08 | 4.00E+06 |
| PaWrulAB | 4.00E+09 | 5.00E+09 | 3.00E+08 | 4.00E+06 |
| PaWrulAB | 5.00E+09 | 4.00E+09 | 7.00E+08 | 5.00E+06 |
| PaWrulAB | 1.00E+10 | 1.20E+09 | 1.20E+08 | 5.00E+06 |
| PaWrulAB | 1.00E+10 | 7.00E+08 | 1.20E+08 | 6.00E+06 |
| PaWrulAB | 1.00E+10 | 9.00E+09 | 6.00E+08 | 7.00E+06 |
| PaWrulAB | 9.00E+09 | 1.10E+10 | 2.00E+09 | 7.00E+06 |
| PaWrulAB | 7.00E+09 | 7.00E+09 | 1.50E+08 | 8.00E+06 |
| PaWrulAB | 1.40E+09 | 1.60E+09 | 2.00E+08 | 9.00E+06 |
| PC13 | 3.30E+09 | 2.00E+05 | 1.00E+04 | 0.00E+00 |
| PC13 | 5.00E+09 | 3.00E+05 | 0.00E+00 | 0.00E+00 |
| PC13 | 8.00E+09 | 3.00E+05 | 4.00E+04 | 0.00E+00 |
| PC13 | 5.00E+09 | 6.00E+05 | 1.50E+05 | 1.00E+04 |
| PC13 | 4.00E+09 | 4.00E+06 | 1.50E+05 | 2.00E+04 |
| PC13 | 2.30E+09 | 6.00E+05 | 1.20E+05 | 3.00E+04 |
| PC14 | 1.00E+10 | 7.00E+06 | 7.00E+04 | 0.00E+00 |
| PC14 | 1.10E+10 | 2.00E+07 | 3.00E+05 | 0.00E+00 |
| PC14 | 8.00E+09 | 5.00E+07 | 6.00E+05 | 0.00E+00 |
| PC14 | 7.00E+09 | 1.30E+07 | 2.00E+06 | 0.00E+00 |
| PC14 | 1.00E+10 | 9.00E+06 | 3.00E+05 | 0.00E+00 |
| PC14 | 9.00E+09 | 7.00E+06 | 1.30E+06 | 0.00E+00 |
| PC15 | 1.00E+10 | 3.00E+06 | 5.00E+04 | 0.00E+00 |
| PC15 | 1.10E+10 | 6.00E+07 | 2.00E+05 | 0.00E+00 |
| PC15 | 6.00E+09 | 2.00E+07 | 8.00E+04 | 0.00E+00 |
| PC15 | 6.00E+09 | 1.60E+06 | 1.00E+04 | 1.00E+04 |
| PC15 | 8.00E+09 | 2.00E+06 | 6.00E+04 | 1.00E+04 |
| PC15 | 8.00E+09 | 2.00E+06 | 7.00E+04 | 1.00E+04 |
| PC16 | 5.00E+09 | 7.00E+06 | 4.00E+06 | 0.00E+00 |
| PC16 | 8.00E+09 | 5.00E+07 | 1.10E+05 | 0.00E+00 |
| PC16 | 8.00E+09 | 4.00E+06 | 2.00E+06 | 0.00E+00 |
| PC16 | 5.00E+09 | 9.00E+06 | 3.00E+05 | 0.00E+00 |
| PC16 | 8.00E+09 | 2.00E+06 | 1.40E+05 | 6.00E+03 |
| PC16 | 8.00E+09 | 6.00E+06 | 8.00E+05 | 1.00E+04 |
| PC16 | 1.20E+10 | 6.00E+07 | 4.00E+06 | 2.00E+04 |
| PC17 | 5.00E+09 | 4.00E+08 | 2.30E+07 | 2.80E+06 |
| PC17 | 7.00E+09 | 4.00E+09 | 5.00E+07 | 3.00E+06 |
| PC17 | 8.00E+09 | 9.00E+08 | 4.00E+08 | 2.00E+07 |
| PC17 | 7.00E+09 | 2.00E+09 | 2.00E+08 | 2.00E+07 |
| PC17 | 4.00E+09 | 4.00E+09 | 3.00E+08 | 4.00E+07 |
| PC17 | 4.00E+09 | 1.00E+09 | 6.00E+08 | 1.30E+08 |
| PC18 | 8.00E+09 | 2.00E+08 | 4.00E+06 | 0.00E+00 |
| PC18 | 6.00E+09 | 3.00E+08 | 8.00E+05 | 0.00E+00 |
| PC18 | 1.00E+10 | 4.00E+09 | 8.00E+06 | 0.00E+00 |
| PC18 | 1.40E+10 | 1.00E+09 | 5.00E+08 | 0.00E+00 |
| PC18 | 1.00E+10 | 2.00E+08 | 5.00E+07 | 0.00E+00 |
| PC18 | 1.00E+10 | 6.00E+08 | 5.00E+06 | 0.00E+00 |
| PC18 | 1.50E+10 | 1.30E+09 | 4.00E+06 | 1.00E+04 |
| PC18 | 7.00E+09 | 8.00E+08 | 6.00E+06 | 1.00E+04 |
| PC18 | 8.00E+09 | 6.00E+08 | 5.00E+06 | 1.00E+04 |
| PC18 | 1.40E+10 | 4.00E+09 | 8.00E+07 | 2.00E+04 |
| PC18 | 6.00E+09 | 5.00E+08 | 6.00E+05 | 4.00E+04 |
| PC18 | 9.00E+09 | 1.00E+09 | 2.00E+06 | 4.00E+04 |
| PC18 | 1.00E+10 | 3.00E+09 | 3.00E+08 | 4.00E+05 |
| PC20 | 3.00E+09 | 3.00E+08 | 1.30E+07 | 0.00E+00 |
| PC20 | 4.00E+09 | 5.00E+08 | 3.00E+06 | 0.00E+00 |
| PC20 | 3.00E+09 | 2.00E+08 | 2.00E+07 | 0.00E+00 |
| PC20 | 4.00E+09 | 1.30E+09 | 3.00E+07 | 1.00E+04 |
| PC20 | 7.00E+09 | 1.80E+09 | 8.00E+06 | 3.00E+04 |
| PC20 | 6.00E+09 | 2.00E+09 | 2.00E+08 | 2.00E+05 |
| PC20 | 6.00E+09 | 1.60E+09 | 3.00E+08 | 1.40E+06 |
| PC24 | 1.30E+10 | 1.30E+09 | 2.00E+06 | 0.00E+00 |
| PC24 | 1.50E+10 | 5.00E+08 | 3.00E+07 | 0.00E+00 |
| PC24 | 1.30E+10 | 2.00E+08 | 5.00E+06 | 0.00E+00 |
| PC24 | 1.50E+10 | 6.00E+08 | 2.00E+05 | 4.00E+03 |
| PC24 | 1.80E+10 | 1.00E+08 | 7.00E+05 | 1.00E+04 |
| PC24 | 1.10E+10 | 6.00E+07 | 3.00E+06 | 1.00E+04 |
| PC24 | 1.30E+10 | 8.00E+08 | 1.10E+07 | 8.00E+04 |
| PC30 | 1.50E+10 | 8.00E+06 | 3.00E+05 | 0.00E+00 |
| PC30 | 1.00E+10 | 3.00E+07 | 2.00E+04 | 0.00E+00 |
| PC30 | 7.00E+09 | 4.00E+06 | 3.00E+05 | 0.00E+00 |
| PC30 | 1.00E+10 | 3.00E+07 | 2.00E+05 | 0.00E+00 |
| PC30 | 7.00E+09 | 5.00E+06 | 4.00E+04 | 0.00E+00 |
| PC30 | 1.00E+10 | 1.10E+07 | 3.00E+05 | 1.00E+04 |
| PC34 | 7.00E+09 | 0.00E+00 | 2.00E+04 | 0.00E+00 |
| PC34 | 1.00E+10 | 3.00E+05 | 1.00E+04 | 0.00E+00 |
| PC34 | 6.00E+09 | 2.00E+04 | 0.00E+00 | 0.00E+00 |
| PC34 | 8.00E+09 | 1.60E+05 | 2.00E+04 | 1.00E+04 |
| PC34 | 7.00E+09 | 3.00E+05 | 2.00E+04 | 1.00E+04 |
| PC34 | 4.00E+09 | 4.00E+05 | 1.00E+04 | 1.00E+04 |
| PC36 | 6.00E+09 | 5.00E+06 | 4.00E+05 | 0.00E+00 |
| PC36 | 7.00E+09 | 2.00E+07 | 3.00E+05 | 0.00E+00 |
| PC36 | 1.10E+10 | 1.60E+07 | 4.00E+05 | 1.00E+04 |
| PC36 | 8.00E+09 | 7.00E+07 | 5.00E+04 | 1.00E+04 |
| PC36 | 6.00E+09 | 1.40E+08 | 7.00E+06 | 4.00E+04 |
| PC36 | 8.00E+09 | 9.00E+07 | 2.00E+06 | 4.00E+04 |
| PC38 | 6.00E+09 | 7.00E+08 | 8.00E+07 | 1.00E+04 |
| PC38 | 9.00E+09 | 5.00E+08 | 8.00E+06 | 2.00E+04 |
| PC38 | 3.50E+09 | 1.60E+09 | 1.10E+08 | 2.00E+04 |
| PC38 | 4.00E+09 | 1.00E+09 | 3.00E+07 | 8.00E+04 |
| PC38 | 6.00E+09 | 1.20E+09 | 4.00E+06 | 1.30E+05 |
| PC38 | 8.00E+09 | 1.10E+09 | 1.30E+07 | 2.00E+05 |
| PC39 | 8.00E+09 | 9.00E+07 | 1.00E+06 | 1.00E+04 |
| PC39 | 1.90E+10 | 6.00E+07 | 4.00E+06 | 1.00E+04 |
| PC39 | 1.00E+10 | 1.90E+08 | 1.20E+06 | 1.00E+04 |
| PC39 | 4.00E+09 | 8.00E+07 | 0.00E+00 | 2.00E+04 |
| PC39 | 7.00E+09 | 5.00E+07 | 2.00E+06 | 3.00E+04 |
| PC39 | 8.00E+09 | 2.20E+08 | 2.00E+04 | 4.00E+04 |

The UV-induced (100 J/m^2^) Rif^r^ mutant frequencies.

| Strain | UV-induced | Strain | UV-induced | Strain | UV-induced |
| --- | --- | --- | --- | --- | --- |
| 2A20 | 2.05E-08 | C52 | 4.25E-06 | P94 | 1.88E-09 |
| 2A20 | 2.09E-08 | C52 | 4.55E-06 | P94 | 1.88E-09 |
| 2A20 | 1.40E-07 | C52 | 4.90E-06 | P94 | 3.75E-09 |
| 2A20 | 1.75E-07 | C52 | 5.03E-06 | P94 | 5.63E-08 |
| 2A20 | 1.94E-07 | C52 | 5.25E-06 | P94 | 6.00E-08 |
| 2A20 | 2.33E-07 | D66v | 5.66E-08 | P94 | 6.57E-08 |
| 2A20 | 2.56E-07 | D66v | 9.81E-08 | P94 | 8.14E-08 |
| 2A20 | 3.20E-07 | D66v | 1.06E-07 | P94 | 9.00E-08 |
| 2A20 | 3.25E-07 | D66v | 1.06E-07 | P94 | 1.04E-07 |
| 2A20 | 3.50E-07 | D66v | 2.15E-07 | P94 | 1.06E-07 |
| 2A20 | 3.68E-07 | D66v | 2.42E-07 | P94 | 1.22E-07 |
| 2A20 | 4.08E-07 | D66v | 2.83E-07 | P94 | 1.43E-07 |
| 2A20 | 4.72E-07 | D66v | 2.90E-07 | P94 | 1.53E-07 |
| 2A20 | 4.77E-07 | D66v | 3.25E-07 | P94 | 1.63E-07 |
| 2A38 | 4.75E-07 | D66v | 3.55E-07 | P94 | 1.70E-07 |
| 2A38 | 7.17E-07 | D66v | 4.83E-07 | P94 | 1.76E-07 |
| 2A38 | 7.45E-07 | D66v | 5.10E-07 | P94 | 1.82E-07 |
| 2A38 | 9.00E-07 | D66v | 5.10E-07 | P94 | 1.90E-07 |
| 2A38 | 1.03E-06 | D66v | 5.20E-07 | P94 | 2.16E-07 |
| 2A38 | 1.12E-06 | D66v | 5.30E-07 | PaW1 | 6.38E-09 |
| 2A38 | 1.27E-06 | D66v | 5.42E-07 | PaW1 | 7.14E-09 |
| 2A38 | 1.40E-06 | D66v | 6.30E-07 | PaW1 | 1.00E-08 |
| 2A38 | 1.45E-06 | D66v | 6.30E-07 | PaW1 | 1.72E-08 |
| 2A38 | 2.03E-06 | D66v | 6.45E-07 | PaW1 | 1.73E-08 |
| 2A38 | 2.22E-06 | D66v | 6.45E-07 | PaW1 | 2.77E-08 |
| 2A38 | 3.15E-06 | D66v | 6.50E-07 | PaW1 | 3.96E-08 |
| 2A38 | 4.19E-06 | D66v | 6.55E-07 | PaW1 | 4.23E-08 |
| 2A38 | 4.29E-06 | D66v | 7.17E-07 | PaW1 | 4.32E-08 |
| 2A38 | 4.88E-06 | D66v | 1.36E-06 | PaW1 | 4.61E-08 |
| 2A38 | 5.42E-06 | Hd16 | 1.00E-08 | PaW1 | 4.80E-08 |
| 2A38 | 5.57E-06 | Hd16 | 1.60E-08 | PaW1 | 5.84E-08 |
| 2A38 | 5.66E-06 | Hd16 | 6.36E-08 | PaW1 | 7.03E-08 |
| 2A38 | 6.76E-06 | Hd16 | 7.22E-08 | PaW1 | 7.14E-08 |
| 2A54 | 5.24E-07 | Hd16 | 8.40E-08 | PaW1 | 7.91E-08 |
| 2A54 | 7.49E-07 | Hd16 | 8.76E-08 | PaW1 | 9.71E-08 |
| 2A54 | 8.08E-07 | Hd16 | 8.78E-08 | PaW1 | 1.02E-07 |
| 2A54 | 8.48E-07 | Hd16 | 1.07E-07 | PaW1 | 1.14E-07 |
| 2A54 | 8.96E-07 | Hd16 | 1.18E-07 | PaW1 | 1.16E-07 |
| 2A54 | 9.08E-07 | Hd16 | 1.19E-07 | PaW1 | 1.22E-07 |
| 2A54 | 9.20E-07 | Hd16 | 1.21E-07 | PaW1 | 1.28E-07 |
| 2A54 | 9.28E-07 | Hd16 | 1.30E-07 | PaW1 | 1.46E-07 |
| 2A54 | 9.46E-07 | Hd16 | 1.37E-07 | PaW1 | 1.91E-07 |
| 2A54 | 9.52E-07 | Hd16 | 1.39E-07 | PaW1 | 2.00E-07 |
| 2A54 | 9.94E-07 | Hd16 | 1.56E-07 | PaW1 | 2.24E-07 |
| 2A54 | 1.15E-06 | Hd16 | 1.68E-07 | PaW1 | 3.32E-07 |
| 2A54 | 1.18E-06 | Hd16 | 1.70E-07 | PaW1 | 3.80E-07 |
| 2A54 | 1.21E-06 | Hd16 | 2.28E-07 | PaW1 | 3.96E-07 |
| 2A54 | 1.26E-06 | Hd16 | 2.32E-07 | PaW1 | 3.96E-07 |
| 2Anah4 | 1.43E-07 | Hp2 | 7.23E-08 | PaW1 | 4.00E-07 |
| 2Anah4 | 1.54E-07 | Hp2 | 7.85E-08 | PaW1 | 4.29E-07 |
| 2Anah4 | 1.55E-07 | Hp2 | 8.68E-08 | PaW1 | 4.52E-07 |
| 2Anah4 | 1.89E-07 | Hp2 | 9.14E-08 | PaW1 | 4.54E-07 |
| 2Anah4 | 1.89E-07 | Hp2 | 9.77E-08 | PaW1 | 4.73E-07 |
| 2Anah4 | 1.99E-07 | Hp2 | 1.13E-07 | PaW1 | 4.96E-07 |
| 2Anah4 | 2.00E-07 | Hp2 | 1.30E-07 | PaW1 | 5.36E-07 |
| 2Anah4 | 2.07E-07 | Hp2 | 1.35E-07 | PaW1 | 5.53E-07 |
| 2Anah4 | 2.37E-07 | Hp2 | 1.39E-07 | PaW1 | 5.96E-07 |
| 2Anah4 | 2.44E-07 | Hp2 | 1.42E-07 | PaW1 | 6.00E-07 |
| 2Anah4 | 2.47E-07 | Hp2 | 1.52E-07 | PaW85 | 1.20E-09 |
| 2Anah4 | 2.75E-07 | Hp2 | 1.53E-07 | PaW85 | 2.46E-09 |
| 2Anah4 | 2.76E-07 | Hp2 | 1.54E-07 | PaW85 | 2.81E-09 |
| 2Anah4 | 3.00E-07 | Hp2 | 1.58E-07 | PaW85 | 3.51E-09 |
| 2Anah4 | 3.24E-07 | Hp2 | 1.62E-07 | PaW85 | 3.64E-09 |
| 2Anah4 | 5.72E-07 | Hp2 | 1.72E-07 | PaW85 | 4.85E-09 |
| 2Anah4 | 5.78E-07 | Hp2 | 1.79E-07 | PaW85 | 4.91E-09 |
| 2Anah4 | 6.53E-07 | Hp2 | 1.88E-07 | PaW85 | 5.96E-09 |
| 2Anah4 | 6.88E-07 | Hp2 | 1.90E-07 | PaW85 | 6.00E-09 |
| 2C41 | 1.16E-07 | Hp5 | 3.91E-09 | PaW85 | 7.72E-09 |
| 2C41 | 1.87E-07 | Hp5 | 1.04E-08 | PaW85 | 8.40E-09 |
| 2C41 | 2.19E-07 | Hp5 | 1.17E-08 | PaW85 | 8.48E-09 |
| 2C41 | 2.20E-07 | Hp5 | 7.32E-08 | PaW85 | 8.77E-09 |
| 2C41 | 2.58E-07 | Hp5 | 1.26E-07 | PaW85 | 8.77E-09 |
| 2C41 | 2.62E-07 | Hp5 | 1.37E-07 | PaW85 | 9.09E-09 |
| 2C41 | 3.03E-07 | Hp5 | 1.44E-07 | PaW85 | 9.82E-09 |
| 2C41 | 3.17E-07 | Hp5 | 1.59E-07 | PaW85 | 1.02E-08 |
| 2C41 | 3.37E-07 | Hp5 | 1.82E-07 | PaW85 | 1.14E-08 |
| 2C41 | 3.46E-07 | Hp5 | 2.12E-07 | PaW85 | 1.20E-08 |
| 2C41 | 3.47E-07 | Hp5 | 2.13E-07 | PaW85 | 1.27E-08 |
| 2C41 | 3.92E-07 | Hp5 | 2.16E-07 | PaW85 | 1.42E-08 |
| 2C41 | 4.32E-07 | Hp5 | 2.19E-07 | PaW85 | 1.51E-08 |
| 2C41 | 6.04E-07 | Hp5 | 2.22E-07 | PaW85 | 1.53E-08 |
| 2C41 | 6.04E-07 | Hp5 | 2.28E-07 | PaW85 | 1.55E-08 |
| 2C41 | 6.24E-07 | Hp5 | 2.63E-07 | PaW85 | 1.59E-08 |
| 2C41 | 6.57E-07 | Hp5 | 3.21E-07 | PaW85 | 1.60E-08 |
| 2C41 | 6.83E-07 | Hp5 | 3.28E-07 | PaW85 | 1.70E-08 |
| 2C41 | 7.67E-07 | Hp5 | 3.57E-07 | PaW85 | 1.71E-08 |
| 2C63 | 2.95E-07 | Hp6 | 1.67E-08 | PaW85 | 1.92E-08 |
| 2C63 | 3.12E-07 | Hp6 | 1.95E-08 | PaW85 | 2.03E-08 |
| 2C63 | 3.70E-07 | Hp6 | 2.00E-08 | PaW85 | 2.28E-08 |
| 2C63 | 3.85E-07 | Hp6 | 2.25E-08 | PaW85 | 2.61E-08 |
| 2C63 | 4.30E-07 | Hp6 | 2.47E-08 | PaW85 | 3.73E-08 |
| 2C63 | 4.71E-07 | Hp6 | 2.58E-08 | PaW85 | 4.70E-08 |
| 2C63 | 6.00E-07 | Hp6 | 2.83E-08 | PaW85 | 1.04E-07 |
| 2C63 | 6.43E-07 | Hp6 | 2.88E-08 | PaW85 | 1.65E-07 |
| 2C63 | 6.67E-07 | Hp6 | 3.13E-08 | PaW85 | 2.33E-07 |
| 2C63 | 7.29E-07 | Hp6 | 3.67E-08 | PaW85 | 2.62E-07 |
| 2C63 | 7.30E-07 | Hp6 | 4.51E-08 | PaW85 | 9.43E-07 |
| 2C63 | 7.41E-07 | Hp6 | 4.63E-08 | PaWrulAB | 3.00E-09 |
| 2C63 | 8.28E-07 | Hp6 | 4.78E-08 | PaWrulAB | 4.38E-09 |
| 2C63 | 8.50E-07 | Hp6 | 5.46E-08 | PaWrulAB | 1.00E-08 |
| 2D66 | 2.47E-07 | Hp6 | 5.51E-08 | PaWrulAB | 1.03E-08 |
| 2D66 | 2.66E-07 | Hp6 | 5.63E-08 | PaWrulAB | 1.27E-08 |
| 2D66 | 3.33E-07 | Hp6 | 5.66E-08 | PaWrulAB | 1.35E-08 |
| 2D66 | 3.43E-07 | Hp6 | 5.75E-08 | PaWrulAB | 1.94E-08 |
| 2D66 | 3.90E-07 | Hp6 | 5.82E-08 | PaWrulAB | 2.06E-08 |
| 2D66 | 4.02E-07 | Hp6 | 6.13E-08 | PaWrulAB | 2.57E-08 |
| 2D66 | 4.61E-07 | Hp6 | 6.22E-08 | PaWrulAB | 4.75E-08 |
| 2D66 | 5.00E-07 | Hp6 | 6.37E-08 | PaWrulAB | 4.97E-08 |
| 2D66 | 5.03E-07 | Hp6 | 6.41E-08 | PaWrulAB | 6.24E-08 |
| 2D66 | 5.11E-07 | Hp6 | 6.60E-08 | PaWrulAB | 8.37E-08 |
| 2D66 | 5.15E-07 | Hp6 | 7.92E-08 | PaWrulAB | 1.16E-07 |
| 2D66 | 5.20E-07 | Hp6 | 9.33E-08 | PaWrulAB | 2.33E-07 |
| 2D66 | 5.30E-07 | Hp6 | 1.53E-07 | PaWrulAB | 2.33E-07 |
| 2D66 | 5.61E-07 | Hp6 | 2.27E-07 | PaWrulAB | 4.65E-07 |
| 2D66 | 6.26E-07 | Hp6 | 2.38E-07 | PaWrulAB | 4.67E-07 |
| 2D66 | 6.70E-07 | Hp6 | 2.51E-07 | PaWrulAB | 4.93E-07 |
| 2D66 | 6.98E-07 | Hp6 | 2.84E-07 | PC17 | 0 |
| 2D66 | 7.14E-07 | Hp6 | 3.28E-07 | PC17 | 0 |
| 2D66 | 7.23E-07 | Hp6 | 3.30E-07 | PC17 | 0 |
| 2D66 | 8.08E-07 | P48 | 1.67E-09 | PC17 | 1.04E-09 |
| 2D67 | 0.00E+00 | P48 | 2.00E-09 | PC17 | 2E-09 |
| 2D67 | 0.00E+00 | P48 | 2.17E-09 | PC17 | 2E-09 |
| 2D67 | 1.25E-09 | P48 | 4.17E-09 | PC17 | 5.18E-09 |
| 2D67 | 1.25E-09 | P48 | 2.54E-08 | PC17 | 7.87E-09 |
| 2D67 | 1.74E-09 | P48 | 1.17E-07 | PC17 | 9.33E-09 |
| 2D67 | 1.74E-09 | P48 | 2.99E-07 | PC17 | 1.26E-08 |
| 2D67 | 2.50E-09 | P48 | 3.31E-07 | PC17 | 2.05E-08 |
| 2D67 | 2.50E-09 | P48 | 3.32E-07 | PC17 | 2.69E-08 |
| 2D67 | 5.00E-09 | P48 | 3.36E-07 | PC17 | 2.9E-08 |
| 2D67 | 5.00E-09 | P48 | 3.79E-07 | PC17 | 3.8E-08 |
| 2D67 | 6.25E-09 | P48 | 4.20E-07 | PC17 | 5.13E-08 |
| 2D67 | 6.25E-09 | P48 | 4.46E-07 | PC17 | 1.26E-07 |
| 2D67 | 8.42E-09 | P48 | 4.56E-07 | PC17 | 1.29E-07 |
| 2D67 | 1.05E-08 | P48 | 5.11E-07 | PC17 | 2.66E-07 |
| 2D67 | 1.13E-08 | P48 | 5.22E-07 | PC17 | 9.07E-07 |
| 2D67 | 2.00E-08 | P48 | 6.53E-07 | 2C23 | 1.35E-07 |
| 2D67 | 3.00E-08 | P48 | 6.89E-07 | 2C23 | 1.63E-07 |
| 2D67 | 4.63E-08 | P48 | 8.55E-07 | 2C23 | 9.51E-08 |
| 2D67 | 4.63E-08 | P86 | 5.83E-10 | 2C23 | 1.04E-07 |
| 2D67 | 6.00E-08 | P86 | 5.83E-10 | 2C23 | 1.1E-07 |
| 2D67 | 9.00E-08 | P86 | 3.30E-09 | 2C23 | 1.74E-07 |
| 2D67 | 1.50E-07 | P86 | 6.05E-09 | 2C23 | 6E-08 |
| 2D67 | 2.09E-07 | P86 | 7.57E-09 | 2C23 | 8.32E-08 |
| 2D67 | 5.43E-07 | P86 | 2.56E-08 | 2C23 | 1.01E-07 |
| C52 | 2.19E-07 | P86 | 4.56E-08 | 2C23 | 1.2E-07 |
| C52 | 2.50E-07 | P86 | 5.20E-08 | 2C23 | 6.6E-08 |
| C52 | 3.08E-07 | P86 | 8.60E-08 | 2C23 | 1.07E-07 |
| C52 | 3.25E-07 | P86 | 9.30E-08 | 2C23 | 2.28E-08 |
| C52 | 3.30E-07 | P86 | 1.04E-07 | 2C23 | 4.2E-08 |
| C52 | 3.30E-07 | P86 | 1.28E-07 | 2C23 | 9.48E-08 |
| C52 | 4.56E-07 | P86 | 1.30E-07 | 2C23 | 6.16E-08 |
| C52 | 6.88E-07 | P86 | 1.32E-07 | 2C23 | 6.48E-08 |
| C52 | 1.70E-06 | P86 | 1.33E-07 | 2C23 | 6.32E-08 |
| C52 | 3.32E-06 | P86 | 1.47E-07 | 2C23 | 6.16E-08 |
| C52 | 3.53E-06 | P86 | 1.58E-07 | 2C23 | 7.27E-08 |
| C52 | 3.85E-06 | P86 | 1.68E-07 |  |  |
| C52 | 4.05E-06 | P86 | 1.84E-07 |  |  |

The UV-induced (5 J/m^2^) Rif^r^ mutant frequencies.

| Strain | UV-induced | Strain | UV-induced |
| --- | --- | --- | --- |
| PaW85 | 7.72E-08 | PaW1 | 3.36E-07 |
| PaW85 | 7.81E-08 | PaW1 | 4.00E-07 |
| PaW85 | 6.33E-08 | PaW1 | 3.60E-07 |
| PaW85 | 1.10E-07 | PaW1 | 2.38E-07 |
| PaW85 | 1.29E-07 | PaW1 | 2.90E-07 |
| PaW85 | 3.32E-08 | PaW1 | 2.07E-07 |
| PaW85 | 7.13E-08 | PaW1 | 3.25E-07 |
| PaW85 | 7.05E-08 | PaW1 | 5.49E-07 |
| PaW85 | 6.84E-08 | PaW1 | 2.61E-07 |
| PaW85 | 6.95E-08 | PaW1 | 2.61E-07 |
| PaW85 | 4.69E-08 | PC20 | 1.13E-06 |
| PaW85 | 8.82E-08 | PC20 | 8.35E-07 |
| PaW85 | 1.14E-07 | PC20 | 8.35E-07 |
| PaW85 | 1.18E-07 | PC20 | 7.50E-07 |
| PaW85 | 4.69E-08 | PC20 | 9.63E-07 |
| PaW85 | 7.63E-08 | PC20 | 5.81E-07 |
| PaW85 | 1.47E-07 | PC20 | 8.07E-07 |
| PaW85 | 8.72E-08 | PC20 | 5.40E-07 |
| PaW85 | 1.61E-07 | PC20 | 5.88E-07 |
| PaW85 | 1.12E-07 | PC20 | 5.81E-07 |
| PaWrulAB | 4.40E-07 | PC20 | 2.29E-07 |
| PaWrulAB | 5.60E-07 | PC20 | 3.57E-07 |
| PaWrulAB | 8.13E-07 | PC20 | 4.19E-07 |
| PaWrulAB | 5.35E-07 | PC20 | 3.24E-07 |
| PaWrulAB | 4.92E-07 | PC20 | 4.10E-07 |
| PaWrulAB | 5.41E-07 | PC24 | 4.46E-07 |
| PaWrulAB | 5.83E-07 | PC24 | 3.84E-07 |
| PaWrulAB | 5.51E-07 | PC24 | 4.04E-07 |
| PaWrulAB | 5.26E-07 | PC24 | 3.64E-07 |
| PaWrulAB | 5.98E-07 | PC24 | 4.46E-07 |
| PaWrulAB | 3.97E-07 | PC24 | 6.58E-07 |
| PaWrulAB | 3.52E-07 | PC24 | 6.58E-07 |
| PaWrulAB | 3.57E-07 | PC24 | 6.58E-07 |
| PaWrulAB | 3.52E-07 | PC24 | 6.05E-07 |
| PaWrulAB | 2.65E-07 | PC24 | 6.58E-07 |
| PaW1 | 2.80E-07 | PC24 | 1.14E-06 |
| PaW1 | 3.54E-07 | PC24 | 1.20E-06 |
| PaW1 | 1.56E-07 | PC24 | 1.39E-06 |
| PaW1 | 2.66E-07 | PC24 | 1.52E-06 |
| PaW1 | 2.88E-07 | PC24 | 1.43E-06 |

The expression rate of error-prone DNA polymerases obtained with RT-qPCR experiments. Expression rate is normalized against corresponding expression of *trpA* gene. During normalization process the data obtained for *trpA* expression rate is averaged in each experiment. Treatment one is without MMC and second is with the addition of MMC (2µg/ml).

| Gene of interest | Expression rate | Treatment |
| --- | --- | --- |
| DnaE2_PaW1 | 0.040 | 1 |
| DnaE2_PaW1 | 0.057 | 1 |
| DnaE2_PaW1 | 0.065 | 1 |
| DnaE2_PaW1 | 0.048 | 1 |
| DnaE2_PaW1 | 0.099 | 1 |
| DnaE2_PaW1 | 0.102 | 1 |
| DnaE2_PaW1 | 0.113 | 1 |
| DnaE2_PaW1 | 0.121 | 1 |
| DnaE2_PaW1 | 0.020 | 1 |
| DnaE2_PaW1 | 0.024 | 1 |
| DnaE2_PaW1 | 0.015 | 1 |
| DnaE2_PaW1 | 0.023 | 1 |
| DnaE2_PaW1 | 0.514 | 2 |
| DnaE2_PaW1 | 0.237 | 2 |
| DnaE2_PaW1 | 0.284 | 2 |
| DnaE2_PaW1 | 0.459 | 2 |
| DnaE2_PaW1 | 0.619 | 2 |
| DnaE2_PaW1 | 0.529 | 2 |
| DnaE2_PaW1 | 0.578 | 2 |
| DnaE2_PaW1 | 0.249 | 2 |
| DnaE2_PaW1 | 0.211 | 2 |
| DnaE2_PaW1 | 0.356 | 2 |
| DnaE2_PaW1 | 0.339 | 2 |
| RulB_PaW1 | 0.038 | 1 |
| RulB_PaW1 | 0.071 | 1 |
| RulB_PaW1 | 0.056 | 1 |
| RulB_PaW1 | 0.040 | 1 |
| RulB_PaW1 | 0.092 | 1 |
| RulB_PaW1 | 0.107 | 1 |
| RulB_PaW1 | 0.155 | 1 |
| RulB_PaW1 | 0.099 | 1 |
| RulB_PaW1 | 0.061 | 1 |
| RulB_PaW1 | 0.049 | 1 |
| RulB_PaW1 | 0.036 | 1 |
| RulB_PaW1 | 0.051 | 1 |
| RulB_PaW1 | 0.490 | 2 |
| RulB_PaW1 | 0.830 | 2 |
| RulB_PaW1 | 0.825 | 2 |
| RulB_PaW1 | 0.822 | 2 |
| RulB_PaW1 | 1.312 | 2 |
| RulB_PaW1 | 1.587 | 2 |
| RulB_PaW1 | 1.700 | 2 |
| RulB_PaW1 | 1.823 | 2 |
| RulB_PaW1 | 0.456 | 2 |
| RulB_PaW1 | 0.847 | 2 |
| RulB_PaW1 | 0.776 | 2 |
| DnaE2_PC20 | 0.035 | 1 |
| DnaE2_PC20 | 0.050 | 1 |
| DnaE2_PC20 | 0.038 | 1 |
| DnaE2_PC20 | 0.109 | 1 |
| DnaE2_PC20 | 0.077 | 1 |
| DnaE2_PC20 | 0.066 | 1 |
| DnaE2_PC20 | 0.096 | 1 |
| DnaE2_PC20 | 0.051 | 1 |
| DnaE2_PC20 | 0.053 | 1 |
| DnaE2_PC20 | 0.040 | 1 |
| DnaE2_PC20 | 0.034 | 1 |
| DnaE2_PC20 | 0.037 | 1 |
| DnaE2_PC20 | 0.052 | 1 |
| DnaE2_PC20 | 0.059 | 1 |
| DnaE2_PC20 | 0.058 | 1 |
| DnaE2_PC20 | 0.086 | 2 |
| DnaE2_PC20 | 0.091 | 2 |
| DnaE2_PC20 | 0.069 | 2 |
| DnaE2_PC20 | 0.115 | 2 |
| DnaE2_PC20 | 0.095 | 2 |
| DnaE2_PC20 | 0.082 | 2 |
| DnaE2_PC20 | 0.054 | 2 |
| DnaE2_PC20 | 0.063 | 2 |
| DnaE2_PC20 | 0.063 | 2 |
| DnaE2_PC20 | 0.047 | 2 |
| DnaE2_PC20 | 0.078 | 2 |
| DnaE2_PC20 | 0.067 | 2 |
| DnaE2_PC20 | 0.089 | 2 |
| 2063_PC20 | 0.130 | 1 |
| 2063_PC20 | 0.187 | 1 |
| 2063_PC20 | 0.172 | 1 |
| 2063_PC20 | 0.264 | 1 |
| 2063_PC20 | 0.262 | 1 |
| 2063_PC20 | 0.277 | 1 |
| 2063_PC20 | 0.207 | 1 |
| 2063_PC20 | 0.181 | 1 |
| 2063_PC20 | 0.213 | 1 |
| 2063_PC20 | 0.162 | 1 |
| 2063_PC20 | 0.175 | 1 |
| 2063_PC20 | 0.207 | 1 |
| 2063_PC20 | 0.240 | 1 |
| 2063_PC20 | 0.237 | 1 |
| 2063_PC20 | 0.216 | 1 |
| 2063_PC20 | 0.719 | 2 |
| 2063_PC20 | 0.816 | 2 |
| 2063_PC20 | 0.814 | 2 |
| 2063_PC20 | 0.964 | 2 |
| 2063_PC20 | 1.201 | 2 |
| 2063_PC20 | 1.140 | 2 |
| 2063_PC20 | 0.807 | 2 |
| 2063_PC20 | 1.028 | 2 |
| 2063_PC20 | 0.787 | 2 |
| 2063_PC20 | 0.786 | 2 |
| 2063_PC20 | 1.195 | 2 |
| 2063_PC20 | 0.990 | 2 |
| 2063_PC20 | 1.044 | 2 |
| pG20_PC20 | 0.608 | 1 |
| pG20_PC20 | 0.428 | 1 |
| pG20_PC20 | 0.427 | 1 |
| pG20_PC20 | 0.407 | 1 |
| pG20_PC20 | 1.297 | 1 |
| pG20_PC20 | 1.328 | 1 |
| pG20_PC20 | 1.462 | 1 |
| pG20_PC20 | 1.628 | 1 |
| pG20_PC20 | 0.359 | 1 |
| pG20_PC20 | 0.398 | 1 |
| pG20_PC20 | 0.861 | 1 |
| pG20_PC20 | 0.619 | 1 |
| pG20_PC20 | 4.871 | 2 |
| pG20_PC20 | 5.097 | 2 |
| pG20_PC20 | 3.667 | 2 |
| pG20_PC20 | 3.595 | 2 |
| pG20_PC20 | 3.352 | 2 |
| pG20_PC20 | 2.900 | 2 |
| pG20_PC20 | 2.728 | 2 |
| pG20_PC20 | 2.625 | 2 |
| pG20_PC20 | 2.559 | 2 |
| pG20_PC20 | 1.790 | 2 |
| pG20_PC20 | 4.332 | 2 |
| pG20_PC20 | 5.300 | 2 |
| pG20_PC20 | 4.537 | 2 |
| 2082_PC20 | 0.217 | 1 |
| 2082_PC20 | 0.225 | 1 |
| 2082_PC20 | 0.209 | 1 |
| 2082_PC20 | 0.316 | 1 |
| 2082_PC20 | 0.296 | 1 |
| 2082_PC20 | 0.330 | 1 |
| 2082_PC20 | 0.303 | 1 |
| 2082_PC20 | 0.354 | 1 |
| 2082_PC20 | 0.341 | 1 |
| 2082_PC20 | 0.297 | 1 |
| 2082_PC20 | 0.201 | 1 |
| 2082_PC20 | 0.230 | 1 |
| 2082_PC20 | 0.203 | 1 |
| 2082_PC20 | 0.177 | 1 |
| 2082_PC20 | 0.747 | 2 |
| 2082_PC20 | 0.616 | 2 |
| 2082_PC20 | 0.620 | 2 |
| 2082_PC20 | 0.958 | 2 |
| 2082_PC20 | 1.005 | 2 |
| 2082_PC20 | 1.378 | 2 |
| 2082_PC20 | 0.632 | 2 |
| 2082_PC20 | 0.937 | 2 |
| 2082_PC20 | 0.380 | 2 |
| 2082_PC20 | 0.569 | 2 |
| 2082_PC20 | 0.754 | 2 |
| 2082_PC20 | 0.947 | 2 |
| 2082_PC20 | 0.286 | 2 |
| 1_PC24 | 0.651 | 1 |
| 1_PC24 | 0.572 | 1 |
| 1_PC24 | 0.751 | 1 |
| 1_PC24 | 0.815 | 1 |
| 1_PC24 | 0.972 | 1 |
| 1_PC24 | 1.147 | 1 |
| 1_PC24 | 1.005 | 1 |
| 1_PC24 | 1.439 | 1 |
| 1_PC24 | 1.105 | 1 |
| 1_PC24 | 0.993 | 1 |
| 1_PC24 | 1.036 | 1 |
| 1_PC24 | 1.054 | 1 |
| 1_PC24 | 1.337 | 1 |
| 1_PC24 | 1.113 | 1 |
| 1_PC24 | 1.352 | 1 |
| 1_PC24 | 1.010 | 1 |
| 1_PC24 | 1.786 | 2 |
| 1_PC24 | 2.812 | 2 |
| 1_PC24 | 3.244 | 2 |
| 1_PC24 | 4.290 | 2 |
| 1_PC24 | 2.820 | 2 |
| 1_PC24 | 3.211 | 2 |
| 1_PC24 | 3.512 | 2 |
| 1_PC24 | 2.916 | 2 |
| 1_PC24 | 2.800 | 2 |
| 1_PC24 | 3.248 | 2 |
| 1_PC24 | 1.284 | 2 |
| 1_PC24 | 3.194 | 2 |
| 1_PC24 | 4.177 | 2 |
| 1_PC24 | 3.516 | 2 |
| 1_PC24 | 4.129 | 2 |
| 1_PC24 | 2.691 | 2 |
| 438_PC24 | 0.108 | 1 |
| 438_PC24 | 0.190 | 1 |
| 438_PC24 | 0.128 | 1 |
| 438_PC24 | 0.126 | 1 |
| 438_PC24 | 0.129 | 1 |
| 438_PC24 | 0.104 | 1 |
| 438_PC24 | 0.156 | 1 |
| 438_PC24 | 0.105 | 1 |
| 438_PC24 | 0.088 | 1 |
| 438_PC24 | 0.035 | 1 |
| 438_PC24 | 0.097 | 1 |
| 438_PC24 | 0.093 | 1 |
| 438_PC24 | 0.097 | 1 |
| 438_PC24 | 0.117 | 1 |
| 438_PC24 | 0.119 | 1 |
| 438_PC24 | 0.117 | 1 |
| 438_PC24 | 0.746 | 2 |
| 438_PC24 | 0.797 | 2 |
| 438_PC24 | 0.892 | 2 |
| 438_PC24 | 0.728 | 2 |
| 438_PC24 | 0.653 | 2 |
| 438_PC24 | 1.181 | 2 |
| 438_PC24 | 1.073 | 2 |
| 438_PC24 | 1.269 | 2 |
| 438_PC24 | 0.696 | 2 |
| 438_PC24 | 0.654 | 2 |
| 438_PC24 | 0.774 | 2 |
| 438_PC24 | 0.563 | 2 |
| 438_PC24 | 1.691 | 2 |
| 438_PC24 | 1.413 | 2 |
| 438_PC24 | 1.657 | 2 |
| 438_PC24 | 1.146 | 2 |
| DnaE2_PC24 | 0.044 | 1 |
| DnaE2_PC24 | 0.033 | 1 |
| DnaE2_PC24 | 0.016 | 1 |
| DnaE2_PC24 | 0.031 | 1 |
| DnaE2_PC24 | 0.043 | 1 |
| DnaE2_PC24 | 0.042 | 1 |
| DnaE2_PC24 | 0.041 | 1 |
| DnaE2_PC24 | 0.062 | 1 |
| DnaE2_PC24 | 0.009 | 1 |
| DnaE2_PC24 | 0.014 | 1 |
| DnaE2_PC24 | 0.008 | 1 |
| DnaE2_PC24 | 0.010 | 1 |
| DnaE2_PC24 | 0.020 | 1 |
| DnaE2_PC24 | 0.016 | 1 |
| DnaE2_PC24 | 0.022 | 1 |
| DnaE2_PC24 | 0.021 | 1 |
| DnaE2_PC24 | 0.077 | 2 |
| DnaE2_PC24 | 0.094 | 2 |
| DnaE2_PC24 | 0.093 | 2 |
| DnaE2_PC24 | 0.088 | 2 |
| DnaE2_PC24 | 0.140 | 2 |
| DnaE2_PC24 | 0.117 | 2 |
| DnaE2_PC24 | 0.096 | 2 |
| DnaE2_PC24 | 0.105 | 2 |
| DnaE2_PC24 | 0.067 | 2 |
| DnaE2_PC24 | 0.059 | 2 |
| DnaE2_PC24 | 0.039 | 2 |
| DnaE2_PC24 | 0.066 | 2 |
| DnaE2_PC24 | 0.090 | 2 |
| DnaE2_PC24 | 0.089 | 2 |
| DnaE2_PC24 | 0.062 | 2 |
| DnaE2_PC24 | 0.087 | 2 |

The effect of aromatic compounds (2.5 mM m-cresol or 5 mM phenol with exception of PC20 for which we used 2.5 mM phenol and 1.25 mM m-cresol) to the appearance frequency of Rif^r^ mutants in rich medium (M9 medium supplemented with glucose (0.2%) and CAA (0.2%)).

| Strain | The frequency of Rif^r^ colonies | | |
| --- | --- | --- | --- |
|  | Glc + CAA | Glc + CAA + Phe | Glc + CAA + m-Cre |
| PaW85 | 5.20E-09 | 6.25E-10 | 1.09E-08 |
| PaW85 | 6.67E-09 | 1.00E-08 | 1.09E-08 |
| PaW85 | 8.94E-09 | 8.75E-09 | 1.09E-08 |
| PaW85 | 5.37E-09 | 2.50E-09 | 1.63E-08 |
| PaW85 | 5.20E-09 | 2.50E-09 | 0.00E+00 |
| PaW85 | 7.15E-09 | 6.25E-09 | 1.09E-08 |
| PaW85 | 1.11E-08 | 1.06E-08 | 1.63E-08 |
| PaW85 | 9.92E-09 | 9.38E-09 | 2.72E-08 |
| PaW85 | 2.37E-08 | 1.25E-09 | 1.09E-08 |
| PaW85 | 1.07E-08 | 2.03E-09 | 5.13E-09 |
| PaW85 | 1.32E-08 | 6.09E-09 | 5.13E-09 |
| PaW85 | 2.11E-08 | 2.44E-08 | 0.00E+00 |
| PaW85 | 4.29E-09 | 5.08E-09 | 0.00E+00 |
| PaW85 | 4.63E-09 | 1.12E-08 | 5.13E-09 |
| PaW85 | 4.75E-09 | 3.76E-08 | 5.13E-08 |
| PaW85 | 8.47E-09 | 1.02E-09 | 1.03E-08 |
| PaW85 | 3.36E-08 | 7.11E-09 | 4.62E-08 |
| PaW85 | 8.93E-09 | 1.83E-08 | 4.62E-08 |
| PaW85 | 9.04E-09 | 7.11E-09 | 5.60E-08 |
| PaW85 | 8.25E-09 | 6.86E-09 | 1.07E-08 |
| PaW85 | 2.08E-08 | 5.26E-08 | 5.33E-08 |
| PaW85 | 1.23E-08 | 5.71E-10 | 1.20E-08 |
| PaW85 | 2.33E-08 | 5.71E-10 | 2.00E-08 |
| PaW85 | 7.50E-09 | 6.29E-09 | 1.33E-09 |
| PaW85 | 1.30E-08 | 5.71E-10 | 9.33E-09 |
| PaW85 | 3.95E-08 | 6.86E-09 | 1.07E-08 |
| PaW85 | 2.85E-08 | 1.14E-08 | 2.27E-08 |
| PaW85 | 1.13E-08 | 1.09E-08 | 9.20E-08 |
| PaWpheBA25 | 7.73E-09 | 6.27E-09 | 7.30E-09 |
| PaWpheBA25 | 3.33E-09 | 3.83E-08 | 0.00E+00 |
| PaWpheBA25 | 9.07E-09 | 1.20E-08 | 3.50E-08 |
| PaWpheBA25 | 1.32E-08 | 1.91E-08 | 2.63E-08 |
| PaWpheBA25 | 6.00E-09 | 1.22E-08 | 2.19E-08 |
| PaWpheBA25 | 5.87E-09 | 1.07E-08 | 4.82E-08 |
| PaWpheBA25 | 4.67E-09 | 1.45E-08 | 3.80E-08 |
| PaWpheBA25 | 2.53E-08 | 3.86E-08 | 3.80E-08 |
| PaWpheBA25 | 4.80E-09 | 7.32E-09 | 4.96E-08 |
| PaWpheBA25 | 1.78E-08 | 9.41E-09 | 2.92E-09 |
| PaWpheBA25 | 1.76E-08 | 1.20E-08 | 1.01E-08 |
| PaWpheBA25 | 1.35E-08 | 8.25E-09 | 1.56E-09 |
| PaWpheBA25 | 1.26E-08 | 1.15E-08 | 9.87E-09 |
| PaWpheBA25 | 1.48E-08 | 1.71E-08 | 2.16E-08 |
| PaWpheBA25 | 1.95E-08 | 1.46E-08 | 1.40E-08 |
| PaWpheBA25 | 2.14E-08 | 1.02E-08 | 1.30E-09 |
| PaWpheBA25 | 1.99E-08 | 1.47E-08 | 1.32E-08 |
| PaWpheBA25 | 1.52E-08 | 7.57E-09 | 5.97E-09 |
| PaWpheBA25 | 2.53E-08 | 9.38E-09 | 3.12E-09 |
| PaWpheBA25 | 3.64E-08 | 2.93E-08 | 1.61E-08 |
| PaWpheBA25 | 7.56E-09 | 2.70E-08 | 1.02E-08 |
| PaWpheBA25 | 2.27E-08 | 1.52E-08 | 2.68E-08 |
| PaWpheBA25 | 3.36E-08 | 2.21E-08 | 3.21E-08 |
| PaWpheBA25 | 2.40E-08 | 2.10E-08 | 1.34E-08 |
| PaWpheBA25 | 2.91E-08 | 2.65E-08 | 7.05E-09 |
| PaWpheBA25 | 1.29E-08 | 4.66E-08 | 1.41E-08 |
| PaWpheBA25 | 1.24E-08 | 2.10E-08 | 8.11E-09 |
| PaWpheBA25 | 1.67E-08 | 9.92E-09 | 1.09E-08 |
| PaWpheBA25 | | 4.64E-08 | 1.13E-08 |
| PaWrulAB | 2.17E-08 | 1.05E-08 | 6.97E-10 |
| PaWrulAB | 1.33E-08 | 1.20E-08 | 8.36E-09 |
| PaWrulAB | 2.43E-08 | 8.00E-09 | 8.36E-09 |
| PaWrulAB | 1.66E-08 | 8.50E-09 | 4.88E-09 |
| PaWrulAB | 1.18E-08 | 3.05E-08 | 1.11E-08 |
| PaWrulAB | 1.44E-08 | 3.00E-09 | 0.00E+00 |
| PaWrulAB | 1.55E-08 | 5.50E-09 | 3.48E-09 |
| PaWrulAB | 3.08E-08 | 2.85E-08 | 1.05E-08 |
| PaWrulAB | 1.08E-08 | 1.80E-08 | 1.67E-08 |
| PaWrulAB | 1.47E-08 | 1.15E-08 | 0.00E+00 |
| PaWrulAB | 9.30E-09 | 5.76E-09 | 1.21E-09 |
| PaWrulAB | 9.30E-09 | 7.41E-09 | 1.82E-09 |
| PaWrulAB | 8.34E-09 | 4.94E-09 | 1.21E-08 |
| PaWrulAB | 1.96E-08 | 6.86E-09 | 2.42E-09 |
| PaWrulAB | 2.67E-09 | 1.54E-08 | 4.85E-09 |
| PaWrulAB | 8.56E-09 | 3.07E-08 | 5.45E-09 |
| PaWrulAB | 1.18E-08 | 4.39E-09 | 6.06E-10 |
| PaWrulAB | 1.63E-08 | 8.81E-08 | 4.24E-09 |
| PaWrulAB | 1.22E-08 | 3.16E-08 | 9.70E-09 |
| PaWrulAB | 1.41E-08 | 2.26E-08 | 2.55E-08 |
| PaWrulAB | 1.20E-08 | 1.64E-08 | 2.94E-08 |
| PaWrulAB | 3.32E-08 | 1.87E-08 | 4.16E-08 |
| PaWrulAB | 1.20E-08 | 4.44E-08 | 3.55E-08 |
| PaWrulAB | 9.43E-09 | 3.20E-08 | 2.94E-08 |
| PaWrulAB | 2.53E-08 | 3.90E-09 | 6.09E-09 |
| PaWrulAB | 4.31E-08 | 1.56E-08 | 3.25E-08 |
| PaWrulAB | 8.79E-09 | 5.46E-09 | 1.52E-08 |
| PaWrulAB | 1.82E-08 | 3.90E-09 | 2.94E-08 |
| PaWrulAB | |  | 3.96E-08 |
| PaWrulAB | |  | 6.09E-08 |
| PC20 | 2.02E-08 | 1.26E-09 | 4.29E-09 |
| PC20 | 5.69E-09 | 8.53E-09 | 4.86E-09 |
| PC20 | 9.48E-10 | 9.48E-10 | 3.71E-09 |
| PC20 | 7.27E-09 | 7.90E-09 | 1.43E-09 |
| PC20 | 6.64E-09 | 3.48E-09 | 8.29E-09 |
| PC20 | 4.74E-09 | 4.42E-09 | 2.00E-09 |
| PC20 | 2.21E-09 | 2.53E-09 | 5.71E-10 |
| PC20 | 1.90E-09 | 4.11E-09 | 2.00E-09 |
| PC20 | 1.26E-08 | 2.53E-09 | 1.14E-09 |
| PC20 | 4.42E-09 | 6.00E-09 | 5.46E-10 |
| PC20 | 1.16E-08 | 1.62E-08 | 1.64E-09 |
| PC20 | 2.19E-08 | 1.34E-08 | 5.73E-09 |
| PC20 | 1.23E-08 | 1.57E-08 | 4.09E-09 |
| PC20 | 1.55E-08 | 4.62E-09 | 4.37E-09 |
| PC20 | 1.35E-08 | 1.39E-09 | 4.09E-09 |
| PC20 | 9.68E-09 | 6.00E-09 | 3.27E-09 |
| PC20 | 3.48E-08 | 1.62E-08 | 3.55E-09 |
| PC20 | 1.74E-08 | 5.54E-09 | 1.64E-09 |
| PC20 | 3.23E-09 | 7.39E-09 | 7.09E-09 |
| PC20 | 5.35E-09 | 7.39E-09 | 2.14E-09 |
| PC20 | 6.88E-09 | 3.48E-09 | 1.07E-08 |
| PC20 | 1.34E-08 | 3.16E-10 | 8.57E-10 |
| PC20 | 1.99E-08 | 5.69E-09 | 2.14E-09 |
| PC20 | 9.18E-09 | 1.90E-09 | 1.71E-09 |
| PC20 | 3.44E-09 | 3.48E-09 | 8.57E-10 |
| PC20 | 1.49E-08 | 6.32E-09 | 4.71E-09 |
| PC20 | 1.34E-08 | 2.84E-09 | 1.71E-09 |
| PC20 | 9.18E-09 | 6.95E-09 | 8.57E-10 |
| PC20 |  | 5.06E-09 |  |
| PC24 | 6.62E-09 | 4.52E-09 | 5.08E-09 |
| PC24 | 4.00E-09 | 2.94E-09 | 2.54E-08 |
| PC24 | 2.00E-09 | 4.07E-09 | 1.83E-08 |
| PC24 | 1.43E-08 | 5.20E-09 | 5.08E-09 |
| PC24 | 4.77E-09 | 2.60E-09 | 7.01E-08 |
| PC24 | 3.54E-09 | 7.57E-09 | 3.05E-08 |
| PC24 | 3.69E-09 | 9.72E-09 | 1.62E-08 |
| PC24 | 1.11E-08 | 4.52E-09 | 1.02E-09 |
| PC24 | 7.23E-09 | 4.97E-09 | 7.11E-09 |
| PC24 | 3.65E-08 | 4.63E-09 | 8.37E-09 |
| PC24 | 3.95E-09 | 9.12E-09 | 2.28E-09 |
| PC24 | 2.18E-08 | 6.26E-09 | 1.22E-08 |
| PC24 | 9.36E-09 | 5.31E-09 | 1.29E-08 |
| PC24 | 5.41E-09 | 2.19E-08 | 3.04E-09 |
| PC24 | 5.32E-09 | 4.35E-09 | 8.37E-09 |
| PC24 | 3.69E-09 | 6.67E-09 | 1.83E-08 |
| PC24 | 4.89E-09 | 6.80E-09 | 3.80E-09 |
| PC24 | 3.26E-09 | 8.16E-09 | 1.67E-08 |
| PC24 | 8.58E-09 | 8.30E-09 | 4.56E-09 |
| PC24 | 1.11E-08 | 5.85E-09 | 1.67E-08 |
| PC24 | 8.93E-09 | 5.17E-09 | 7.67E-09 |
| PC24 | 7.73E-09 | 6.57E-09 | 4.88E-09 |
| PC24 | 8.40E-09 | 5.31E-09 | 6.97E-10 |
| PC24 | 7.47E-09 | 9.37E-09 | 1.53E-08 |
| PC24 | 1.87E-08 | 4.34E-09 | 9.76E-09 |
| PC24 | 1.25E-08 | 4.06E-09 | 6.97E-09 |
| PC24 | 1.13E-08 | 9.23E-09 | 9.06E-09 |
| PC24 | 1.24E-08 | 4.90E-09 | 9.76E-09 |
| PC24 | 3.57E-08 | 7.27E-09 | 2.86E-08 |
| PC24 |  | 4.34E-09 |  |

The frequency of Rif^r^ mutants grown in M9 minimal medium supplemented with either glucose (0.2%), phenol (2.5 mM) or p-cresol (1.25 mM) as a sole carbon and energy source.

| Strain | The frequency of Rifr colonies | | |
| --- | --- | --- | --- |
|  | Glucose | Phenol | p-Cresol |
| PaWpheBA25 | 4.5E-09 | 1.2E-08 |  |
| PaWpheBA25 | 3.25E-09 | 7.86E-09 |  |
| PaWpheBA25 | 3.43E-09 | 5.22E-08 |  |
| PaWpheBA25 | 7E-09 | 1.44E-07 |  |
| PaWpheBA25 | 6.67E-09 | 1.32E-07 |  |
| PaWpheBA25 | 2.67E-09 | 1.2E-08 |  |
| PaWpheBA25 | 5E-09 | 4E-09 |  |
| PaWpheBA25 | 5.25E-09 | 3.4E-08 |  |
| PaWpheBA25 | 1.7E-08 | 3.85E-08 |  |
| PaWpheBA25 | 6.67E-09 | 1.75E-08 |  |
| PaWpheBA25 | 2.64E-08 | 1.24E-07 |  |
| PaWpheBA25 | 2.86E-08 | 1.49E-07 |  |
| PaWpheBA25 | 2.16E-08 | 9.36E-08 |  |
| PaWpheBA25 | 1.49E-08 | 6.86E-08 |  |
| PaWpheBA25 | 1.56E-08 | 5.41E-07 |  |
| PaWpheBA25 | 1.27E-08 | 4.57E-07 |  |
| PaWpheBA25 | 1.89E-08 | 1.18E-08 |  |
| PaWpheBA25 | 1.72E-08 | 6.67E-08 |  |
| PaWpheBA25 | 1.24E-08 | 2E-08 |  |
| PC20 | 2E-08 | 2.54E-08 |  |
| PC20 | 7.67E-08 | 1.8E-08 |  |
| PC20 | 6.29E-08 | 2.36E-08 |  |
| PC20 | 9.11E-08 | 1.64E-08 |  |
| PC20 | 7.82E-08 | 1.38E-08 |  |
| PC20 | 6.8E-08 | 1.42E-08 |  |
| PC20 | 2.5E-08 | 4.33E-09 |  |
| PC20 | 5.14E-08 | 5.27E-09 |  |
| PC20 | 5.71E-09 | 1.63E-08 |  |
| PC20 | 2.5E-08 | 1.86E-08 |  |
| PC20 | 2.25E-08 | 1.14E-07 |  |
| PC20 | 6.6E-08 | 6.67E-08 |  |
| PC20 | 2E-08 | 5.96E-08 |  |
| PC20 | 6E-08 | 8.1E-08 |  |
| PC20 | 2.8E-08 | 8.25E-08 |  |
| PC20 | 1.03E-07 | 4.65E-08 |  |
| PC20 | 5.33E-08 | 1.71E-08 |  |
| PC20 | 6.32E-08 | 3.06E-08 |  |
| PC20 | 7.16E-08 | 2.43E-08 |  |
| PC20 | 4.43E-08 | 1.83E-08 |  |
| PC24 | 2.35E-07 | 2.48E-07 | 1.19E-07 |
| PC24 | 1.36E-07 | 2.27E-07 | 4.89E-08 |
| PC24 | 3.64E-07 | 2.5E-07 | 6.43E-08 |
| PC24 | 2.84E-07 | 9.5E-08 | 1.17E-07 |
| PC24 | 2.66E-07 | 2.08E-07 | 7.4E-08 |
| PC24 | 2.17E-08 | 1.7E-08 | 1.14E-08 |
| PC24 | 2.36E-08 | 1.2E-08 | 4.5E-09 |
| PC24 | 6.29E-09 | 8.74E-09 | 1.12E-08 |
| PC24 | 4.33E-09 | 1.27E-08 | 7.75E-09 |
| PC24 | 1.5E-08 | 1.04E-08 | 1.7E-08 |
| PC24 | 1.14E-08 | 7.78E-09 | 6.57E-09 |
| PC24 | 1E-08 | 1E-08 | 6.4E-09 |
| PC24 | 2.06E-08 | 4.36E-08 | 9.2E-09 |
| PC24 | 1.17E-08 | 8.92E-09 | 3.71E-09 |
| PC24 | 7E-09 | 9.6E-09 | 6E-09 |
| PC24 | 4.4E-08 | 8.73E-09 | 2.15E-08 |
| PC24 | 5.87E-08 | 1.13E-07 | 1.44E-08 |
| PC24 | 2.33E-08 | 2.06E-08 | 4.45E-08 |
| PC24 | 2.16E-08 | 1.54E-08 | 1.42E-08 |
| PC24 | 4.6E-08 | 1.18E-07 | 1.4E-08 |
