# Supplementary material for: Contribution of increased mutagenesis to the evolution of pollutants-degrading indigenous bacteria
Source: PLoS One. 2017 Aug 4;12(8):e0182484. doi: 10.1371/journal.pone.0182484 (PMC5544203; doi:10.1371/journal.pone.0182484)
Supplement: S2 Fig — Black boxes indicate the putative active sites residues according to NCBI Conserved Protein Domain Family database (source pfam; superfamily cl10465; Peptidase_S24_S26; [95]. Residues shaded orange represent the autocatalytic cleavage site shown to be required for posttranslational processing of UmuD/RulA homologs [96, 97]. Residues surrounded by pink boxes have been shown to be important in the UmuDC mediated mutagenesis [98]. Sequences were aligned with ClustalX2 and domains were marked according to Protein sequence analysis and classification portal Interpro (http://www.ebi.ac.uk/interpro/) based on the sequence of UmuD (P0AG11). (PDF) [file pone.0182484.s003.pdf]

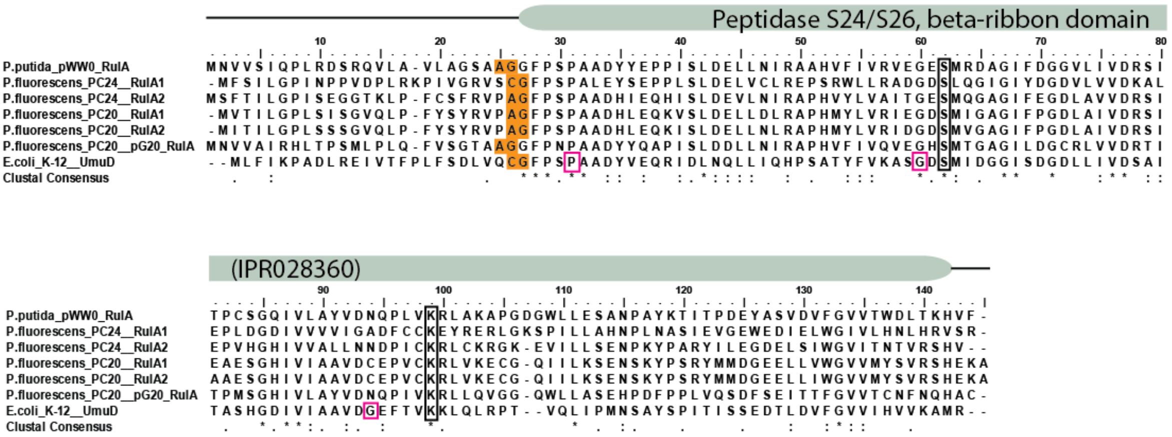

S2 Figure. Multiple sequence alignment of RulA homologs from *Pseudomonas fluorescens* PC20, PC24; *Pseudomonas putida* plasmid pWW0 RulA (Q8VMP5) and *E. coli* K-12 UmuD (P0AG11). Black boxes indicate the putative active sites residues according to NCBI Conserved Protein Domain Family database (source pfam; superfamily cl10465; Peptidase_S24_S26; [1]. Residues shaded orange represent the autocatalytic cleavage site shown to be required for posttranslational processing of UmuD/RulA homologs [2,3]. Residues surrounded by pink boxes[1] have been shown to be important in the UmuDC mediated mutagenesis [4]. Sequences were aligned with ClustalX2 and domains were marked according to Protein sequence analysis and classification portal Interpro (http://www.ebi.ac.uk/interpro/) based on the sequence of UmuD (P0AG11).

1. Marchler-Bauer A, Derbyshire MK, Gonzales NR, Lu S, Chitsaz F, Geer LY, et al. (2015) CDD: NCBI's conserved domain database. Nucleic Acids Res 43: D222-226.
2. Nohmi T, Battista JR, Dodson LA, Walker GC (1988) RecA-mediated cleavage activates UmuD for mutagenesis: mechanistic relationship between transcriptional derepression and posttranslational activation. Proc Natl Acad Sci U S A 85: 1816-1820.
3. Sundin GW, Kidambi SP, Ullrich M, Bender CL (1996) Resistance to ultraviolet light in *Pseudomonas syringae*: sequence and functional analysis of the plasmid-encoded *rulAB* genes. Gene 177: 77-81.
4. Koch WH, Ennis DG, Levine AS, Woodgate R (1992) *Escherichia coli umuDC* mutants: DNA sequence alterations and UmuD cleavage. Mol Gen Genet 233: 443-448.
